# The role of serum amyloid A1 in the adipogenic differentiation of human adipose-derived stem cells basing on single-cell RNA sequencing analysis

**DOI:** 10.1186/s13287-022-02873-5

**Published:** 2022-05-07

**Authors:** Rongmei Qu, Kai He, Yuchao Yang, Tingyu Fan, Bing Sun, Asmat Ullah Khan, Wenhua Huang, Jun Ouyang, Xinghua Pan, Jingxing Dai

**Affiliations:** 1 Guangdong Provincial Key Laboratory of Medical Biomechanics and Guangdong Engineering Research Center for Translation of Medical 3D Printing Application and National Key Discipline of Human Anatomy, School of Basic Medical Science, Southern Medical University and National Demonstration Center for Experimental Education of Basic Medical Sciences, Southern Medical University, Guangzhou, China; 2 Guangdong Provincial Key Lab of Single Cell Technology and Application, and Department of Biochemistry and Molecular Biology, School of Basic Medical Science, Southern Medical University, Guangzhou, China

**Keywords:** Adipose-derived stem cells (ASCs), Adipogenesis, Serum amyloid A1 (SAA1), Regulation of inflammatory response, Single-cell transcriptomic sequencing (scRNA-seq)

## Abstract

**Background:**

Adipose-derived stem cells (ASCs) are obtained from a variety of sources in vivo where they present in large quantities. These cells are suitable for use in autologous transplantation and the construction of tissue-engineered adipose tissue. Studies have shown that ASCs differentiation is in a high degree of heterogeneity, yet the molecular basis including key regulators of differentiation remains to clarify.

**Methods:**

We performed single-cell RNA sequencing and bioinformatics analysis on both undifferentiated (ASC-GM group) and adipogenically differentiated human ASCs (ASC-AD group, ASCs were cultured in adipogenic inducing medium for 1 week). And then, we verified the results of serum amyloid A1 (SAA1) with western blotting, immunofluorescence staining, oil red O staining. After these experiments, we down-regulated the expression of serum amyloid A1 (SAA1) gene to verify the adipogenic differentiation ability of ASCs.

**Results:**

In single-cell RNA sequence analyzing, we obtained 4415 cells in the ASC-GM group and 4634 cells in the ASC-AD group. The integrated sample cells could be divided into 11 subgroups (0–10 cluster). The cells in cluster 0, 2, 5 were came from ASC-GM group and the cells in cluster 1, 3, 7 came from ASC-AD group. The cells of cluster 4 and 6 came from both ASC-GM and ASC-AD groups. Fatty acid binding protein 4, fatty acid binding protein 5, complement factor D, fatty acid desaturase 1, and insulin like growth factor binding protein 5 were high expressed in category 1 and 7. Regulation of inflammatory response is the rank 1 biological processes. And cellular responses to external stimuli, negative regulation of defense response and acute inflammatory response are included in top 20 biological processes. Based on the MCODE results, we found that SAA1, C-C Motif Chemokine Ligand 5 (CCL5), and Annexin A1 (ANXA1) significantly highly expressed during adipogenic differentiation. Western blot and immunofluorescent staining results showed that SAA1 increased during adipogenesis. And the area of ORO positive staining in siSAA1 cells was significantly lower than in the siControl (negative control) cells.

**Conclusions:**

Our results also indicated that our adipogenic induction was successful, and there was great heterogeneity in the adipogenic differentiation of ASCs. SAA1 with the regulation of inflammatory response were involved in adipogenesis of ASCs based on single-cell RNA sequencing analysis. The data obtained will help to elucidate the intrinsic mechanism of heterogeneity in the differentiation process of stem cells, thus, guiding the regulation of self-renewal and differentiation of adult stem cells.

**Supplementary Information:**

The online version contains supplementary material available at 10.1186/s13287-022-02873-5.

## Introduction

Adipose-derived stem cells (ASCs) are available from the clearance of fat tissue around the vascular system and the suction of fat content during liposuction surgery. ASCs can differentiate into osteoblasts, chondrocytes, adipocytes, and many other cell types [1, 2]. There has been an increasing focus on the use of ASCs as seed cells for the construction of tissue-engineered fat and autologous transplantation. This interest is partially attributable to the large pool of ASCs that can be isolated in vivo [3]. A better understanding of the mechanisms that regulate ASC differentiation and the development of adipose tissue is essential. It will advance our understanding of the physiological conditions that influence fat production. Knowledge of these conditions will not only aid in the development of clinically relevant products for mass soft tissue repair but also potential therapies to prevent obesity and its harmful consequences [3, 4].

Both in vivo and in vitro studies have shown that stem cells are heterogeneous populations of cells with the potential for self-renewal and differentiation. Although these properties are rarely studied in mature tissues, they are the basis of the unique differentiation and regeneration abilities seen in adult stem cells [5–7]. Increasing evidences show that the molecular mechanisms involved in stem cell self-renewal and differentiation, as well as the generation of diseases related to stem cell dysfunction, all occur at the single-cell level [8]. Current research studies based on heterogeneous stem cell populations have considerable limitations. In recent years, the development of single-cell RNA sequencing technology has provided a foundation for the study of heterogeneous cell populations at the ultimate resolution.

Stem cell differentiation is a very complex biological process controlled by the coordination of multiple gene networks. Studies have shown that most genes show a high degree of intercellular heterogeneity during stem cell differentiation [9–11] (even the critical regulators of differentiation). Most experimental methods have failed to elucidate the intrinsic mechanism of heterogeneity in stem cell differentiation. This stem cell heterogeneity can be interpreted by single-cell RNA sequencing [12].

We performed single-cell RNA sequencing on undifferentiated and adipogenic differentiated human ASCs to understand the intrinsic mechanism of heterogeneity in the differentiation of stem cells. Knowledge of this mechanism will help to guide the regulation of self-renewal and differentiation of adult stem cells. One crucial biological process (regulation of inflammatory response) was identified, and we believe that changes in serum amyloid A1 (*SAA1*) [13, 14] may be a critical regulatory factor in the adipogenic differentiation of ASCs.

## Materials and methods

### Ethical approval

The patients/participants provided their written informed consent to participate in this study. This study was approved by the ethics committee of the School of Basic Medical Sciences, Southern Medical University, and all protocols agreed with the ethical standards of the research committee. Adipose tissue from the participants was provided by the Department of Plastic Surgery, Nanfang Hospital, Southern Medical University, China.

### Cell culture

We obtained ASCs from human adipose tissue using the type I collagenase digestion method as described previously [15, 16]. The liposuction sample from one participant was used in AD group and GM group for single-cell RNA sequencing, and the body mass index of the participant was 24.6. The liposuction sample was digested with 0.15% type 1 collagenase (Sigma, Saint Louis, MO, USA) for 40 min. The reaction was terminated with a growth medium (GM), and the samples were then centrifuged at 800 rpm for 5 min. The GM contained Dulbecco’s modified Eagle’s medium (DMEM) with 10% fetal bovine serum (FBS; Gibco; Carlsbad, CA; USA) and 1% penicillin/streptomycin (Gibco). The cells were planted in a 10 cm dish, incubated in a 37 °C incubator with 5% CO_2_, and the medium was replaced 24 h later. Cells cultured for 3–6 passages were used for seeding a 10 cm dish at a density of 8000 cells/cm^2^ when cell confluence was approximately 80%.

The ASCs were characterized using various surface markers. For adipogenic and osteogenic differentiation, oil red O and alizarin red staining, respectively, were performed as described previously. The third passage ASCs were used in the subsequent experiments.

### Adipogenic and osteogenic differentiation

For the adipogenesis experiments, post-confluent human ASCs were cultured with adipogenic induction medium (AIM). DMEM with 10% FBS, 1% penicillin/streptomycin, 10 µg/ml insulin (Sigma, Saint Louis, MO, USA), 10 µM indomethacin (Sigma), 100 nM dexamethasone (Sigma), and 0.5 mM 3-isobutyl methyl-xanthine (Sigma). The medium was changed every 3 days.

For the osteogenesis experiments, we cultured ASCs with osteogenic induction medium (OIM). DMEM with 10% FBS, 1% penicillin/streptomycin, 100 nM dexamethasone, 200 μM ascorbic acid (Sigma), and 10 mM glycerol 2-phosphate (Sigma). The medium was changed every 3 days.

### Oil red O staining and Alizarin red staining

Oil red O staining was performed according to the manufacturer’s protocol for the oil red O staining kit (Cyagen; Suzhou; China). Cells cultured in a 6-well-plate were rinsed with phosphate-buffered saline (PBS), fixed with 4% paraformaldehyde (Tiengen Biological, China) for 10 min, and washed with 60% isopropyl alcohol (Tiengen Biological). Cells were then incubated in 2% (w/v) oil red O staining solution (Cyagen) for 1 h at room temperature. Excess staining was removed by washing with 60% isopropyl alcohol, followed by several washes with distilled water. Lastly, images were collected with an Olympus microscope (BX51, Tokyo; Japan).

Alizarin red staining was performed according to the manufacturer’s protocol for the alizarin red staining kit (Cyagen). Cells cultured in a 6-well-plate were rinsed three times with PBS, fixed with 4% formaldehyde for 10 min at room temperature, and incubated with fresh alizarin red staining solution for 5 min, and then, images were collected with an Olympus microscope (BX51).

### Preparation of the single-cell suspension

The third passage ASCs, cultured in GM, were used to prepare the single-cell suspension of the ASC-GM group. At 70%-80% confluence, the cells were digested with 0.25% trypsin-EDTA (Thermo Fisher Scientific, Waltham, MA, USA). Cells treated with AIM for 1 week (ASC-AD group) were digested with 0.25% trypsin-EDTA. Subsequently, the cells in both groups were centrifuged (1000 rpm; 5 min), and the pellets were resuspended in PBS with 0.04% BSA. The cell concentration was determined by Countstar (IC1000, Aber Instruments Ltd, Ceredigion, UK). The final concentration of the single cells was 0.8–1.2 × 10^6^ cells/mL, and trypan blue (Sigma) staining was used to detect cell viability. If the live cells were more than 80%, and the proportion of single cells was more than 90%, the single-cell suspension was considered qualified, and the next experiment could be carried out.

### Single-cell RNA-seq library preparation and sequencing

The 10X Genomics Chromium platform (10X genomics, Pleasanton, California, USA) was used to capture and barcode the cells to generate single-cell Gel Beads-in-Emulsion (GEMs) by following the manufacturer’s protocol. Chromium Single Cell 3' V2 Chemistry Library Kit, Gel Bead & Multiplex Kit, and Chip Kit from 10X Genomics (Pleasanton, California, USA) were used as a standard procedure of the 3-terminal single-cell transcription sequencing scheme. After cDNA amplification and Library construction, Qubit and Agilent 2100 Bioanalyzer (Agilent Technologies, Palo Alto, CA, USA) were used to measure the concentration. After the database was built, Illumina HiSeq X10 (Illumina, San Diego, CA, USA) was used for sequencing.

### Quality control analysis and cell selection

**Fig. 1:**
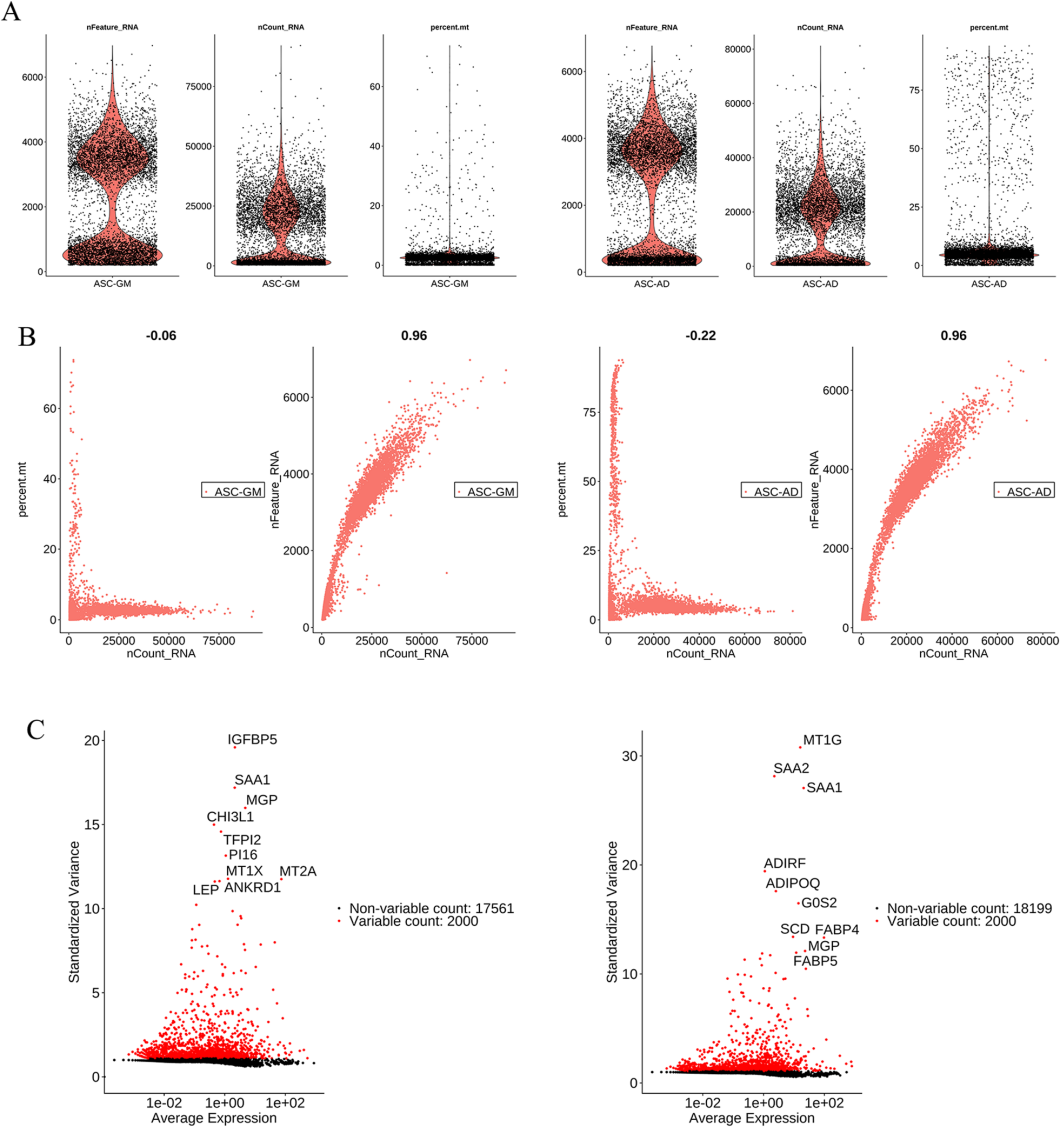
Single-cell RNA-seq data quality assessment and comparison between the AD and GM group of ASCs. **A** The proportion of specific RNA, RNA expression, and mitochondrial gene expression in the samples of the GM and AD groups. **B** The relationships among specific RNA, RNA expression, and mitochondrial gene expression in the samples of the GM and AD groups. **C** The expression distribution of the top 20 principal components in 100 different cell types in the samples of the GM and AD groups

We used the R language Seurat package (cellranger, 10X genomics) to read and manipulate the raw 10X data. Genes expressed in ≥ 3 cells and cells that can detect ≥ 200 genes were retained. We obtained 19,561 genes and 6752 cells in the ASC-GM group (ASCs were cultured in growth medium), and 18,199 genes and 7844 cells in the ASC-AD group (ASCs were cultured in adipogenic inducing medium for 1 week). Next, we plotted the distribution of specific RNA (nFeature_RNA), RNA expression (nCount_RNA), and mitochondrial gene expression (percent. Mt) of the samples and their relationships (Fig. 1 and Additional file 2: Figure S1). Next, we selected the cells between 500 and 6000 expressed genes, a proportion of mitochondrial gene expression not exceeding 0.2, and a sequencing reading not less than 2000. Sequencing reading of expressed genes lesser than 500 were identified as cell fragments and larger than 6000 are considered double or multicellular. Proportion of mitochondria larger than 0.2 and a sequencing reading larger than 2000 are known as programmed death. Lastly, the FindIntegrationAnchors function was implemented to determine the collections and use them to integrate the two data sets into the IntegrateData.

### Clustering analysis

We used the globally standardized LogNormalize method, which adjusted the total expression of each cell to 10,000 (by multiplying with a coefficient S0 and obtaining the logarithm of this value). The ScaleData function was used to remove the gene expression fluctuation caused by the different total expression quantity of cells and different proportions of mitochondrial gene expression. We used the RunPCA function to determine the principal component parameter as npcs = 30. RunUMAP algorithm was then implemented to determine the distribution of the two samples in the UMAP graph. Finally, the FindClusters function was used to conduct a clustering analysis based on the top 30 principal components (clustering precision = 0.5).

The UMAP algorithm was used to display the clustering results, which showed that the integrated sample cells could be divided into subgroups, and the number of cells distributed in each subgroup was displayed. We also plotted the distribution of subgroups of the two samples in the integrated data clustering and the clustering of each subgroup of the integrated data in the two samples. We used the FindAllMarkers function to evaluate the marker genes in various groups. The filter parameters are as follows: only.pos= TRUE, min.pct = 0.25, threx. use = 0.25. That is, only positively labeled genes were screened, and labeled genes were expressed in at least 25% of the cells, with a threshold of 0.25 for differential expression. As shown in Table 1 and Additional file 1: Table S1, the number of tag genes varies among different cell types.

**Table 1:** Top 20 clusters with representative enriched terms (one per cluster)

GO	Category	Description	Count	%	Log10(P)	Log10(q)
GO:0050727	GO Biological Processes	Regulation of inflammatory response	10	22.73	− 7.69	− 3.37
GO:0010757	GO Biological Processes	Negative regulation of plasminogen activation	3	6.82	− 6.95	− 2.93
GO:0019216	GO Biological Processes	Regulation of lipid metabolic process	8	18.18	− 6.12	− 2.42
R-HSA-5661231	Reactome Gene Sets	Metallothioneins bind metals	3	6.82	− 6.04	− 2.42
GO:0071407	GO Biological Processes	Cellular response to organic cyclic compound	8	18.18	− 5.26	− 2.27
GO:0001817	GO Biological Processes	Regulation of cytokine production	9	20.45	− 5.14	− 2.20
GO:0015909	GO Biological Processes	Long-chain fatty acid transport	4	9.09	− 5.12	− 2.20
M167	Canonical Pathways	PID AP1 PATHWAY	4	9.09	− 5.09	− 2.19
GO:0034250	GO Biological Processes	Positive regulation of cellular amide metabolic process	5	11.36	− 4.98	− 2.14
R-HSA-8953897	Reactome Gene Sets	Cellular responses to external stimuli	8	18.18	− 4.96	− 2.14
GO:2000379	GO Biological Processes	Positive regulation of reactive oxygen species metabolic process	4	9.09	− 4.43	− 1.83
GO:0051051	GO Biological Processes	Negative regulation of transport	7	15.91	− 4.28	− 1.79
M5884	Canonical Pathways	NABA CORE MATRISOME	5	11.36	− 3.86	− 1.57
hsa04145	KEGG Pathway	Phagosome	4	9.09	− 3.75	− 1.50
GO:0030279	GO Biological Processes	Negative regulation of ossification	3	6.82	− 3.31	− 1.23
GO:0034109	GO Biological Processes	Homotypic cell–cell adhesion	3	6.82	− 3.31	− 1.23
GO:0031348	GO Biological Processes	Negative regulation of defense response	4	9.09	− 2.95	− 0.96
GO:0007098	GO Biological Processes	Centrosome cycle	3	6.82	− 2.81	− 0.86
GO:0002526	GO Biological Processes	Acute inflammatory response	3	6.82	− 2.11	− 0.30

“Count” is the number of genes in the user-provided lists with membership in the given ontology term. “%” is the percentage of all of the user-provided genes that are found in the given ontology term (only input genes with at least one ontology term annotation are included in the calculation). “Log10(P)” is the *p* value in log base 10. “Log10(q)” is the multi-test adjusted *p* value in log base 10

### Different subsets of cell identity annotations

We used the intersection of the label genes of each type of cell in this sample and the label genes of 467 known cells in the CellMarker database for the Fisher’s exact test. The analysis results are shown in Additional file 1: Table S2 and S3. We sorted by *p* value and selected the top 1 known name of cells to annotate the current subgroup of cells.

### Selection and analysis of hub genes

Cytoscape (https://cytoscape.org/) was used to identify 46 genes as hub genes. The gene identifiers were first converted into their corresponding H. sapiens Entrez gene IDs using the latest version of the database (last updated on 2020-03-19). If multiple identifiers correspond to the same Entrez gene ID, they will be considered as a single Entrez gene ID in downstream analyses. There are 44 unique genes in input ID. The following are the list of annotations retrieved from the latest version of the database (last updated on 2020-03-19) (Additional file 1: Table S4).

### Pathway and process enrichment analysis

For each given gene list, pathway, and process enrichment, an analysis has been carried out by String (https://string-db.org/) with the following ontology sources: KEGG Pathway, GO Biological Processes, Reactome Gene Sets, Canonical Pathways, and CORUM. All genes in the genome were used as an enrichment background. Terms with a *p* value < 0.01, a minimum count of 3, and an enrichment factor > 1.5 (the enrichment factor is the ratio between the observed counts and the counts expected by chance) are collected and grouped into clusters based on their membership similarities. More specifically, *p* values are calculated based on the accumulative hypergeometric distribution, and q-values are calculated using the Benjamini–Hochberg procedure, which accounts for multiple testing. Kappa scores are used as the similarity metric when performing hierarchical clustering on the enriched terms, and sub-trees with a similarity > 0.3 are considered a cluster. The most statistically significant term within a cluster was chosen to represent the whole cluster.

To further capture the relationships between the terms, a subset of enriched terms has been selected and rendered as a network plot, where edges connect terms with a similarity > 0.3. We select the terms with the best *p* values from each of the 20 clusters, with the constraint that there are no more than 15 terms per cluster and no more than 250 terms in total. The network is visualized using Cytoscape, where each node represents an enriched term and is colored first by its cluster ID and then by its *p* value.

For each given gene list, protein–protein interaction enrichment analysis has been carried out with the following databases: BioGrid, InWeb_IM, and OmniPath. The resultant network contains the subset of proteins that form physical interactions with at least one other member in the list. If the network contained between 3 and 500 proteins, the Molecular Complex Detection (MCODE) algorithm was applied to identify densely connected network components. The MCODE networks identified for individual gene lists were gathered.

### Western blot analysis

Cells were collected in a pH 8.0 lysis buffer (50 mM Tris HCl, 120 mM NaCl, and 0.5% NP-40; Keygen Biotech, Nanjing, China). Protein concentrations in the extracts were estimated using the Bradford method. A total of 20 μg of protein sample was loaded per lane, separated by polyacrylamide gel electrophoresis, and then transferred to polyvinylidene fluoride (PVDF) membranes (Millipore; Waltham, MA, USA). Membranes were subsequently blocked in 5% nonfat milk (BD Biosciences, Franklin Lakes, NJ, USA) in Tris Buffered Saline with 0.05% Tween 20 (TBST) for 1 h at room temperature and incubated with anti-SAA1+SAA2 antibody (dilution 1:500, ab207445; Abcam; Cambridge, UK), adiponectin antibody (1:500, Abcam), CEBPα antibody (1:500, 2295s, Cell Signaling Technology; Danvers, MA, USA), or anti-glyceraldehyde 3-phosphate dehydrogenase (GAPDH) antibody (1:5000; AP0063; Bioworld Company, Bloomington, MN, USA) primary antibodies at 4 °C overnight. The membranes were washed for 15 min in TBST and then incubated for 1 h with horseradish peroxidase (HRP)-conjugated secondary antibodies (dilution 1:5000; Fudebio, Hangzhou, China) in TBST at room temperature. After washes with TBST, the membranes were visualized by enhanced chemiluminescence (ECL) detection reagents (Fudebio). Immunoreactive bands were detected by the ECL detection system (Protein Simple, Silicon Valley, CA, USA). The immunoreactive bands were quantification analyzed with ImageJ software (V1.4.3.64; National Institutes of Health; Bethesda, MD, USA), and the relative expression of each protein was normalized to GAPDH.

### Immunofluorescence analysis

Cells were fixed with 4% paraformaldehyde for 10 min, and membranes were ruptured by 0.1% Triton-X 100 (Sigma) for 5 min. The samples were blocked with 2% BSA (diluted with PBS) for 1 h at room temperature, then incubated with α-tubulin antibody (dilution 1:500; ab7291; Abcam), 4 °C overnight. Incubation with the secondary antibody was performed at room temperature for 1 h the next day. Nuclei were labeled with DAPI. Samples were sealed with glycerin gelatin. Images were collected using a confocal microscope (LSM 880 with Airyscan; Carl Zeiss; Oberkochen, Germany) and analyzed with software ZEN-blue-edition (Carl Zeiss; Germany).

### Transfection

Lentivirus vectors repressing SAA1 (AGGGTACACAATGGGTATCTA) were constructed and generated by Genechem (Additional file 2: Figure S1, Shanghai; China). In preliminary experiment, we selected the optimal lentiviral infection settings, such as number of cells to inoculate, infection volume, and treatment time. The cells were seeded at 5000 cells/cm^2^ in 6-well-plate. Medium was changed to GM with polybrene when the cell confluence got 60%, and the lentivirus was then added in. After 12 h, the medium was replaced with GM. At 72 h, the expression of green fluorescent protein was observed by fluorescence microscopy and the transfection efficiency was evaluated. Additionally, siControl cells were transfected with a negative control virus (TTCTCCGAACGTGTCACGT) which were constructed by Genechem.

### Statistical analyses

All experiments were independently repeated at least three times. Statistical analysis was performed with GraphPad Prism 8.0 software (GraphPad Prism, La Jolla, California, USA). The student’s t test was used to identify significant differences, and *p* < 0.05 indicated statistical significance.

## Results

### Quality control analysis and cell selection

We obtained 19,561 genes and 6752 cells in the ASC-GM group that the ASCs were cultured in growth medium, and 20,199 genes and 7844 cells in the ASC-AD group that the ASCs were cultured in adipogenic inducing medium for 1 week. Next, we plotted the distribution of specific RNA (nFeature_RNA), RNA expression (nCount_RNA), and mitochondrial gene expression (percent. Mt) of the samples and their relationships (Fig. 1A, B, and Additional file 3: Figure S2). We selected cells with gene expression quantity between 500 and 6000, mitochondrial gene expression proportion not more than 0.2, and sequencing reading not less than 2000. We obtained 4415 cells in the ASC-GM group and 4634 cells in the ASC-AD group.

### Clustering analysis

**Fig. 2:**
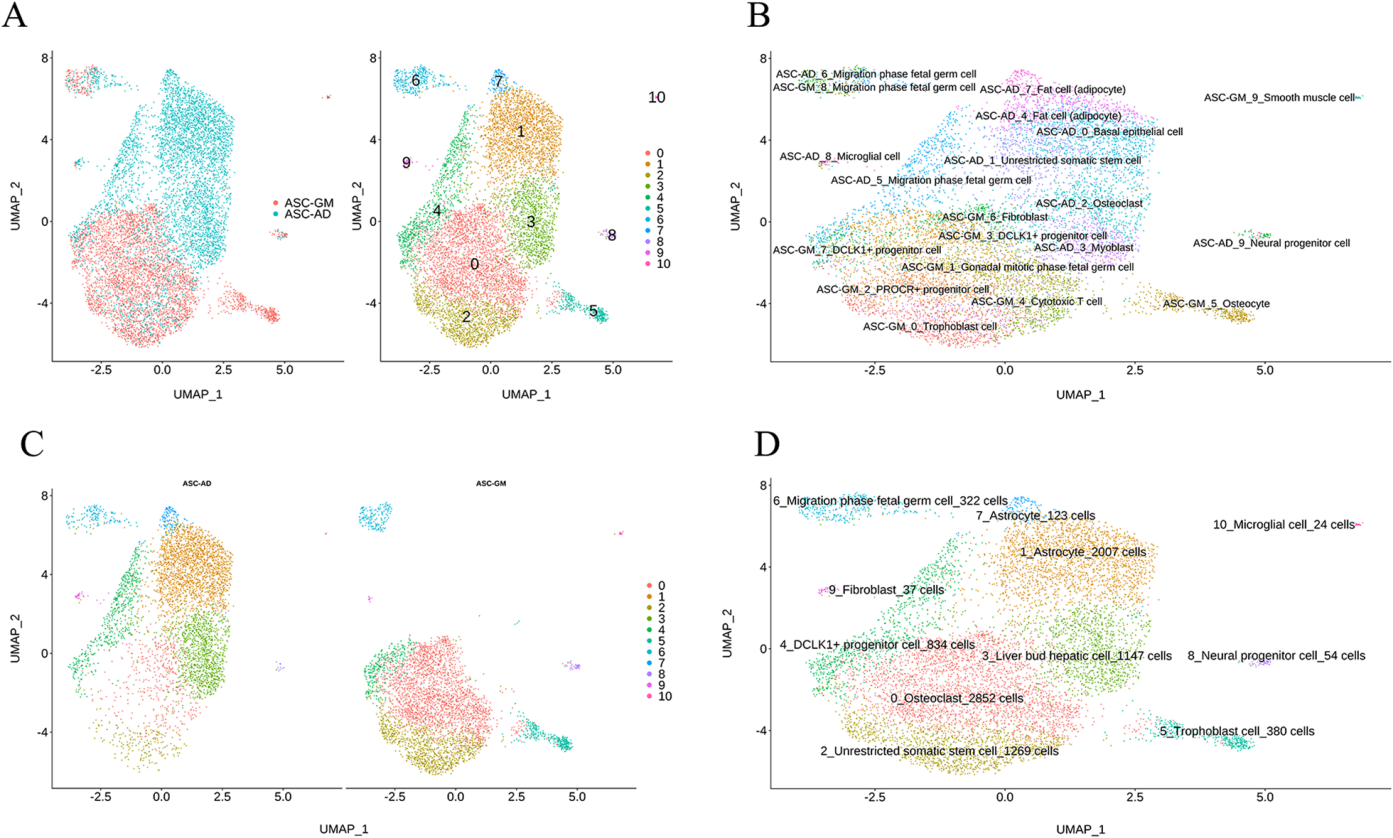
**A** UMAP clustering results of the top 30 principal components and sample distribution. **B** The distribution of subpopulations of two samples in the integrated data UMAP clustering results. **C** The UMAP clustering of each subgroup of the integrated data in the two samples. **D** The number of cells distributed in each subgroup in the integrated data UMAP cluster

The integrated sample cells could be divided into 11 subgroups (0–10 cluster; Fig. 2A, B), and the number of cells distributed in each subgroup was displayed. Because the cluster 8–10 had only 115 cells (1.27% in total cells), we only focus on the subgroups 0–7. We also plotted the distribution of subgroups of the two samples in the integrated data clustering (Fig. 2B) and the clustering of each subgroup of the integrated data in the two samples (Fig. 2C, D). The cells in cluster 0, 2, 5 were came from ASC-GM group and the cells in cluster 1, 3, 7 came from ASC-AD group. The cells of cluster 4 and 6 came from both ASC-GM and ASC-AD groups.

### Screening of marker genes for each subgroup

As shown in Fig. 1C, the number of tag genes varied among different subgroups of cells. Additionally, the cell proportions of the two samples also varied among different subgroups. These results are summarized in Additional file 1: Tables S1 and S2.

**Fig. 3:**
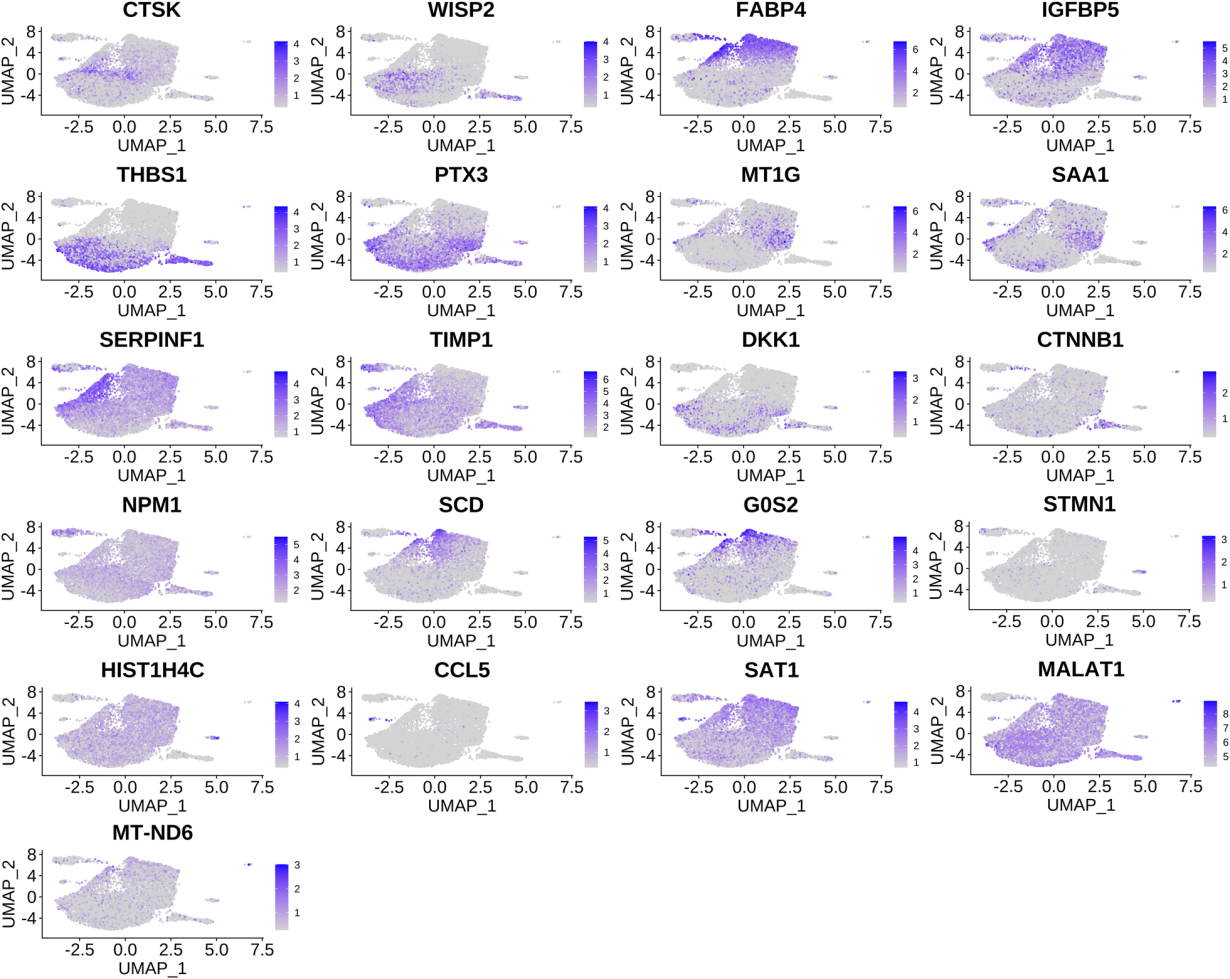
Distribution map of the top 2 tag genes expressed in different subgroups. The color depth represents the intensity of expression in the samples

**Fig. 4:**
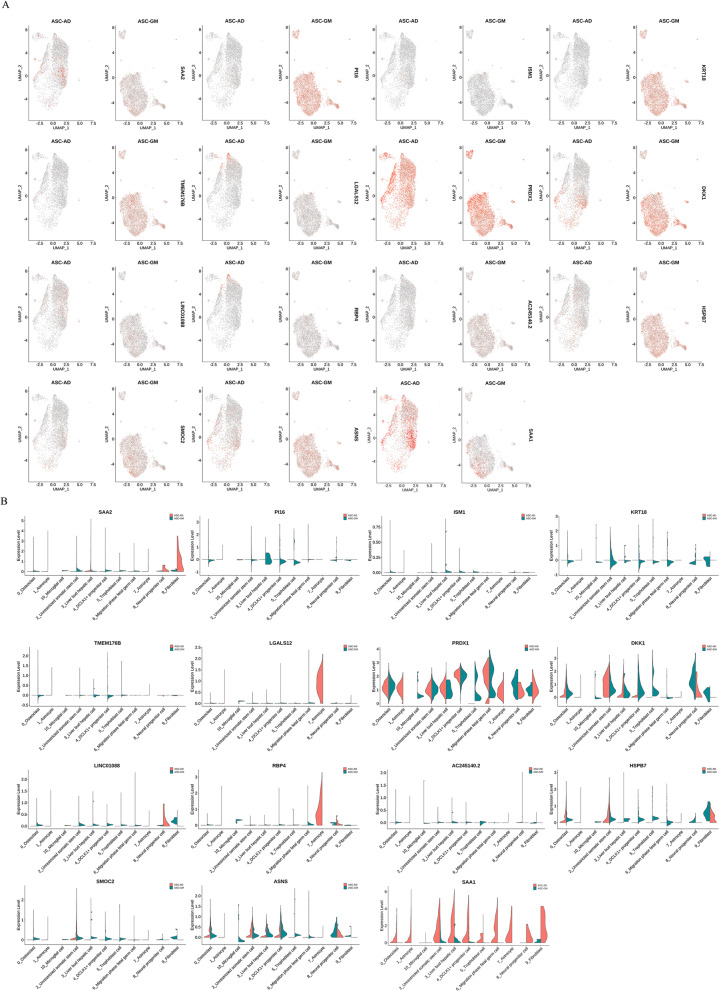
Expression distribution map and violin diagram of the top 2 differentially expressed genes in each subgroup. **A** An expression distribution map. **B** Violin diagram

We plotted the expression profiles of the top 2 tag genes (ordered by ratio) in different subgroups (Fig. 3) and the bubble plots (Additional file 4: Figure S3) of the top 5 tag genes. There were 21 genes include fatty acid binding protein 4 (FABP4), serum amyloid A1 (SAA1), C-C Motif Chemokine Ligand 5 (CCL5) shown in Fig. 3. FABP4 was positively expressed in some of the cells, and FABP4 is a widely recognized adipocyte marker, which fully demonstrates the successful adipogenic differentiation of some cells and the heterogeneity of ASCs differentiation. And then, the top 5 tag genes were sorted by *p* value, and we selected the most known name of cells to annotate the current category of cells (see Additional file 1: Table S2). The analysis results are shown in Additional file 1: Table S2 and S3. We selected the top 2 differentially expressed genes from each subgroup for the expression distribution map (Fig. 4A) and the violin plot (Fig. 4B). There were 14 genes including serum amyloid A2 (SAA2), shown in Fig. 4.

### Annotation for different subsets of cells

**Fig. 5:**
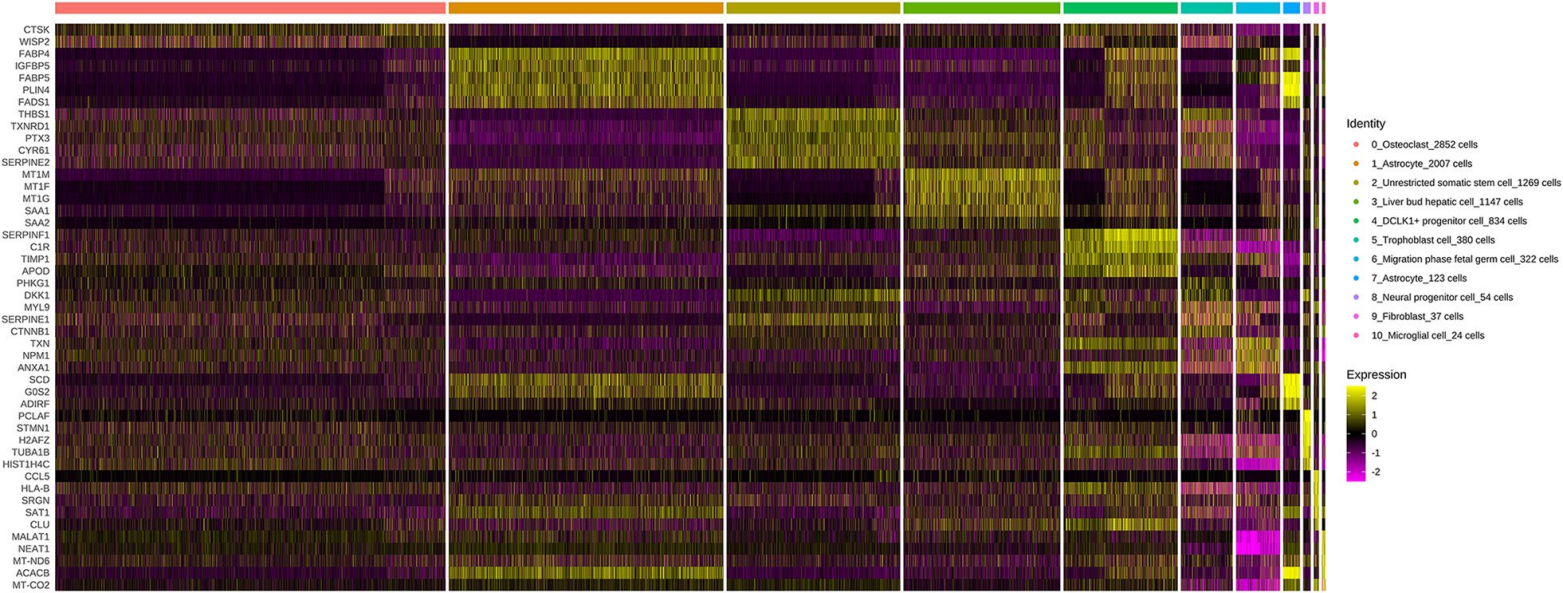
Heatmap of the top 5 tag genes expressed in different subgroups. Each row represents a gene, and each column represents a single cell, and the color depth represents the intensity of expression in the samples of the GM and AD groups

We classified the cells into 11 subgroups; the annotation of these subgroups was identified in Additional file 1: Table S5. There were huge differences in cell number and mark genes between category 1 and category 7, although both were named as “Astroctyte” (Fig. 5 and Additional file 3: Figure S2). The conventional markers of adipocytes, FABP4, fatty acid binding protein 5 (FABP5), complement factor D (CFD), or usually highly expression genes in adipose tissue, fatty acid desaturase 1 (FADS1), and insulin like growth factor binding protein 5 (IGFBP5) were high expressed in category 1, 7 and low expressed in category 0, 2, 3, 5. It showed that most cells of category 1, 7 came from ASC-AD group, while most cells of category 0, 2, 5 came from ASC-GM group except category 3 that came from ASC-AD group. These results also indicated that our adipogenic induction was successful on the one hand, and on the other hand, there was great heterogeneity in the adipogenic differentiation of ASCs. Except for category 10, the expression of SAA1 in ASC-AD group of the other 10 categories was significantly increased compared with ASC-GM group (Fig. 4).

### Pathway and process enrichment analysis

**Fig. 6:**
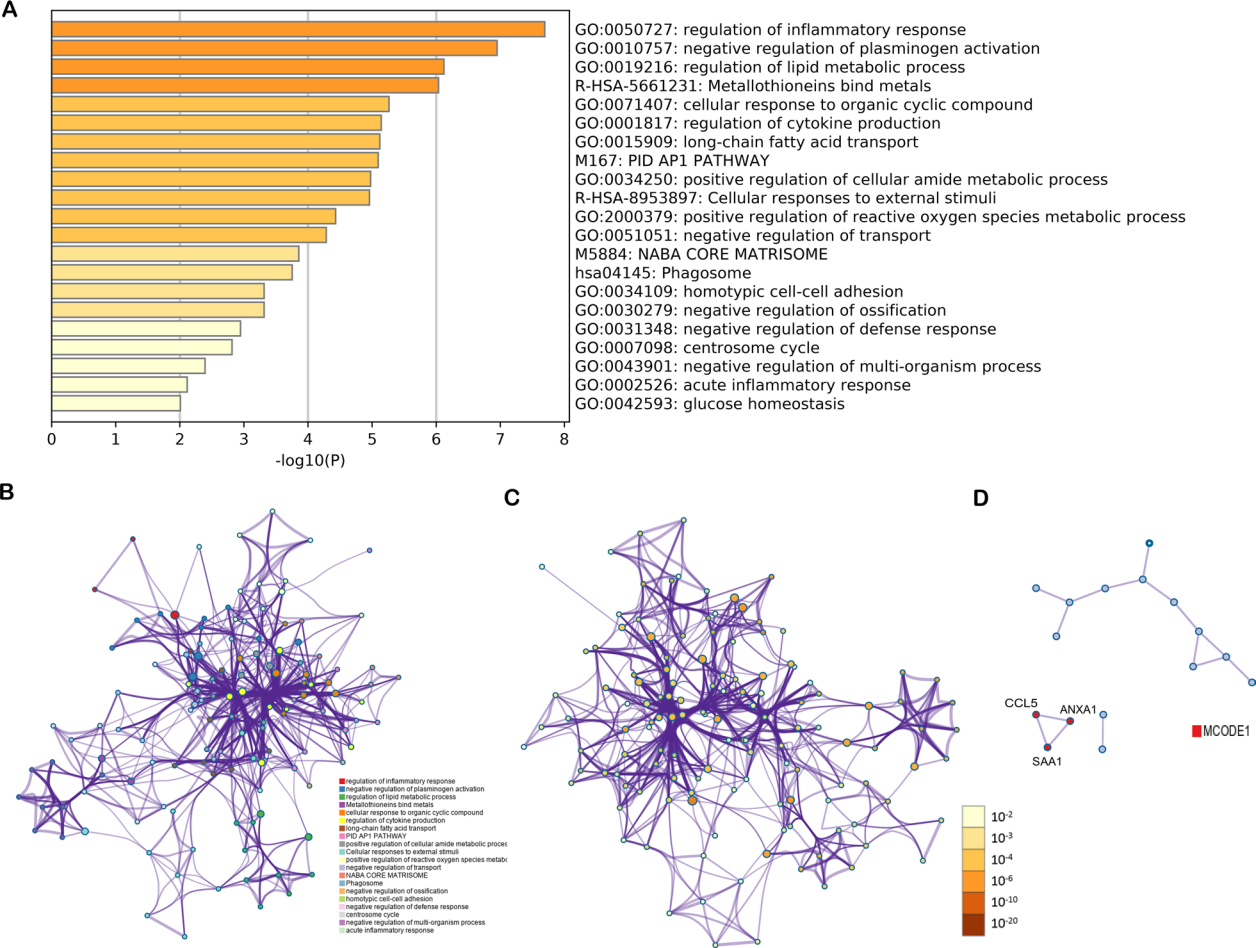
Pathway and process enrichment analysis. **A** Bar graph of enriched terms across input gene lists, colored by *p* values. The network of enriched terms: **B** colored by cluster ID, where nodes that share the same cluster ID are typically close to each other. Each term is represented by a circle node, where its size is proportional to the number of input genes fall into that term, and its color represents its cluster identity (i.e., nodes of the same color belong to the same cluster). Terms with a similarity score > 0.3 are linked by an edge (the thickness of the edge represents the similarity score). The network is visualized with Cytoscape with “force-directed” layout and with edge bundled for clarity. One term from each cluster is selected to have its term description shown as label. **C** The same enrichment network has its nodes colored by *p* value, as shown in the legend. The dark the color, the more statistically significant the node is (see legend for *p* value ranges). **D** protein–protein interaction network and MCODE components identified in the gene lists. MCODE algorithm was then applied to this network to identify neighborhoods where proteins are densely connected. Each MCODE network is assigned a unique color. GO enrichment analysis was applied to each MCODE network to assign “meanings” to the network component

Regulation of inflammatory response is the rank 1 biological processes. And cellular responses to external stimuli, negative regulation of defense response and acute inflammatory response are included in top 20 biological processes (Fig. 6A). These four biological processes are all related to the response of cells to external stimuli, such as acute inflammatory response.

The network of enriched terms was shown in cluster ID (Fig. 6B) with *p* value (Fig. 6C). Based on the MCODE results, we found that SAA1, C-C Motif Chemokine Ligand 5 (CCL5), and Annexin A1 (ANXA1) significantly highly expressed during adipogenic differentiation (Fig. 6D).

### The role of SAA1 adipogenesis of ASCs

A considerable number of reports has been published on the numerous biological activities attributed to SAA1, most of which state that it is pro-inflammatory. SAA1 has been shown to upregulate the expression of various inflammatory mediators such as cell adhesion molecules, cytokines, chemokines, matrix-degrading proteases, reactive oxygen species (ROS), and pro-angiogenic molecules in several cell types including leukocytes, fibroblasts, and endothelial cells [17–22].

**Fig. 7:**
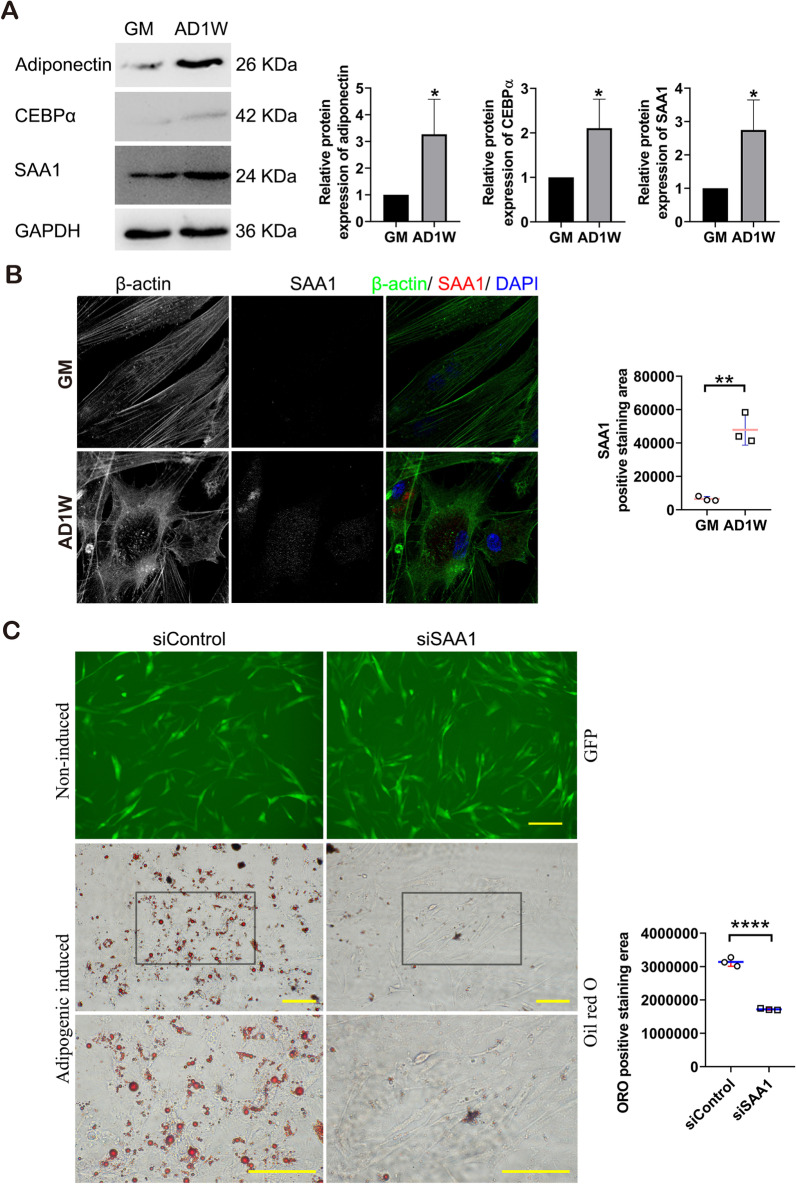
SAA1 changed during the adipogenic differentiation of human ASCs. **A** Protein expression analysis of SAA1 and adipogenic differentiation protein markers after adipogenic-induced one week. GAPDH was used as the internal reference gene. Mean ± SD, *p < 0.05, *n* = 3. GM: human ASCs were cultured in growth medium; AD1W: human ASCs were cultured in adipogenic inducing medium 1 week. **B** Morphological changes of SAA1 during the adipogenic differentiation of ASCs. SAA1 is marked in red, microfilaments are marked with phalloidin in green, and the nucleus is marked with DAPI in blue. Scale bar = 50 µm. Mean ± SD, ** P < 0.01, *n* = 3. **C** Oil red O staining for triglyceride content and quantitative analysis in SAA1-silenced ASCs (siSAA1) after 4 days of adipogenic differentiation. Negative control group was siControl group. Scale bar = 100 μm. Mean ± SD, ****p < 0.0001, *n* = 3

The role of SAA1 during the adipogenic differentiation of ASCs was verified using immunofluorescence and western blot experiments. ASCs have been adipogenic-induced successful based on the western blot results of adiponectin and CEBPα. Western blot experiment results showed that the protein expression of SAA1 increased during adipogenesis (Fig. 7A). The SAA1-positive staining area of ASCs treated with adipogenic induction medium (for 1 week) was significantly higher than that of untreated ASCs (Fig. 7B). To investigate how SAA1 contributed to adipogenesis, we performed oil red O (ORO) staining after 4 days of adipogenic differentiation in response to SAA1 knockdown. The area of ORO positive staining in the negative control (siControl) cells was significantly higher than that in the siSAA1 cells after 1 week of treatment with adipogenic induction medium (Fig. 7C).

## Discussion

ASCs are distributed in the region surrounding the vascular system of adipose tissue. The content of ASCs in fat aspirates obtained by liposuction is approximately 2% [23]. ASCs, which have numerous sources and vast reserves in vivo, are suitable for autologous transplantation and adipose tissue engineering [24–27]. Understanding the mechanisms involved in the regulation of adipose tissue development and the differentiation of ASCs is crucial. It will promote our understanding of the impact of fat generated under physiological conditions and help in the development of potential new treatments for obesity prevention as well as clinically relevant products for soft tissue repair. Burl et al. [28] employed single-cell RNA sequencing (scRNA-seq) of mouse adipose tissue to identify distinct subpopulations of adipocyte progenitors and immune cells. And Rondini and Granneman [29] commented these researches. And our results showed a heterogeneity of gene expression in adipogenically induced cells of human adipose-derived stem cells and that the regulation of inflammatory response and the expression of serum amyloid A1 is upregulated and required during adipogenic differentiation.

The development of single-cell sequencing and bioinformatics strategies is progressing continuously, and many achievements have been made in recent years. Single-cell sequencing is valuable for the study of the heterogeneity of highly complex cell populations and in the analysis of their cell subpopulations as well as changes in their molecular mechanisms [30–33]. The main procedure involves establishing a library of multiple single-cells independent from each other with their message, through amplification of nucleic acid information, followed by second-generation sequencing and bioinformatic analysis. The single cells could be obtained by a flow cell sorting or microfluidic control system or other systems [6, 34]. Additionally, a variety of code-labeling, decoding, information amplification, fidelity processes or namely deviation correction, sequencing data digitization and standardization, cell clustering and cluster identification, pathway, and other molecular mechanism analyses, need to be applied [35]. We used single-cell RNA sequencing and bioinformation to do analyzing. Although the adipose-derived stem cells were considered homogenous cells, the scRNA-seq analysis here revealed the nuance difference among cells in both ASCs and AD cells, with several subpopulations identified each with specific transcriptomic pattern.

Population cell sequencing and analysis can mask the unique role of different cell subsets. This means that some non-dominant subsets in the stem cell population may play a more critical role in differentiation. Single-cell omics analysis provides a unique high-resolution method for the study of complex and heterogeneous cellular systems. Single-cell omics recently is widely used to identify the molecular pathways, regulatory mechanisms, and lineage trajectory analysis for stem cell differentiation [36–38]. Based on this experiment, we found that ASCs are very highly heterogeneous during adipogenic differentiation.

Based on our analysis, it is reasonable to assume that the cocktail inducer we used induces adipogenic differentiation of ASCs, which is a strong stimulus for ASCs. In this study, we identified that SAA1 is an important regulator in the differentiation of ASCs. Significantly high expression of SAA1 is indicative of an acute response, which is mainly produced by the liver. It is a protective response of the human body to environmental stimuli, such as tissue injury and infection. Human SAA1 is encoded by one of the four SAA genes and is the best-categorized SAA protein. Formerly known as a major precursor of amyloid A, SAA1 has been found to play a vital role in lipid metabolism and the regulation of inflammation [39, 40]. We discovered the regulatory role of SAA1 in ASCs differentiation in response to external stimuli.

Li et al. [41, 42] found that SAA1 was highly expressed in the fibroblasts and epithelium of the amnion and trophoblasts of the chorion. SAA1 production was amplified by interleukin-1β and cortisol alone or in combination. Additionally, SAA1 increased the expression of interleukin-1β, interleukin-6, cyclooxygenase-2, and prostaglandin E2 (PGE2). These functions of SAA1 were intermediated through activation of the nuclear transcription factor-κB (NF-κB), p38, and extracellular regulated protein kinases (ERK1/2) signaling pathways by toll-like receptor 4. Wang et al. [43] found that silencing SAA1 could inhibit the progression of obesity-induced insulin resistance through the NF-κB pathway based on ex vivo and mouse experiments. Poitou C et al. found that acute phase SAA played an important role in the crosstalk of adipocyte and macrophage based on microarray analysis [14]. These previous studies are consistent with our research results, which support that adipogenic induction medium is a robust external stimulus for ASCs. We found that the expression of SAA1 is significantly increased during the adipogenic differentiation of ASCs, and we further confirmed this hypothesis by demonstrating the reduction in the adipogenic differentiation efficiency of ASCs after SAA1 silencing.

## Conclusion

Based on single-cell RNA sequencing analysis, we confirmed that ASCs had a high heterogeneity during adipogenic differentiation. Regulation of inflammatory response, cellular responses to external stimuli, negative regulation of defense response, and acute inflammatory response are included in top 20 biological processes. We found that SAA1, CCL5, and ANXA1 significantly highly expressed during adipogenic differentiation, based on the MCODE results. The results of western blot, immunofluorescent staining, and siSAA1 demonstrated that SAA1 and regulation of inflammatory response were involved in adipogenesis of ASCs, especially in early stage of adipogenesis. So, we found that regulation of inflammatory response and SAA1 may play a vital role in the adipogenic differentiation of ASCs. The specific mechanism needs to be further analyzed in future studies. These findings not only contribute to a better understanding of the regulatory mechanism of ASCs adipogenic differentiation, but also provide new ideas and targets for us to precisely regulate ASCs adipogenic differentiation.

## Supplementary Information

**Additional file 1: Table S1.** Differentially expressed genes. **Table S2.** The number of label genes, annotations, and cell numbers of different subgroups in UMAP clustering results. **Table S3.** Combine annotation cluster cells. **Table S4.** Gene annotations extracted. **Table S5.** Statistics of input gene lists.

**Additional file 2: Figure S1.** Structure of SAA1 virus vector: hU6-MCS-Ubiquitin-EGFP-IRES-puromycin.

**Additional file 3: Figure S2.** (A) and (B) show the expression distribution and standard deviation of the top 20 principal components in 100 different types of cells, respectively. (C) and (D) show the expression distribution of the top 20 principal components in 100 different types of cells.

**Additional file 4: Figure S3.** Bubble diagram of the top 5 tag genes expressed in different subgroups.

## Data Availability

The code for processing the data from a combined raw UMI count matrix to a clean gene-cell matrix is available online.

## Abbreviations

ASCs: Adipose-derived stem cells
SAA: Serum amyloid A
SAA1: Serum amyloid A1
scRNA-seq: Single-cell transcriptomic sequencing
MCODE: Molecular Complex Detection
CCL5: C-C Motif Chemokine Ligand 5
ANXA1: Annexin A1
ROS: Reactive oxygen species
ORO: Oil red O
PGE2: Prostaglandin
NF-κB: Nuclear transcription factor-κB
ERK1/2: Extracellular regulated protein kinases
GM: Growth medium
DMEM: Dulbecco’s modified Eagle’s medium
FBS: Fetal bovine serum
AIM: Adipogenic induction medium
OIM: Osteogenic induction medium
PBS: Phosphate-buffered saline
GEMs: Gel Beads-in-Emulsion
PVDF: Polyvinylidene fluoride
TBST: Tris Buffered Saline with Tween 20
GAPDH: Glyceraldehyde 3-phosphate dehydrogenase
ECL: Enhanced Chemiluminescence
FABP4: Fatty acid binding protein 4
FABP5: Fatty acid binding protein 5
CFD: Complement factor D
FADS1: Fatty acid desaturase 1
IGFBP5: Insulin like growth factor binding protein 5
