# Supplementary material for: The role of serum amyloid A1 in the adipogenic differentiation of human adipose-derived stem cells basing on single-cell RNA sequencing analysis
Source: Stem Cell Res Ther. 2022 May 7;13:187. doi: 10.1186/s13287-022-02873-5 (PMC9080218; doi:10.1186/s13287-022-02873-5)
Supplement: Supplementary file 1 — Additional file 1: Table S1. Differentially expressed genes. Table S2. The number of label genes, annotations, and cell numbers of different subgroups in UMAP clustering results. Table S3. Combine annotation cluster cells. Table S4. Gene annotations extracted. Table S5. Statistics of input gene lists. [file 13287_2022_2873_MOESM1_ESM.pdf]

Table S1 Differentially expressed genes

p_val	avg_logFC	pct.1	pct.2	p_val_adj	cluster	gene
6.57E-150	0.327744	0.98	0.787	1.31E-146	0	CTSK
1.24E-26	0.259996	0.582	0.288	2.47E-23	0	WISP2
0	1.230391	0.998	0.987	0	1	FABP4
0	1.21411	0.98	0.939	0	1	IGFBP5
0	0.934944	0.897	0.842	0	1	FABP5
0	0.771898	0.902	0.799	0	1	PLIN4
0	0.731208	0.985	0.946	0	1	ACACB
0	0.701706	0.816	0.76	0	1	AOC3
0	0.665955	0.987	0.958	0	1	CFD
0	0.65633	0.898	0.845	0	1	PNPLA2
0	0.644079	0.915	0.854	0	1	HEBP2
0	0.643581	0.984	0.94	0	1	CIDEC
0	0.56306	0.827	0.802	0	1	RASD1
0	0.487507	0.963	0.941	0	1	SHC1
0	0.451321	0.998	0.994	0	1	DBI
3.47E-292	0.453777	0.962	0.948	6.94E-289	1	ACSL1
2.92E-281	0.545375	0.935	0.892	5.84E-278	1	PLA2G16
3.59E-278	0.464738	0.94	0.912	7.18E-275	1	C1QTNF1
6.35E-272	0.444825	0.925	0.885	1.27E-268	1	COL4A1
1.45E-269	0.523249	0.835	0.833	2.91E-266	1	FASN
2.38E-260	0.465673	0.885	0.888	4.76E-257	1	APCDD1
1.16E-256	0.39774	0.936	0.93	2.32E-253	1	DHCR24
5.36E-251	0.359907	0.907	0.894	1.07E-247	1	SLC25A1
3.49E-249	0.398092	0.979	0.936	6.97E-246	1	LAMA4
2.17E-241	0.408502	0.994	0.988	4.35E-238	1	CYB5A
2.59E-241	0.476942	0.995	0.988	5.19E-238	1	SAT1
1.56E-233	0.384379	0.957	0.936	3.12E-230	1	COL4A2
1.27E-228	0.674396	0.887	0.896	2.54E-225	1	SCD

1.36E-227	0.759571	0.729	0.732	2.72E-224	1	PLIN1
2.85E-225	0.445057	0.984	0.97	5.71E-222	1	ADH1B
4.84E-220	0.295895	0.999	0.989	9.67E-217	1	SRPX
1.17E-210	0.335341	0.783	0.813	2.35E-207	1	TSPAN14
1.31E-203	0.42244	0.752	0.736	2.63E-200	1	ERV3-1
1.59E-199	0.3012	0.985	0.958	3.19E-196	1	AKR1C1
5.08E-194	0.344425	0.971	0.959	1.02E-190	1	ELOVL5
5.56E-190	0.774599	0.873	0.899	1.11E-186	1	FADS1
3.67E-183	0.366744	0.761	0.801	7.35E-180	1	SORT1
2.97E-182	0.351036	0.82	0.862	5.94E-179	1	FZD4
1.20E-176	0.346087	0.768	0.81	2.40E-173	1	AKR1C2
3.53E-176	0.543557	0.698	0.725	7.06E-173	1	RDH5
1.20E-175	0.478362	0.838	0.869	2.41E-172	1	INSIG1
1.63E-162	0.278349	0.822	0.693	3.27E-159	1	ADAM12
6.11E-160	0.721553	0.83	0.854	1.22E-156	1	G0S2
6.37E-152	0.277694	0.998	0.995	1.27E-148	1	PRDX6
2.03E-139	0.329332	0.744	0.811	4.05E-136	1	VKORC1L1
5.68E-136	0.288065	0.994	0.98	1.14E-132	1	HSPB6
5.27E-135	0.437267	0.702	0.758	1.05E-131	1	PDK4
1.19E-126	0.32111	0.724	0.763	2.37E-123	1	RHOB
3.06E-120	0.429149	0.646	0.71	6.12E-117	1	AQP7
1.32E-104	0.252269	0.939	0.877	2.65E-101	1	SH3PXD2A

6.26E-102	0.269719	0.772	0.655	1.25E-98	1	MYLK
4.45E-99	0.389038	0.988	0.988	8.91E-96	1	GLUL
9.81E-97	0.351295	0.751	0.848	1.96E-93	1	GPAM
4.19E-95	0.452513	0.583	0.623	8.38E-92	1	UCP2
3.74E-93	0.309551	0.947	0.944	7.48E-90	1	GSN
5.36E-91	0.259363	0.655	0.742	1.07E-87	1	CSAD
1.08E-79	0.2603	0.786	0.854	2.15E-76	1	ACLY
2.05E-79	0.268582	0.715	0.829	4.09E-76	1	LPIN1
6.85E-68	0.295967	0.733	0.82	1.37E-64	1	AKAP12
5.98E-67	0.291859	0.64	0.673	1.20E-63	1	ALDH1A3
6.44E-67	0.314592	0.605	0.74	1.29E-63	1	MLXIPL
2.76E-65	0.74405	0.603	0.738	5.52E-62	1	LPL
1.07E-52	0.358597	0.679	0.82	2.13E-49	1	IGF1
8.06E-43	0.304973	0.527	0.617	1.61E-39	1	ITIH5
6.41E-42	0.267973	0.759	0.877	1.28E-38	1	FADS2
6.80E-41	0.461252	0.55	0.699	1.36E-37	1	GPD1
4.72E-30	0.33402	0.292	0.647	9.44E-27	1	FABP3
4.25E-29	0.304891	0.568	0.737	8.49E-26	1	CPB1
2.84E-27	0.303706	0.521	0.689	5.67E-24	1	ITGA7
1.53E-20	0.32584	0.532	0.722	3.07E-17	1	FAM213A
3.87E-19	0.260496	0.53	0.731	7.75E-16	1	RAMP2
7.58E-19	0.286687	0.561	0.772	1.52E-15	1	HMGCS1
3.58E-18	0.266724	0.335	0.671	7.17E-15	1	MRAP
2.62E-10	0.25387	0.48	0.687	5.23E-07	1	HK2
0	0.775772	0.944	0.523	0	2	THBS1
0	0.404678	0.979	0.665	0	2	ALCAM
5.11E-304	0.540833	0.985	0.711	1.02E-300	2	FBN2
2.54E-255	0.469728	0.97	0.533	5.09E-252	2	DKK1
8.84E-252	0.536473	0.969	0.653	1.77E-248	2	HEG1
7.83E-248	0.547361	0.996	0.81	1.57E-244	2	TXNRD1
4.93E-246	0.417617	0.998	0.95	9.87E-243	2	CAVIN1
5.65E-246	0.528874	0.99	0.776	1.13E-242	2	ITGBL1
2.00E-243	0.520703	0.995	0.846	4.00E-240	2	LOX
5.42E-242	0.77653	0.998	0.813	1.08E-238	2	PTX3
1.83E-238	0.468817	0.976	0.67	3.66E-235	2	CYTOR

6.57E-235	0.39892	0.981	0.654	1.31E-231	2	LINC01133
6.39E-232	0.314817	0.974	0.661	1.28E-228	2	PSAT1
5.76E-226	0.600487	0.924	0.552	1.15E-222	2	CYR61
2.63E-214	0.516055	1	0.979	5.26E-211	2	FN1
9.31E-210	0.752308	0.935	0.596	1.86E-206	2	SERPINE2
1.37E-208	0.26603	0.965	0.684	2.75E-205	2	ID1
2.52E-208	0.494839	0.986	0.747	5.03E-205	2	USP53
1.97E-181	0.303087	0.916	0.523	3.93E-178	2	TMEM47
7.25E-180	0.284145	1	1	1.45E-176	2	TMSB10
1.17E-177	0.345467	0.995	0.818	2.35E-174	2	PLEC
8.22E-169	0.337152	0.997	0.901	1.64E-165	2	CLIC4
1.06E-160	0.310395	0.989	0.793	2.13E-157	2	NABP1
3.72E-156	0.401607	0.999	0.989	7.45E-153	2	ACTG1
8.76E-154	0.264689	0.801	0.342	1.75E-150	2	SFRP4
4.37E-150	0.365337	0.897	0.518	8.74E-147	2	PTGIS
2.71E-144	0.34191	0.983	0.851	5.42E-141	2	SLC38A2
2.84E-144	0.322844	0.976	0.761	5.68E-141	2	MARCKS
4.54E-142	0.420694	0.822	0.419	9.09E-139	2	SERPINE1
8.67E-142	0.280301	0.834	0.443	1.73E-138	2	DDAH1
5.51E-141	0.321625	0.987	0.831	1.10E-137	2	FLNA
1.42E-137	0.277727	0.736	0.249	2.85E-134	2	STC2
3.14E-137	0.285873	0.835	0.426	6.28E-134	2	CRIM1

8.14E-136	0.331397	0.99	0.819	1.63E-132	2	EMP1
6.48E-134	0.335405	0.752	0.302	1.30E-130	2	LMO7
6.36E-133	0.400772	1	0.98	1.27E-129	2	COL1A1
9.48E-133	0.266037	0.982	0.79	1.90E-129	2	COMP
3.74E-132	0.463379	0.779	0.347	7.49E-129	2	CTGF
4.49E-132	0.320905	0.894	0.534	8.97E-129	2	CDC42EP3
5.57E-128	0.272966	0.976	0.761	1.11E-124	2	STAT1
7.21E-125	0.353046	0.968	0.78	1.44E-121	2	TPM1
6.07E-122	0.268372	0.864	0.526	1.21E-118	2	C1orf198
2.64E-121	0.338323	1	0.998	5.28E-118	2	ACTB
1.18E-116	0.480446	0.957	0.718	2.36E-113	2	TGFBI
9.19E-114	0.335628	0.854	0.52	1.84E-110	2	COL8A1
2.31E-112	0.273462	1	0.989	4.63E-109	2	COL1A2
7.97E-111	0.266076	0.97	0.749	1.59E-107	2	FBLN5
3.31E-106	0.426652	0.991	0.89	6.63E-103	2	ELN
7.28E-103	0.393455	0.631	0.174	1.46E-99	2	ANKRD1
1.14E-102	0.257245	0.902	0.617	2.29E-99	2	PALLD
2.48E-98	0.255406	0.716	0.287	4.96E-95	2	SYNPO2
5.79E-98	0.26991	0.999	0.939	1.16E-94	2	DPYSL2
1.13E-88	0.350105	0.941	0.715	2.26E-85	2	ADM
3.37E-87	0.250925	0.897	0.656	6.74E-84	2	IRS2
3.66E-85	0.309344	0.775	0.437	7.32E-82	2	PRSS23
1.23E-80	0.37278	0.976	0.773	2.46E-77	2	ID3
8.35E-75	0.282763	0.961	0.816	1.67E-71	2	IGFBP7
4.43E-74	0.290465	0.961	0.764	8.87E-71	2	ATF5
4.37E-72	0.281554	0.958	0.865	8.73E-69	2	COL12A1
2.49E-67	0.366134	0.817	0.494	4.98E-64	2	CCL2
8.40E-65	0.339096	0.667	0.296	1.68E-61	2	CEMIP
1.95E-63	0.391793	0.856	0.662	3.91E-60	2	IGFBP3

6.22E-61	0.277021	0.836	0.561	1.24E-57	2	ID2
7.95E-59	0.448548	0.879	0.69	1.59E-55	2	TIMP3
2.21E-58	0.434227	0.595	0.197	4.42E-55	2	PPP1R14A
4.41E-54	0.388498	0.625	0.274	8.81E-51	2	VEPH1
4.90E-46	0.266862	0.96	0.856	9.80E-43	2	TAGLN
1.80E-39	0.524926	0.594	0.231	3.60E-36	2	STMN2
1.34E-34	0.259407	0.571	0.199	2.68E-31	2	TNFRSF11B
6.17E-28	0.501401	1	1	1.23E-24	2	MT2A
0	0.899658	1	0.999	0	3	MT1E
0	0.789956	1	1	0	3	MT1X
5.08E-288	1.348281	0.976	0.962	1.02E-284	3	MT1M
8.34E-184	0.322058	1	1	1.67E-180	3	MT2A
9.93E-144	1.126417	0.797	0.787	1.99E-140	3	MT1F
5.60E-125	0.383168	1	0.999	1.12E-121	3	GPX3
6.60E-110	1.878376	0.839	0.826	1.32E-106	3	MT1G
2.56E-80	1.373106	0.818	0.833	5.12E-77	3	SAA1
5.07E-79	0.306364	0.963	0.932	1.01E-75	3	SPOCK1
1.87E-76	0.30177	0.958	0.902	3.74E-73	3	CRLF1
3.66E-76	0.290936	0.946	0.932	7.33E-73	3	TSC22D3
4.44E-71	0.443848	0.977	0.939	8.89E-68	3	MGP
1.07E-49	0.26192	0.629	0.687	2.14E-46	3	SLC39A8
1.93E-46	0.321144	0.915	0.828	3.87E-43	3	PTX3
1.26E-32	0.332592	0.915	0.855	2.53E-29	3	IGFBP6
2.66E-21	0.284246	0.898	0.905	5.31E-18	3	ELN
3.88E-11	0.267295	0.57	0.734	7.76E-08	3	MT1A
9.01E-11	0.928645	0.316	0.605	1.80E-07	3	SAA2
1.11E-10	0.26524	0.49	0.638	2.22E-07	3	SCARA5
8.83E-06	0.36679	0.344	0.59	0.017654	3	IL1RL1
6.09E-05	0.282145	0.613	0.814	0.121758	3	IGF1
0	1.275375	1	0.988	0	4	SERPINF1
0	0.985403	1	0.985	0	4	C1R
0	0.96449	1	0.997	0	4	CD63
0	0.929817	0.996	0.865	0	4	PCOLCE
0	0.860069	1	0.955	0	4	HSP90B1
0	0.856242	0.996	0.96	0	4	HSP90AA1
0	0.809431	1	0.988	0	4	PPIB
0	0.772499	0.999	0.981	0	4	PRDX1
0	0.683083	1	0.981	0	4	GSTP1

1.15E-304	0.810812	0.995	0.906	2.30E-301	4	HLA-A
2.42E-298	0.850432	1	0.983	4.84E-295	4	SERPING1
1.82E-282	0.774947	1	0.994	3.64E-279	4	ANXA2
5.90E-282	0.714213	0.996	0.966	1.18E-278	4	TMEM59
1.38E-279	0.784391	0.999	0.99	2.77E-276	4	NNMT
6.78E-278	0.782795	0.988	0.832	1.36E-274	4	CTSL
7.18E-277	0.68457	0.995	0.971	1.44E-273	4	UBB
2.74E-274	0.627654	0.995	0.93	5.48E-271	4	PSMB1
5.14E-273	0.639296	0.998	0.977	1.03E-269	4	LAPTM4A
2.92E-272	0.725637	0.986	0.905	5.84E-269	4	EPHX1
1.89E-268	0.647958	0.984	0.89	3.77E-265	4	PSMB3
2.70E-262	0.707626	0.984	0.919	5.40E-259	4	PRDX4
5.32E-256	0.62029	0.99	0.924	1.06E-252	4	MYL12B
3.15E-246	0.598473	0.982	0.88	6.30E-243	4	SSB
6.22E-242	0.609054	0.983	0.938	1.24E-238	4	NDUFC2
2.21E-240	0.590222	1	0.998	4.43E-237	4	MYL6
7.98E-237	0.638599	1	0.978	1.60E-233	4	UBC
6.28E-236	0.570359	0.984	0.895	1.26E-232	4	PSMA4
1.58E-220	0.605255	1	1	3.16E-217	4	MGST1
9.55E-218	1.129178	1	0.988	1.91E-214	4	TIMP1
7.46E-214	0.476843	1	0.998	1.49E-210	4	S100A11
1.73E-211	0.499277	0.976	0.867	3.47E-208	4	PSMB6

2.09E-207	0.508917	0.983	0.887	4.17E-204	4	RPN2
1.27E-205	0.551713	0.986	0.903	2.54E-202	4	ATP5F1B
8.20E-205	0.554385	0.993	0.959	1.64E-201	4	TALDO1
1.43E-202	0.586396	0.983	0.907	2.85E-199	4	HLA-C
3.64E-200	0.61374	0.996	0.943	7.28E-197	4	HSPA5
2.17E-194	0.46738	0.957	0.756	4.34E-191	4	PSME2
2.21E-194	0.528221	0.959	0.887	4.41E-191	4	LGALS3BP
1.62E-190	0.466507	0.944	0.798	3.23E-187	4	PSMA3
5.35E-189	1.018351	0.963	0.881	1.07E-185	4	APOD
1.80E-187	0.666991	0.992	0.951	3.60E-184	4	LGALS3
8.64E-187	0.46174	0.972	0.861	1.73E-183	4	EMC7
1.42E-186	0.477174	0.905	0.706	2.85E-183	4	MDH1
1.74E-184	0.580598	0.984	0.94	3.48E-181	4	ETFB
5.74E-184	0.453453	0.921	0.717	1.15E-180	4	PSMD7
6.35E-180	0.542692	0.987	0.922	1.27E-176	4	MYL12A
5.94E-178	0.430754	0.941	0.787	1.19E-174	4	TECR
6.39E-176	0.494845	0.971	0.847	1.28E-172	4	RNH1
1.67E-174	0.60352	0.995	0.987	3.35E-171	4	MGST3
1.81E-170	0.589672	0.983	0.929	3.63E-167	4	EMP3
2.68E-169	0.540827	0.988	0.946	5.37E-166	4	ANXA1
2.80E-164	0.879246	0.99	0.942	5.60E-161	4	MFAP5
1.86E-163	0.631836	0.984	0.936	3.72E-160	4	AKR1C3

1.88E-163	0.604797	0.966	0.911	3.75E-160	4	NQO1
1.57E-156	0.418218	0.983	0.923	3.14E-153	4	BCAP31
8.98E-154	0.444662	0.849	0.57	1.80E-150	4	NUCB2
2.50E-152	0.404454	0.96	0.836	5.01E-149	4	REEP5
3.33E-147	0.444879	0.989	0.957	6.67E-144	4	CALM1
5.45E-146	0.720407	0.999	0.988	1.09E-142	4	DCN
9.09E-146	0.80785	0.929	0.839	1.82E-142	4	CLU
2.95E-144	0.361175	0.868	0.682	5.90E-141	4	ERP44
7.44E-143	0.463015	0.999	0.994	1.49E-139	4	TXN
1.38E-142	0.381708	0.891	0.715	2.77E-139	4	DHRS7
7.05E-140	0.454865	0.988	0.944	1.41E-136	4	ENO1
1.85E-134	0.315262	0.829	0.647	3.69E-131	4	C1GALT1C1
7.95E-134	0.624036	0.986	0.962	1.59E-130	4	AKR1C1
2.09E-133	0.51	0.999	0.99	4.18E-130	4	SRPX
2.59E-133	0.401699	0.915	0.755	5.18E-130	4	LMAN1
2.97E-131	0.325468	0.861	0.681	5.93E-128	4	SIL1
3.34E-129	0.402648	0.946	0.846	6.69E-126	4	PRDX2
5.22E-129	0.35597	0.975	0.922	1.04E-125	4	REXO2
1.70E-128	0.352926	0.914	0.733	3.40E-125	4	UQCRC1
3.73E-126	0.40932	0.947	0.827	7.47E-123	4	GSTO1
1.23E-123	0.498087	1	0.995	2.46E-120	4	PRDX6
2.12E-122	0.442804	0.953	0.885	4.23E-119	4	PLPP1

3.31E-122	0.402357	0.9	0.767	6.63E-119	4	TUBB4B
3.72E-122	0.420983	0.922	0.761	7.44E-119	4	ECH1
5.69E-119	0.364528	0.998	0.963	1.14E-115	4	APLP2
9.68E-117	0.335416	0.835	0.613	1.94E-113	4	FKBP11
2.32E-116	0.361735	0.998	0.964	4.64E-113	4	PKM
1.15E-115	0.393082	0.921	0.823	2.31E-112	4	CCPG1
3.36E-115	0.421901	0.996	0.978	6.72E-112	4	ANXA5
5.58E-113	0.31296	0.929	0.82	1.12E-109	4	NOP56
1.71E-107	0.342914	0.986	0.958	3.41E-104	4	CALM2
1.23E-103	0.372859	0.841	0.712	2.45E-100	4	STEAP1
5.51E-101	0.34782	0.865	0.72	1.10E-97	4	PTGR1
1.24E-100	0.3517	1	0.981	2.48E-97	4	CALD1
6.97E-98	0.341606	0.832	0.616	1.39E-94	4	BLVRB
8.71E-98	0.4359	0.972	0.887	1.74E-94	4	TUBA1B
2.35E-97	0.606414	0.99	0.94	4.69E-94	4	GSN
1.21E-96	0.287393	0.831	0.68	2.42E-93	4	AIMP1
9.11E-95	0.317228	0.871	0.765	1.82E-91	4	C3
1.64E-94	0.308582	0.897	0.819	3.28E-91	4	CCT5
2.36E-93	0.299981	0.776	0.532	4.72E-90	4	GSTM3
2.99E-93	0.311994	0.94	0.825	5.98E-90	4	PGK1
1.77E-87	0.497917	0.996	0.989	3.55E-84	4	CYB5A
2.62E-87	0.351088	0.996	0.975	5.24E-84	4	CD248
9.15E-87	0.463536	0.93	0.866	1.83E-83	4	FDPS
1.28E-82	0.33389	0.796	0.706	2.57E-79	4	ACAT2
1.30E-82	0.354342	1	1	2.60E-79	4	S100A6
9.14E-82	0.348247	0.993	0.959	1.83E-78	4	HSPA8
2.71E-81	0.28102	0.96	0.917	5.41E-78	4	NME1
4.45E-81	0.601021	0.952	0.91	8.89E-78	4	DPT
2.08E-78	0.427919	0.808	0.637	4.16E-75	4	TUBA1A
2.93E-78	0.410442	0.998	0.995	5.87E-75	4	DBI
8.37E-78	0.38119	0.934	0.861	1.67E-74	4	AKR1B1
7.43E-77	0.620074	0.783	0.691	1.49E-73	4	HP
8.97E-77	0.362472	0.815	0.7	1.79E-73	4	IFITM1
1.51E-76	0.292375	0.847	0.717	3.03E-73	4	SLC3A2

3.11E-75	0.377781	0.94	0.842	6.22E-72	4	HLA-B
3.42E-74	0.292603	0.999	0.995	6.83E-71	4	HSPB1
6.52E-74	0.267322	0.812	0.666	1.30E-70	4	RCN3
8.19E-74	0.30719	0.89	0.794	1.64E-70	4	MANF
2.28E-73	0.381058	0.829	0.7	4.57E-70	4	PHGDH
1.91E-72	0.476778	0.995	0.975	3.82E-69	4	PLIN2
6.51E-71	0.360086	0.935	0.874	1.30E-67	4	NEXN
8.76E-71	0.333627	0.96	0.911	1.75E-67	4	OLFML3
6.63E-70	0.360477	0.927	0.825	1.33E-66	4	TMEM45A
1.09E-69	0.262254	0.8	0.642	2.18E-66	4	P4HA2
3.13E-67	0.524846	0.982	0.962	6.25E-64	4	CFD
3.80E-67	0.377079	0.725	0.574	7.60E-64	4	PLTP
5.02E-62	0.400298	0.856	0.72	1.00E-58	4	LUM
1.55E-54	0.323037	1	0.998	3.10E-51	4	ACTB
1.03E-50	0.341967	0.999	0.989	2.06E-47	4	CAV1
1.98E-50	0.337049	0.992	0.987	3.96E-47	4	TPM2
6.23E-49	0.253164	0.796	0.644	1.25E-45	4	CLEC2B
1.16E-48	0.375948	0.994	0.986	2.32E-45	4	S100A10
1.50E-47	0.269508	0.784	0.674	2.99E-44	4	EMP2
1.64E-46	0.360228	0.976	0.92	3.28E-43	4	FBLN1
9.50E-45	0.332682	0.844	0.796	1.90E-41	4	AKR1C2
5.22E-44	0.345071	0.941	0.883	1.04E-40	4	EFEMP1
7.79E-44	0.425956	0.944	0.95	1.56E-40	4	CIDEC
1.21E-37	0.847912	0.939	0.944	2.43E-34	4	MGP
1.50E-37	0.316435	0.986	0.972	3.00E-34	4	ADH1B
4.81E-37	0.263567	0.86	0.813	9.62E-34	4	GPNMB
8.05E-36	0.409455	0.891	0.844	1.61E-32	4	CTSK
3.11E-35	0.291872	0.999	0.987	6.21E-32	4	CCDC80
1.17E-29	0.265201	0.998	0.975	2.33E-26	4	MMP2
2.16E-29	0.48669	0.977	0.963	4.33E-26	4	CRYAB
4.22E-27	0.288494	0.668	0.609	8.44E-24	4	UCHL1
1.32E-25	0.60797	0.815	0.817	2.64E-22	4	COMP
8.59E-19	0.305977	0.951	0.938	1.72E-15	4	S100A4
4.75E-18	0.253823	0.795	0.731	9.51E-15	4	RARRES2
2.51E-17	0.30304	0.886	0.869	5.02E-14	4	TAGLN
1.01E-14	0.39412	0.884	0.86	2.02E-11	4	IGFBP6
2.75E-14	0.544921	0.818	0.832	5.50E-11	4	SAA1
4.51E-13	0.262707	0.914	0.9	9.02E-10	4	PLA2G16
3.05E-07	1.116447	0.987	0.989	0.000611	4	FABP4
0.000655	0.62539	0.841	0.855	1	4	FABP5
0.005349	0.360662	0.829	0.85	1	4	G0S2
2.05E-64	0.577235	0.892	0.433	4.11E-61	5	PHKG1
1.22E-56	0.501737	0.884	0.569	2.43E-53	5	THBS1
9.46E-56	0.390151	0.997	0.982	1.89E-52	5	FN1

4.61E-54	0.250212	0.155	0.326	9.22E-51	5	PCDH7
2.64E-43	0.250649	0.218	0.462	5.27E-40	5	MYO1B
1.99E-40	0.320335	1	0.982	3.98E-37	5	COL1A1
3.48E-40	0.306765	0.237	0.317	6.96E-37	5	FST
1.77E-38	0.645468	0.984	0.578	3.55E-35	5	DKK1
2.47E-38	0.383835	0.211	0.312	4.94E-35	5	EDIL3
1.02E-37	0.277416	0.282	0.325	2.05E-34	5	VEPH1
3.34E-37	0.260292	0.289	0.513	6.68E-34	5	VGLL3
1.21E-36	0.279673	1	0.99	2.42E-33	5	COL1A2
5.37E-35	0.28966	0.274	0.414	1.07E-31	5	TGFB1I1
1.55E-34	0.517478	0.858	0.747	3.09E-31	5	TGFBI
8.19E-33	0.337622	0.239	0.277	1.64E-29	5	KRT7
1.42E-32	0.527715	0.803	0.945	2.84E-29	5	MYL9
4.39E-32	0.287164	0.3	0.418	8.78E-29	5	SLC16A3
5.86E-31	0.374812	0.324	0.491	1.17E-27	5	NDUFA4L2
1.42E-30	0.289477	0.795	0.391	2.84E-27	5	CTGF
1.56E-30	0.369614	0.963	0.997	3.12E-27	5	LDHA
1.43E-27	0.278625	0.192	0.309	2.86E-24	5	ALDH1B1
3.45E-27	0.400195	0.826	0.636	6.90E-24	5	SERPINE2
2.72E-26	0.305742	0.953	0.996	5.44E-23	5	ANXA2
4.11E-26	0.258904	0.366	0.595	8.21E-23	5	MAGED1
5.34E-26	0.426255	0.945	0.446	1.07E-22	5	VSIR
1.07E-25	0.323194	0.289	0.368	2.14E-22	5	LMO7
1.33E-25	0.317969	0.987	0.696	2.66E-22	5	ALCAM
3.14E-25	0.330163	0.334	0.584	6.28E-22	5	C1orf198
7.55E-24	0.33542	0.434	0.691	1.51E-20	5	RCN3
2.80E-23	0.259325	0.392	0.563	5.61E-20	5	PHACTR2
7.00E-22	0.564553	0.713	0.465	1.40E-18	5	SERPINE1
1.06E-21	0.377919	0.782	0.557	2.11E-18	5	COL8A1
1.15E-20	0.330438	0.884	0.741	2.29E-17	5	ADM
1.09E-19	0.34217	0.25	0.251	2.17E-16	5	TNFRSF11B
1.61E-19	0.25388	0.461	0.602	3.23E-16	5	TMEM204
1.67E-19	0.270459	0.347	0.428	3.34E-16	5	TNFRSF12A
2.11E-19	0.433502	0.782	0.946	4.22E-16	5	TPM4
3.08E-19	0.333814	0.374	0.503	6.16E-16	5	DDAH1
6.75E-18	0.668205	0.955	0.696	1.35E-14	5	CTNNB1
1.12E-17	0.271739	0.471	0.707	2.25E-14	5	HEG1
1.27E-17	0.48121	0.747	0.873	2.55E-14	5	LOX
3.07E-17	0.291952	0.434	0.694	6.14E-14	5	PGM1
7.99E-17	0.261248	0.363	0.552	1.60E-13	5	LINC01503
8.74E-17	0.497616	0.963	0.834	1.75E-13	5	PTX3
1.82E-16	0.28461	0.395	0.435	3.64E-13	5	GPR176
1.83E-16	0.290797	0.374	0.593	3.65E-13	5	CDC42EP3
8.51E-15	0.367775	0.358	0.557	1.70E-11	5	CSRP1

1.06E-14	0.369111	0.397	0.705	2.12E-11	5	HERPUD1
3.53E-14	0.336123	0.845	0.932	7.07E-11	5	MYL12A
8.42E-14	0.415082	0.353	0.317	1.68E-10	5	SULF1
7.56E-13	0.280711	0.805	0.712	1.51E-09	5	BGN
7.37E-12	0.328472	0.382	0.448	1.47E-08	5	GRAMD2B
1.12E-11	0.369354	0.734	0.598	2.24E-08	5	CYR61
1.58E-11	0.283453	0.984	0.693	3.16E-08	5	PSAT1
1.59E-10	0.433298	0.992	0.979	3.19E-07	5	ANXA5
3.24E-10	0.260929	0.945	0.818	6.48E-07	5	VAT1
3.32E-10	0.424643	0.366	0.566	6.65E-07	5	AXL
3.61E-10	0.337861	0.655	0.329	7.22E-07	5	GREM1
6.27E-10	0.299002	0.818	0.967	1.25E-06	5	CALM2
7.26E-10	0.286577	0.847	0.749	1.45E-06	5	RAD23B
7.43E-10	0.446846	0.737	0.928	1.49E-06	5	CTSB
1.37E-09	0.356715	0.913	0.952	2.74E-06	5	CD59
1.60E-09	0.481184	0.384	0.488	3.21E-06	5	CRIM1
1.69E-09	0.317476	0.797	0.882	3.38E-06	5	COL12A1
2.73E-09	0.38446	0.374	0.314	5.46E-06	5	MDK
7.28E-09	0.268573	0.411	0.683	1.46E-05	5	LOXL2
9.84E-08	0.258385	1	0.984	0.000197	5	LAMC1
1.36E-07	0.397262	0.587	0.651	0.000272	5	ELL2
6.53E-07	0.263068	0.792	0.878	0.001306	5	GNG11
1.15E-06	0.506786	0.929	0.859	0.0023	5	IGFBP6
1.78E-06	0.332844	0.637	0.658	0.003569	5	PALLD
2.02E-06	0.268432	0.945	0.945	0.004046	5	MPC2
2.39E-06	0.251427	0.811	0.84	0.004771	5	GSTO1
2.83E-06	0.260695	0.997	0.955	0.005654	5	MAP1B
5.15E-06	0.29502	0.624	0.436	0.010309	5	AEBP1
6.32E-06	0.256637	0.447	0.525	0.012645	5	LTBP2
9.25E-06	0.345912	0.45	0.758	0.018495	5	ACTN1
1.06E-05	0.367158	0.982	0.889	0.021298	5	LGALS3BP
1.45E-05	0.287944	0.992	0.738	0.028974	5	FBN2
2.76E-05	0.296428	0.979	0.875	0.055171	5	NEXN
6.41E-05	0.33674	0.921	0.823	0.128166	5	ITGB5
0.000126	0.284853	0.674	0.911	0.252957	5	DAB2
0.00018	0.384702	0.989	0.832	0.360206	5	TWIST2
0.000182	0.287038	0.495	0.837	0.364732	5	PGRMC2
0.000195	0.398397	0.503	0.438	0.389782	5	C12orf75
0.00027	0.278412	0.505	0.885	0.540901	5	SLC38A2
0.000303	0.366378	0.434	0.245	0.605914	5	INHBA
0.000389	0.267278	0.984	0.891	0.77864	5	CSF1
0.000397	0.294802	0.663	0.797	0.793755	5	THBS2
0.000656	0.308322	0.926	0.545	1	5	CCNL1
0.000676	0.303855	0.937	0.743	1	5	GARS

0.001178	0.336787	0.984	0.829	1	5	CAV2
0.001227	0.259513	0.968	0.949	1	5	ANXA1
0.003951	0.495218	0.997	0.958	1	5	HTRA1
0.004286	0.355135	0.495	0.273	1	5	STMN2
0.004371	0.260314	0.966	0.653	1	5	MYC
0.004906	0.409635	0.576	0.595	1	5	PDLIM7
0.00837	0.28372	0.992	0.837	1	5	EMP1
0.009117	0.36124	0.992	0.924	1	5	FBN1
2.73E-61	0.758271	0.966	0.996	5.46E-58	6	ANXA2
1.53E-58	0.797283	0.963	0.996	3.07E-55	6	TXN
8.02E-58	0.701969	0.991	0.999	1.60E-54	6	S100A11
8.54E-49	0.48938	1	1	1.71E-45	6	GAPDH
8.21E-46	0.880765	0.938	0.99	1.64E-42	6	NPM1
8.29E-42	0.589901	0.988	0.999	1.66E-38	6	MYL6
8.44E-42	0.685338	0.953	0.984	1.69E-38	6	GSTP1
1.61E-38	0.835429	0.938	0.991	3.23E-35	6	FABP4
2.71E-38	0.783674	0.898	0.983	5.42E-35	6	ANXA5
2.47E-37	0.4742	0.997	1	4.93E-34	6	S100A6
1.08E-34	0.82581	0.832	0.855	2.16E-31	6	FABP5
1.60E-29	0.354658	1	1	3.21E-26	6	LGALS1
4.00E-29	0.643057	0.963	0.997	8.01E-26	6	LDHA
8.02E-29	0.67035	0.888	0.977	1.60E-25	6	UBB
8.51E-29	0.705114	0.969	0.996	1.70E-25	6	DBI
6.37E-26	0.518757	0.913	0.99	1.27E-22	6	TPM2
9.92E-25	0.556504	0.801	0.935	1.98E-21	6	MYL12B
1.84E-24	0.622199	0.922	0.991	3.68E-21	6	MGST3
1.14E-22	0.530951	0.947	0.998	2.28E-19	6	PRDX6
2.16E-22	0.823087	0.848	0.954	4.32E-19	6	ANXA1
2.26E-22	0.695514	0.873	0.966	4.52E-19	6	TALDO1
1.15E-21	0.335866	0.217	0.494	2.31E-18	6	NDUFA4L2
8.74E-21	0.574603	0.913	0.99	1.75E-17	6	S100A10
9.20E-21	0.785379	0.913	0.986	1.84E-17	6	PRDX1
4.17E-20	0.531196	0.981	0.999	8.34E-17	6	ACTB
4.18E-19	0.721631	0.795	0.873	8.35E-16	6	TAGLN
2.74E-17	0.318464	1	1	5.48E-14	6	FTL
6.75E-16	0.703276	0.811	0.92	1.35E-12	6	NQO1
8.85E-14	0.768422	0.817	0.914	1.77E-10	6	ATP5F1B
3.81E-13	0.347577	0.972	0.999	7.61E-10	6	SH3BGRL3
5.51E-13	0.483809	0.789	0.933	1.10E-09	6	MYL12A
2.27E-12	0.300738	0.655	0.613	4.53E-09	6	UCP2
4.71E-11	0.36474	0.944	0.997	9.43E-08	6	HSPB1
7.92E-10	0.292249	0.991	1	1.58E-06	6	MGST1
5.47E-09	0.474564	0.854	0.953	1.09E-05	6	CIDEC
2.34E-08	0.521471	0.643	0.863	4.67E-05	6	PRDX2

1.23E-07	0.407997	0.742	0.956	0.000246	6	ENO1
1.36E-07	0.411219	0.801	0.945	0.000272	6	AKR1C3
1.41E-07	0.523677	0.882	0.967	0.000283	6	CRYAB
1.43E-07	0.459147	0.233	0.308	0.000286	6	ADIRF
3.19E-07	0.333434	0.295	0.5	0.000638	6	SRGN
4.08E-07	0.264881	0.683	0.805	0.000816	6	AKR1C2
8.69E-07	0.438907	0.761	0.945	0.001737	6	MYL9
2.84E-06	0.616355	0.78	0.932	0.005684	6	REXO2
6.64E-06	0.483579	0.919	0.992	0.013279	6	CYB5A
3.86E-05	0.251197	0.717	0.878	0.077199	6	FDPS
3.99E-05	0.567751	0.885	0.993	0.079864	6	TIMP1
8.13E-05	0.315668	0.739	0.965	0.162623	6	MAP1B
0.000138	0.252574	0.798	0.954	0.276582	6	IGFBP5
0.000195	0.479763	0.758	0.968	0.389418	6	CALM2
0.000896	0.315795	0.609	0.729	1	6	MDH1
0.000922	0.476232	0.72	0.976	1	6	PKM
0.000967	0.388224	0.817	0.96	1	6	LGALS3
0.001524	0.53726	0.472	0.698	1	6	CCND1
0.002662	0.486887	0.438	0.621	1	6	UCHL1
0.002742	0.281041	0.811	0.944	1	6	S100A4
0.005782	0.302206	0.807	0.95	1	6	MPC2
0.009232	0.426301	0.677	0.658	1	6	ACAA2
3.18E-79	0.919904	0.691	0.137	6.36E-76	7	PCK1
6.18E-79	1.816122	0.992	0.661	1.24E-75	7	GPD1
2.18E-78	1.36555	1	0.564	4.36E-75	7	AGPAT2
1.65E-77	1.853011	1	0.819	3.29E-74	7	PLIN4
3.62E-77	1.512786	1	0.77	7.25E-74	7	AOC3
4.09E-77	1.647701	1	0.832	8.18E-74	7	FASN
4.93E-77	1.363165	1	0.95	9.86E-74	7	ACSL1
7.22E-76	1.66565	1	0.728	1.44E-72	7	PLIN1
2.71E-75	2.498258	1	0.892	5.42E-72	7	SCD
6.03E-75	1.498684	1	0.954	1.21E-71	7	ACACB
1.67E-74	1.338665	1	0.855	3.35E-71	7	PNPLA2
3.91E-73	1.408534	1	0.9	7.82E-70	7	PLA2G16
5.38E-73	2.501698	0.992	0.846	1.08E-69	7	G0S2
3.62E-72	1.055187	1	0.895	7.24E-69	7	SLC25A1
9.27E-70	1.011981	0.976	0.636	1.85E-66	7	HK2
2.19E-69	2.097375	0.992	0.852	4.38E-66	7	FABP5
7.01E-68	1.175626	0.976	0.676	1.40E-64	7	FAM213A
1.59E-67	0.898384	1	0.794	3.19E-64	7	VKORC1L1
5.19E-67	1.048275	0.976	0.692	1.04E-63	7	AQP7
5.75E-67	0.944152	0.919	0.517	1.15E-63	7	LGALS12
5.85E-67	0.852258	0.976	0.706	1.17E-63	7	MLXIPL
9.18E-66	0.991281	1	0.866	1.84E-62	7	HEBP2

9.71E-66	1.137104	0.976	0.609	1.94E-62	7	UCP2
1.09E-65	1.466316	1	0.86	2.19E-62	7	INSIG1
1.05E-64	1.748895	1	0.989	2.11E-61	7	FABP4
1.74E-64	1.16058	1	0.995	3.48E-61	7	DBI
2.92E-64	1.02915	1	0.949	5.84E-61	7	CIDEC
4.45E-64	0.772657	0.967	0.648	8.90E-61	7	ITGA7
8.43E-63	0.851152	0.984	0.736	1.69E-59	7	ERV3-1
1.65E-62	0.90776	1	0.892	3.29E-59	7	COL4A1
2.07E-62	0.95954	0.951	0.632	4.14E-59	7	APOE
4.36E-62	1.524723	0.976	0.824	8.72E-59	7	GPAM
1.29E-61	0.825625	0.951	0.585	2.58E-58	7	HADH
3.69E-60	0.870389	1	0.94	7.39E-57	7	COL4A2
1.03E-59	0.718603	0.911	0.571	2.06E-56	7	PRKAR2B
7.74E-59	0.80222	0.992	0.851	1.55E-55	7	FZD4
1.21E-57	0.924493	0.967	0.716	2.42E-54	7	RDH5
8.69E-56	0.696595	0.967	0.785	1.74E-52	7	CS
2.26E-55	0.647774	0.984	0.686	4.51E-52	7	MRAS
2.77E-55	0.588757	0.927	0.398	5.55E-52	7	PTPRF
2.10E-54	0.660519	0.984	0.79	4.20E-51	7	SORT1
8.85E-54	0.971491	0.911	0.671	1.77E-50	7	DGAT2
2.06E-53	0.758614	0.992	0.864	4.12E-50	7	MKNK2
6.73E-53	0.783563	0.886	0.55	1.35E-49	7	DLAT
9.33E-52	1.005314	0.943	0.837	1.87E-48	7	ACLY
2.70E-51	1.938244	0.805	0.298	5.40E-48	7	ADIRF
3.59E-51	0.557121	1	0.999	7.18E-48	7	MT-CO2
5.02E-51	1.486741	0.911	0.705	1.00E-47	7	LPL
8.57E-50	0.587434	0.902	0.64	1.71E-46	7	NR1H3
2.00E-49	0.603024	0.829	0.43	4.00E-46	7	ZBED3-AS1
2.97E-48	0.719796	1	0.944	5.95E-45	7	MPC2
3.36E-48	1.161662	0.992	0.892	6.73E-45	7	FADS1
4.87E-48	0.807636	0.976	0.799	9.74E-45	7	AKAP12
5.08E-48	0.620663	0.951	0.741	1.02E-44	7	C19orf12
5.86E-48	0.679259	0.992	0.833	1.17E-44	7	CAV2
1.35E-47	0.767763	0.959	0.592	2.70E-44	7	HILPDA
6.52E-46	0.649793	1	0.945	1.30E-42	7	SHC1
1.54E-45	0.774415	1	0.989	3.07E-42	7	CYB5A
1.66E-45	0.526614	0.911	0.641	3.32E-42	7	C2CD2
7.10E-44	0.675624	0.992	0.889	1.42E-40	7	SH3PXD2A
4.42E-43	0.537203	0.813	0.503	8.84E-40	7	CLMN
7.13E-43	0.630967	0.886	0.697	1.43E-39	7	AACS
1.27E-42	0.669565	0.797	0.498	2.54E-39	7	CEBPA
1.35E-42	0.528056	0.959	0.804	2.71E-39	7	TSPAN14
1.87E-42	0.582751	0.976	0.718	3.74E-39	7	ADAM12
2.82E-42	0.624935	0.976	0.834	5.64E-39	7	IDI1

6.14E-42	0.717697	0.862	0.661	1.23E-38	7	ACSS2
1.71E-41	0.453848	0.772	0.394	3.41E-38	7	GPT2
1.70E-40	0.457541	0.919	0.72	3.40E-37	7	CSAD
3.48E-40	0.671654	1	0.989	6.96E-37	7	SAT1
1.06E-39	0.566047	0.992	0.961	2.13E-36	7	ELOVL5
2.20E-38	0.630362	0.951	0.709	4.40E-35	7	FHL1
2.96E-38	0.514121	0.992	0.93	5.91E-35	7	DHCR24
3.15E-38	0.474293	0.683	0.304	6.30E-35	7	LIPE
3.74E-38	0.691559	0.854	0.636	7.48E-35	7	CDKN2C
3.35E-37	0.50778	0.943	0.802	6.69E-34	7	LPIN1
3.85E-37	1.121598	0.772	0.535	7.70E-34	7	RBP4
8.15E-37	0.42542	0.829	0.578	1.63E-33	7	SHMT1
2.17E-36	0.508605	0.902	0.572	4.35E-33	7	GLIPR2
5.45E-36	0.373889	0.675	0.317	1.09E-32	7	CNTFR
7.44E-36	0.397033	0.78	0.39	1.49E-32	7	BOK
7.99E-36	0.474614	0.87	0.724	1.60E-32	7	RETSAT
1.21E-35	1.254888	0.829	0.641	2.43E-32	7	CD36
3.26E-35	0.415027	0.732	0.428	6.51E-32	7	PDE3B
5.42E-35	0.868968	0.854	0.698	1.08E-31	7	HP
2.05E-34	0.461492	0.846	0.646	4.11E-31	7	FNDC4
2.28E-34	0.617212	0.862	0.706	4.57E-31	7	ACSL5
3.02E-34	0.484754	0.837	0.588	6.04E-31	7	RASSF4
5.92E-34	0.433268	0.902	0.708	1.18E-30	7	ACOX1
9.55E-33	0.443506	0.951	0.679	1.91E-29	7	PGM1
9.78E-33	0.624307	0.87	0.723	1.96E-29	7	HMGCS1
2.88E-32	0.512812	0.911	0.753	5.76E-29	7	RHOB
1.80E-31	0.617719	0.829	0.66	3.60E-28	7	MVD
3.35E-31	0.45629	0.959	0.811	6.70E-28	7	TNS1
7.84E-31	0.364077	0.642	0.336	1.57E-27	7	GYG2
1.04E-30	0.422355	1	0.99	2.07E-27	7	NEAT1
2.37E-30	0.515528	0.691	0.414	4.73E-27	7	ALDH1L1
2.39E-30	0.464751	0.756	0.565	4.78E-27	7	PALMD
3.46E-30	0.443852	0.846	0.706	6.93E-27	7	SIK2
5.06E-30	0.323523	0.829	0.662	1.01E-26	7	PPARG
1.12E-29	0.486976	0.789	0.608	2.24E-26	7	ZBED3
2.95E-29	0.447218	1	0.996	5.90E-26	7	PRDX6
3.98E-29	0.387467	0.862	0.715	7.96E-26	7	AIFM2
5.76E-29	0.432754	0.919	0.799	1.15E-25	7	AKR1C2
7.98E-29	0.343019	0.642	0.331	1.60E-25	7	ACSF2
1.07E-28	0.439063	0.829	0.662	2.13E-25	7	TSKU
1.41E-27	0.478707	1	0.99	2.83E-24	7	CAV1
4.10E-27	0.359697	0.634	0.359	8.21E-24	7	CHCHD10
1.08E-26	0.421543	0.87	0.762	2.17E-23	7	LSS
2.68E-26	0.395017	0.87	0.733	5.35E-23	7	FAH

2.02E-25	0.36838	0.707	0.508	4.03E-22	7	DGAT1
4.24E-25	0.371822	0.959	0.874	8.48E-22	7	GPC1
4.35E-25	0.442972	0.984	0.921	8.71E-22	7	GYPC
9.86E-25	0.375586	0.707	0.509	1.97E-21	7	PIM3
1.64E-24	0.498181	0.927	0.871	3.28E-21	7	FDPS
1.97E-24	0.330553	0.537	0.267	3.93E-21	7	PPP1R16A
4.70E-24	0.431394	0.984	0.893	9.41E-21	7	RHOBTB3
8.97E-24	0.394769	0.748	0.599	1.79E-20	7	CAMK1
4.34E-23	0.365737	0.748	0.625	8.67E-20	7	PDP2
9.87E-23	0.452483	0.919	0.85	1.97E-19	7	FADS2
1.21E-22	0.308538	0.691	0.463	2.42E-19	7	NSDHL
1.44E-22	0.346997	0.878	0.656	2.87E-19	7	ACAA2
4.53E-22	0.263261	0.772	0.5	9.07E-19	7	SCRN2
7.86E-22	0.373646	0.902	0.779	1.57E-18	7	CKS1B
1.04E-21	0.288869	0.626	0.367	2.08E-18	7	SYNPO
5.84E-21	0.30024	0.886	0.807	1.17E-17	7	RASD1
7.48E-21	0.379662	0.87	0.767	1.50E-17	7	MSMO1
1.33E-20	0.274842	0.813	0.732	2.66E-17	7	SYNE3
1.52E-20	0.806567	0.699	0.595	3.04E-17	7	MRAP
3.18E-20	0.253299	0.569	0.32	6.35E-17	7	PFKFB3
5.57E-20	0.320564	1	0.945	1.11E-16	7	LAMA4
6.13E-20	0.301625	1	1	1.23E-16	7	LGALS1
7.27E-20	0.306788	0.927	0.86	1.45E-16	7	PEMT
2.09E-19	0.27436	0.691	0.477	4.17E-16	7	FRMD4A
2.97E-19	0.424813	1	0.964	5.94E-16	7	CFD
7.65E-19	0.364315	0.78	0.713	1.53E-15	7	ACAT2
1.89E-18	0.355627	0.764	0.595	3.78E-15	7	ITIH5
2.03E-18	0.413765	0.984	0.939	4.06E-15	7	STOM
2.33E-18	0.31399	0.732	0.548	4.67E-15	7	CKB
5.41E-18	0.303559	0.772	0.699	1.08E-14	7	SELENBP1
1.21E-17	0.360852	0.967	0.917	2.42E-14	7	C1QTNF1
1.27E-17	0.355111	0.984	0.942	2.55E-14	7	NDUFC2
1.43E-17	0.317309	0.715	0.597	2.87E-14	7	PPIF
1.45E-17	0.27841	0.496	0.274	2.89E-14	7	SEMA3G
1.58E-17	0.266088	0.683	0.498	3.16E-14	7	ECHDC2
3.70E-17	0.307619	0.61	0.468	7.41E-14	7	SREBF1
5.40E-17	0.347277	0.821	0.744	1.08E-13	7	PDK4
2.78E-16	0.341592	0.659	0.572	5.56E-13	7	ZNF117
2.90E-16	0.307652	0.756	0.734	5.79E-13	7	GHR
3.27E-16	0.263551	0.967	0.928	6.54E-13	7	BCAP31
8.13E-16	0.307086	0.992	0.91	1.63E-12	7	ATP5F1B
8.14E-16	0.300463	1	0.983	1.63E-12	7	HSPB6
8.21E-16	0.380757	0.659	0.585	1.64E-12	7	GPBAR1
8.63E-16	0.293516	0.886	0.697	1.73E-12	7	NRN1

1.91E-15	0.271863	0.715	0.575	3.81E-12	7	KANK1
2.55E-15	0.288593	0.805	0.75	5.10E-12	7	LDLR
2.73E-15	0.398681	0.715	0.617	5.45E-12	7	HOXB6
3.19E-15	0.338871	0.943	0.874	6.37E-12	7	LMO4
1.76E-14	0.305651	0.772	0.745	3.52E-11	7	NRCAM
2.12E-14	0.285004	0.472	0.326	4.24E-11	7	PNPLA3
2.51E-14	0.303897	0.642	0.57	5.03E-11	7	GNG2
2.64E-14	0.283378	0.699	0.6	5.29E-11	7	TOB1
8.71E-14	0.256361	0.846	0.8	1.74E-10	7	TECR
1.34E-13	0.268904	0.74	0.685	2.69E-10	7	EBP
1.53E-13	0.438498	0.602	0.536	3.05E-10	7	CDO1
7.90E-13	0.280254	0.789	0.642	1.58E-09	7	SPRY1
1.38E-12	0.335596	0.65	0.611	2.76E-09	7	MMD
1.82E-12	0.34841	0.537	0.448	3.64E-09	7	PLCXD1
2.54E-12	0.252575	0.504	0.369	5.08E-09	7	1-Mar
5.43E-12	0.338888	1	0.921	1.09E-08	7	TKT
8.90E-12	0.312519	0.585	0.51	1.78E-08	7	DUSP6
1.19E-11	0.283224	0.984	0.944	2.37E-08	7	ETFB
8.74E-11	0.287012	0.821	0.813	1.75E-07	7	MT-ND6
2.48E-10	0.315159	0.87	0.643	4.96E-07	7	SOX4
9.62E-10	0.319999	0.537	0.474	1.92E-06	7	TRARG1
4.23E-09	0.287516	0.992	0.988	8.46E-06	7	GLUL
1.07E-08	0.592123	0.618	0.609	2.14E-05	7	APOC1
6.47E-06	0.254392	0.398	0.328	0.012931	7	KCNK3
9.40E-06	0.272058	0.537	0.583	0.018801	7	AL513283.1
1.09E-05	0.301403	0.496	0.51	0.021731	7	LBP
2.60E-05	0.250585	0.512	0.554	0.052049	7	DUSP4
0.004356	0.340784	0.951	0.965	1	7	CRYAB
2.92E-94	0.266035	0.722	0.035	5.85E-91	8	PIMREG
1.84E-80	0.400843	0.685	0.059	3.69E-77	8	MYBL2
2.33E-68	0.30241	0.759	0.101	4.66E-65	8	FAM111B
2.92E-52	0.805906	0.704	0.002	5.84E-49	8	MKI67
2.16E-51	0.253957	0.648	0.002	4.33E-48	8	TROAP
5.30E-36	1.467031	0.963	0.299	1.06E-32	8	PCLAF
7.00E-36	1.477465	1	0.353	1.40E-32	8	STMN1
9.67E-36	0.338139	0.926	0.187	1.93E-32	8	SPC24
2.66E-35	0.318015	0.815	0.21	5.31E-32	8	SHCBP1
2.93E-33	1.406095	1	0.939	5.85E-30	8	H2AFZ
2.09E-32	0.469942	0.963	0.381	4.18E-29	8	HELLS
6.68E-31	0.428846	0.852	0.088	1.34E-27	8	PRC1
6.43E-29	0.751938	0.926	0.453	1.29E-25	8	CENPU
1.63E-28	0.585025	0.778	0.12	3.27E-25	8	CENPM
6.34E-28	1.254565	0.907	0.28	1.27E-24	8	TOP2A
1.34E-27	1.314773	0.981	0.894	2.68E-24	8	TUBA1B

2.71E-27	0.71742	0.889	0.19	5.41E-24	8	TK1
1.92E-26	0.908005	1	0.968	3.84E-23	8	TUBB
2.55E-26	0.45531	0.87	0.307	5.10E-23	8	TACC3
3.93E-26	0.340878	0.63	0.002	7.87E-23	8	CKAP2L
1.57E-25	1.162024	0.741	0.071	3.15E-22	8	RRM2
1.50E-24	0.818262	0.907	0.354	3.00E-21	8	TYMS
1.93E-24	0.315731	0.944	0.445	3.86E-21	8	MCM4
5.76E-24	0.342253	0.815	0.237	1.15E-20	8	CDCA3
3.32E-23	0.31955	0.685	0.118	6.65E-20	8	KIFC1
4.89E-23	0.74532	0.759	0.158	9.79E-20	8	UBE2C
1.52E-22	1.191809	0.87	0.239	3.03E-19	8	PTTG1
8.17E-22	0.665375	0.907	0.344	1.63E-18	8	CENPN
2.06E-21	0.468744	0.722	0.07	4.12E-18	8	GTSE1
2.17E-21	0.603445	0.87	0.306	4.34E-18	8	TMEM106C
2.47E-21	0.704935	0.963	0.532	4.94E-18	8	PCNA
6.48E-21	0.337193	0.796	0.205	1.30E-17	8	UHRF1
6.86E-21	0.322725	0.833	0.393	1.37E-17	8	E2F1
1.25E-20	0.440879	0.833	0.362	2.50E-17	8	RAD51AP1
8.96E-20	1.614452	1	0.958	1.79E-16	8	HIST1H4C
1.01E-19	0.730411	0.963	0.61	2.01E-16	8	PLP2
2.20E-19	0.745272	0.926	0.653	4.40E-16	8	C19orf48
3.29E-19	0.620982	0.852	0.404	6.58E-16	8	UBE2S
5.05E-19	0.446917	0.889	0.476	1.01E-15	8	SLC7A1
1.12E-18	0.337178	0.759	0.086	2.24E-15	8	DIAPH3
1.79E-18	0.440501	0.815	0.398	3.57E-15	8	CCNB1
1.95E-18	0.336892	0.926	0.554	3.91E-15	8	SYNE2
4.02E-18	0.694864	0.981	0.828	8.04E-15	8	TUBA1C
1.49E-17	0.665121	0.981	0.774	2.97E-14	8	TUBB6
2.12E-17	0.589404	0.852	0.43	4.24E-14	8	SMC4
3.07E-17	1.235116	0.852	0.434	6.14E-14	8	HMGB2
3.70E-17	0.388697	0.778	0.324	7.41E-14	8	CDCA5
5.16E-17	0.451361	0.926	0.655	1.03E-13	8	KIF22
5.92E-17	1.027016	0.907	0.534	1.18E-13	8	CKS2
6.08E-17	0.561867	0.963	0.78	1.22E-13	8	CKS1B
1.14E-16	0.313172	0.648	0.182	2.27E-13	8	DEPDC1
1.16E-16	0.288743	0.815	0.361	2.32E-13	8	KIF20B
1.89E-16	0.328145	0.87	0.46	3.78E-13	8	ZWINT
2.63E-16	0.368667	0.815	0.433	5.26E-13	8	KNSTRN
2.90E-16	0.385232	0.741	0.16	5.80E-13	8	CENPH
1.16E-15	0.299418	1	0.66	2.32E-12	8	TMEM97
2.07E-15	0.416206	0.815	0.423	4.14E-12	8	CCBE1
2.50E-15	0.374652	0.833	0.545	5.00E-12	8	PRG4
4.20E-15	0.418099	0.722	0.262	8.39E-12	8	MND1
5.04E-15	0.443807	0.833	0.464	1.01E-11	8	CXCL6

6.95E-15	0.84675	0.944	0.778	1.39E-11	8	TUBB4B
2.71E-14	0.491087	0.889	0.552	5.42E-11	8	DHFR
4.41E-14	0.357941	0.833	0.316	8.83E-11	8	SMTN
4.47E-14	0.306393	0.741	0.295	8.95E-11	8	CDT1
9.32E-14	0.256958	0.944	0.613	1.86E-10	8	CERS6
1.03E-13	0.885969	0.685	0.053	2.06E-10	8	BIRC5
1.93E-13	0.253375	0.833	0.353	3.86E-10	8	ANKH
2.64E-13	0.46625	0.852	0.438	5.28E-10	8	C12orf75
3.10E-13	0.668508	0.722	0.153	6.19E-10	8	CENPF
3.47E-13	0.449494	0.741	0.322	6.94E-10	8	AURKA
3.54E-13	0.55284	0.685	0.177	7.07E-10	8	ANLN
3.87E-13	0.403588	0.685	0.288	7.74E-10	8	HMMR
5.29E-13	0.897525	0.852	0.593	1.06E-09	8	DKK1
7.55E-13	0.392479	0.815	0.404	1.51E-09	8	CKAP2
8.91E-13	0.312632	0.815	0.364	1.78E-09	8	MOXD1
2.63E-12	0.448425	0.741	0.45	5.26E-09	8	FOXM1
3.51E-12	0.313634	1	1	7.02E-09	8	TMSB10
7.59E-12	0.336959	0.944	0.648	1.52E-08	8	CDC25B
2.15E-11	0.364156	0.741	0.238	4.30E-08	8	NUSAP1
2.45E-11	0.259412	0.667	0.157	4.90E-08	8	ASPM
2.75E-11	0.390193	0.815	0.421	5.50E-08	8	CDH2
3.76E-11	0.3797	0.981	0.836	7.52E-08	8	COTL1
3.78E-11	0.400823	0.944	0.71	7.55E-08	8	PHGDH
5.28E-11	0.355909	0.944	0.642	1.06E-07	8	GADD45A
6.64E-11	0.30218	0.926	0.733	1.33E-07	8	ADAMTS5
6.83E-11	0.431758	0.63	0.124	1.37E-07	8	CDC20
1.04E-10	0.632989	0.685	0.235	2.07E-07	8	ANKRD1
3.04E-10	0.446845	1	0.99	6.09E-07	8	CAV1
3.64E-10	0.44693	0.907	0.622	7.27E-07	8	ITGA5
7.22E-10	0.278774	0.593	0.15	1.44E-06	8	KCNMA1
7.87E-10	0.419093	0.648	0.179	1.57E-06	8	PRR11
1.09E-09	0.424772	0.926	0.651	2.19E-06	8	TUBA1A
1.23E-09	0.416182	0.981	0.95	2.47E-06	8	EIF4EBP1
1.26E-09	0.439972	0.796	0.603	2.51E-06	8	CENPW
1.34E-09	0.366978	0.833	0.422	2.69E-06	8	TNFRSF12A
1.55E-09	0.290586	0.685	0.258	3.09E-06	8	CDCA7
1.61E-09	0.408019	0.796	0.466	3.23E-06	8	ARHGAP29
1.75E-09	0.372796	1	0.885	3.51E-06	8	HIF1A
2.56E-09	0.397106	0.926	0.748	5.11E-06	8	FBN2
2.70E-09	0.825244	0.981	0.802	5.40E-06	8	CDKN1A
4.92E-09	0.252229	0.704	0.404	9.83E-06	8	CENPE
5.15E-09	0.41231	0.778	0.59	1.03E-05	8	SCARA3
5.24E-09	0.51034	0.685	0.28	1.05E-05	8	TPX2
5.54E-09	0.303958	0.963	0.732	1.11E-05	8	PTGR1

6.38E-09	0.321181	0.926	0.707	1.28E-05	8	ALCAM
6.46E-09	0.327213	0.722	0.378	1.29E-05	8	FOSL1
1.77E-08	0.660193	0.981	0.989	3.55E-05	8	TIMP1
3.23E-08	0.468792	0.759	0.544	6.47E-05	8	TNC
3.65E-08	0.451392	1	1	7.30E-05	8	MT2A
4.20E-08	0.253362	1	0.88	8.41E-05	8	SLC44A1
5.25E-08	0.395975	0.722	0.366	0.000105	8	UBE2T
8.27E-08	0.265494	0.5	0.003	0.000165	8	CEP55
8.53E-08	0.563787	0.667	0.196	0.000171	8	CDKN3
8.59E-08	0.281312	0.944	0.843	0.000172	8	CRISPLD2
8.76E-08	0.336125	0.722	0.338	0.000175	8	UPP1
8.84E-08	0.735048	1	0.933	0.000177	8	DDIT4
9.17E-08	0.363818	1	0.962	0.000183	8	HSPA8
1.01E-07	0.317909	0.833	0.487	0.000201	8	METRNL
1.06E-07	0.266993	0.648	0.344	0.000213	8	CDCA2
1.11E-07	0.345121	0.963	0.957	0.000223	8	CAVIN1
1.26E-07	0.250528	0.704	0.237	0.000252	8	WEE1
1.45E-07	0.286567	0.833	0.494	0.00029	8	PSMB9
1.63E-07	0.375451	0.907	0.689	0.000325	8	CCND1
1.64E-07	0.362142	0.704	0.39	0.000329	8	ABI3BP
1.66E-07	0.257524	0.667	0.266	0.000332	8	CCNA2
1.79E-07	0.384825	0.889	0.656	0.000359	8	CLEC2B
2.39E-07	0.25607	0.963	0.742	0.000479	8	CREB3L1
3.99E-07	0.30748	0.944	0.908	0.000798	8	CKAP4
4.08E-07	0.286853	1	0.96	0.000816	8	CALM2
4.82E-07	0.271563	0.889	0.664	0.000964	8	ALDH1A3
5.00E-07	0.385928	0.648	0.346	0.000999	8	MXD3
6.38E-07	0.257901	0.741	0.411	0.001276	8	SLC16A3
7.08E-07	0.38296	1	0.964	0.001415	8	MT1M
8.24E-07	0.358407	0.667	0.309	0.001647	8	HMGB3
8.78E-07	0.290153	0.963	0.916	0.001755	8	ANPEP
9.17E-07	0.273603	0.963	0.812	0.001833	8	MT-ND6
9.28E-07	0.275627	0.648	0.344	0.001856	8	AURKB
1.04E-06	0.261242	0.704	0.464	0.002088	8	LY6K
1.48E-06	0.415821	0.889	0.697	0.002956	8	MGLL
1.66E-06	0.336202	1	0.95	0.003312	8	ANXA1
2.17E-06	0.311831	0.907	0.699	0.004345	8	LINC01133
2.52E-06	0.989386	0.852	0.688	0.005033	8	IGFBP3
3.19E-06	0.436207	0.944	0.862	0.006374	8	IGFBP6
3.67E-06	0.258763	0.685	0.328	0.007333	8	RGMB
4.62E-06	0.407044	0.944	0.867	0.009232	8	LOX
5.84E-06	0.355519	0.611	0.189	0.01167	8	TCF19
6.32E-06	0.259583	0.907	0.683	0.012642	8	EMP2
6.98E-06	0.273671	0.889	0.654	0.013953	8	ANKRD28

8.17E-06	0.343256	1	0.998	0.016343	8	ACTB
1.34E-05	0.25592	1	0.942	0.026848	8	CD44
1.38E-05	0.27994	0.778	0.482	0.027501	8	CRIM1
1.42E-05	0.358368	1	0.948	0.028315	8	ENO1
1.51E-05	0.269429	1	0.842	0.030277	8	EMP1
1.71E-05	0.285303	0.981	0.96	0.034139	8	CALM1
2.03E-05	0.283629	0.963	0.914	0.040593	8	CLIC4
2.09E-05	0.494833	0.852	0.791	0.041887	8	ATF5
4.07E-05	0.31659	0.519	0.122	0.081433	8	KIF23
4.26E-05	0.324033	0.537	0.199	0.085258	8	POSTN
4.35E-05	0.47246	0.889	0.715	0.086945	8	TIMP3
4.88E-05	0.325675	0.648	0.299	0.097523	8	ORC6
4.94E-05	0.274527	0.741	0.454	0.098821	8	ADAMTS1
6.51E-05	0.358301	0.833	0.602	0.130231	8	CYR61
9.66E-05	0.287944	0.704	0.53	0.193157	8	DCBLD2
0.000134	0.387916	0.611	0.341	0.267823	8	CLSPN
0.000298	0.364627	0.963	0.897	0.595621	8	JPT1
0.000308	0.255166	0.815	0.57	0.615982	8	PTGIS
0.000852	0.28185	0.889	0.754	1	8	GAS6
0.000891	0.483236	0.685	0.474	1	8	SERPINE1
0.001165	0.255893	0.852	0.739	1	8	KLF6
0.001306	0.291143	0.87	0.67	1	8	LOXL2
0.002141	0.250669	0.519	0.092	1	8	IL13RA2
0.002283	0.256006	0.926	0.835	1	8	PGK1
0.003933	0.263408	0.963	0.957	1	8	RPL22L1
0.00394	0.350527	1	0.99	1	8	COL1A2
1.57E-22	2.18635	0.919	0.483	3.13E-19	9	CCL5
5.61E-21	0.801155	1	0.999	1.12E-17	9	MT-CO2
1.22E-20	0.859551	1	0.96	2.45E-17	9	PCYOX1
4.70E-20	1.175298	1	0.85	9.39E-17	9	HLA-B
1.56E-19	1.033621	0.973	0.491	3.12E-16	9	SRGN
1.09E-18	1.433499	1	0.99	2.17E-15	9	SAT1
1.78E-18	0.658109	1	1	3.55E-15	9	TMSB10
6.54E-18	0.664074	1	0.986	1.31E-14	9	CD81
1.96E-16	1.020054	1	0.99	3.92E-13	9	SOD2
1.99E-16	0.82881	1	0.951	3.98E-13	9	CD59
3.00E-15	0.797047	1	0.974	6.00E-12	9	SQSTM1
3.82E-15	0.837867	1	0.892	7.64E-12	9	SELENOP
6.88E-15	0.733038	1	0.918	1.38E-11	9	C1QTNF1
2.52E-14	0.659646	1	0.908	5.05E-11	9	CKAP4
4.49E-14	0.506957	0.676	0.114	8.99E-11	9	SNCG
8.32E-14	0.794295	0.946	0.66	1.66E-10	9	HIST1H1C
1.36E-13	0.714103	0.973	0.938	2.72E-10	9	MYL9
2.13E-13	0.501714	1	0.966	4.26E-10	9	APLP2

4.37E-13	0.625133	1	1	8.73E-10	9	FTL
1.04E-12	0.481354	0.946	0.791	2.08E-09	9	STAT1
1.54E-12	0.553167	0.865	0.482	3.07E-09	9	CRIM1
1.87E-12	0.494341	0.946	0.731	3.74E-09	9	HIST1H2AC
2.71E-12	0.61766	1	0.969	5.43E-09	9	THY1
4.08E-12	0.423231	0.649	0.201	8.16E-09	9	PAMR1
5.39E-12	0.523672	0.811	0.531	1.08E-08	9	CHI3L2
2.23E-11	0.656607	0.838	0.412	4.45E-08	9	IL32
8.12E-11	0.449013	1	0.97	1.62E-07	9	ITGB1
8.97E-11	0.527656	0.946	0.718	1.79E-07	9	RDH5
1.19E-10	0.522912	0.73	0.369	2.39E-07	9	SEZ6L2
2.26E-10	0.505725	0.838	0.599	4.51E-07	9	MAP1A
2.32E-10	0.734115	0.838	0.679	4.64E-07	9	FAM213A
2.82E-10	0.486009	0.892	0.762	5.64E-07	9	SLC39A14
3.40E-10	0.476474	1	1	6.79E-07	9	FTH1
7.07E-10	0.460201	0.811	0.633	1.41E-06	9	CD82
8.89E-10	0.563989	0.973	0.867	1.78E-06	9	AKR1B1
9.61E-10	0.346861	0.919	0.715	1.92E-06	9	PAPSS2
1.24E-09	0.537889	0.838	0.752	2.49E-06	9	TFPI
2.05E-09	0.367342	0.865	0.608	4.10E-06	9	APOC1
2.34E-09	0.305501	0.838	0.66	4.67E-06	9	PCOLCE2
1.04E-08	0.319469	0.622	0.308	2.08E-05	9	TMEM106C
1.87E-08	0.718977	0.703	0.318	3.74E-05	9	PTGDS
1.97E-08	0.256695	0.622	0.267	3.94E-05	9	PDGFRL
2.02E-08	1.070846	0.973	0.847	4.04E-05	9	CLU
2.09E-08	0.404671	0.946	0.844	4.17E-05	9	MXRA7
2.63E-08	0.422641	0.568	0.247	5.25E-05	9	DKK3
3.35E-08	0.416646	0.811	0.653	6.70E-05	9	SRPX2
3.75E-08	0.434089	1	0.994	7.51E-05	9	MT-ND3
3.83E-08	0.393342	0.811	0.627	7.66E-05	9	EPDR1
4.03E-08	0.403715	1	0.967	8.07E-05	9	PKM
4.24E-08	0.417217	0.73	0.379	8.47E-05	9	SVIP
4.34E-08	0.385418	0.784	0.638	8.67E-05	9	SVIL
4.59E-08	0.466422	1	0.95	9.18E-05	9	ANXA1
7.13E-08	0.470216	0.892	0.823	0.000143	9	VAT1
1.03E-07	0.463662	0.811	0.738	0.000205	9	QSOX1
1.13E-07	0.657435	0.784	0.407	0.000225	9	IFI27
1.36E-07	0.505924	1	0.964	0.000272	9	CRYAB
1.76E-07	0.43915	0.946	0.94	0.000352	9	STOM
2.58E-07	0.272534	0.811	0.53	0.000516	9	GLS
3.18E-07	0.539222	1	0.914	0.000635	9	HLA-C
4.32E-07	0.332071	0.919	0.775	0.000864	9	CELF2
5.02E-07	0.324136	0.595	0.252	0.001004	9	PPP1R14A
5.57E-07	0.371911	0.757	0.53	0.001114	9	DCBLD2

6.36E-07	0.271423	0.459	0.19	0.001272	9	AC090204.1
6.87E-07	0.610501	0.73	0.508	0.001374	9	LBP
7.13E-07	0.341622	0.919	0.928	0.001425	9	BCAP31
7.68E-07	0.274054	0.432	0.149	0.001535	9	ITGA3
1.02E-06	0.255778	0.459	0.16	0.002039	9	KRT8
1.11E-06	0.286542	0.784	0.542	0.002223	9	LGMN
1.65E-06	0.344156	0.838	0.788	0.003292	9	KCTD12
2.38E-06	0.270338	0.649	0.39	0.004753	9	STEAP2
2.46E-06	0.363427	0.838	0.792	0.004924	9	SORT1
2.72E-06	0.341721	0.838	0.733	0.005438	9	SLC43A2
3.95E-06	0.323142	1	0.956	0.007906	9	METTL7A
4.05E-06	0.381961	0.973	0.969	0.0081	9	TMEM59
4.47E-06	0.381184	0.973	0.836	0.008948	9	IGFBP7
5.58E-06	0.345773	1	0.99	0.011161	9	NEAT1
6.35E-06	0.354461	0.973	0.92	0.012695	9	CTSB
6.63E-06	0.269226	0.73	0.499	0.013256	9	ECHDC2
6.64E-06	0.407431	0.919	0.931	0.013283	9	DHCR24
9.39E-06	0.68453	0.811	0.612	0.01879	9	CYP1B1
1.28E-05	0.251699	0.595	0.354	0.02562	9	LTBP1
1.88E-05	0.361611	0.676	0.5	0.037586	9	PLOD2
2.54E-05	0.346547	0.973	0.94	0.050824	9	COL4A2
2.98E-05	0.853741	0.703	0.568	0.059531	9	SAA2
3.58E-05	0.59122	0.919	0.831	0.071587	9	SAA1
4.06E-05	0.311821	0.919	0.861	0.081176	9	NAMPT
4.47E-05	0.28184	0.919	0.826	0.089486	9	ITGB5
5.03E-05	0.291555	1	0.923	0.100603	9	GLRX
5.18E-05	0.355124	0.892	0.785	0.103642	9	NRP2
5.90E-05	0.276109	0.946	0.754	0.117981	9	GAS6
6.56E-05	0.259583	0.919	0.894	0.13117	9	COL4A1
9.28E-05	0.261343	0.919	0.78	0.18568	9	USP53
9.29E-05	0.505017	0.865	0.848	0.185839	9	CTSK
9.99E-05	0.362057	0.838	0.712	0.199829	9	DEPP1
0.000113	0.309456	0.784	0.621	0.225559	9	BST2
0.000114	0.279464	0.27	0.17	0.22778	9	INMT
0.000144	0.294831	0.703	0.645	0.28748	9	C2CD2
0.000144	0.2936	0.73	0.596	0.288583	9	ITIH5
0.000178	0.334988	0.811	0.645	0.355068	9	ANGPTL4
0.000213	0.442532	0.405	0.246	0.425232	9	SFTA1P
0.000281	0.56568	1	0.999	0.561548	9	GPX3
0.000304	0.253417	0.973	0.978	0.608707	9	LAPTM4A
0.000347	0.283125	0.811	0.854	0.693398	9	BRI3
0.000354	0.291085	0.784	0.7	0.707954	9	SELENBP1
0.000436	0.346093	0.649	0.613	0.871233	9	PPM1K
0.000529	0.262228	0.838	0.708	1	9	ALCAM

0.000626	0.304073	0.973	0.867	1	9	LOX
0.000695	0.28323	0.919	0.847	1	9	REEP5
0.000713	0.270033	1	0.997	1	9	CD63
0.000727	0.406923	0.297	0.146	1	9	LEP
0.00086	0.300649	0.595	0.517	1	9	TMTC1
0.000911	0.323906	0.919	0.894	1	9	RHOBTB3
0.000998	0.33717	0.703	0.656	1	9	KIF22
0.00105	0.262082	0.595	0.44	1	9	DPP4
0.001274	0.324141	0.811	0.775	1	9	C3
0.001328	0.364971	0.73	0.734	1	9	ADAMTS5
0.001597	0.429432	0.703	0.7	1	9	HP
0.001952	0.274576	0.919	0.852	1	9	CARHSP1
0.002234	0.354487	0.622	0.566	1	9	RCAN1
0.002717	0.252814	0.703	0.682	1	9	SEMA3C
0.002839	0.25357	0.946	0.86	1	9	COX7A1
0.003097	0.372023	1	1	1	9	MT1X
0.003966	0.345595	1	0.914	1	9	HLA-A
0.004534	0.321424	1	1	1	9	MT2A
0.006797	0.276379	0.622	0.666	1	9	XG
4.27E-37	0.411956	0.958	0.154	8.55E-34	10	MUM1L1
1.80E-22	0.280495	0.958	0.275	3.61E-19	10	SEMA3G
3.79E-18	0.256527	0.958	0.192	7.57E-15	10	HIST1H1D
1.45E-17	0.533541	1	0.467	2.90E-14	10	AC005224.3
3.24E-17	1.727929	1	0.993	6.49E-14	10	MALAT1
3.89E-17	0.599367	1	0.247	7.78E-14	10	PTGER3
6.70E-17	0.594878	1	0.572	1.34E-13	10	ZNF117
1.38E-16	1.66414	1	0.99	2.77E-13	10	NEAT1
1.36E-15	0.258645	0.958	0.297	2.72E-12	10	PDE4D
1.54E-15	1.113592	1	0.745	3.09E-12	10	PDK4
1.63E-15	0.466571	1	0.617	3.26E-12	10	HMGCR
1.77E-15	0.278299	0.958	0.308	3.54E-12	10	LIPE
2.83E-15	0.316877	0.958	0.431	5.65E-12	10	PDE3B
3.24E-15	1.933427	1	0.812	6.48E-12	10	MT-ND6
4.09E-15	0.560395	0.958	0.452	8.17E-12	10	BTG2
8.38E-15	0.618665	1	0.554	1.68E-11	10	DLAT
3.06E-14	0.340121	1	0.641	6.12E-11	10	LMO3
3.13E-14	1.532914	1	0.954	6.27E-11	10	ACACB
3.60E-14	0.521602	0.958	0.296	7.19E-11	10	DNAJC18
6.32E-14	0.359145	0.958	0.487	1.26E-10	10	RNF150
6.48E-14	0.934368	1	0.722	1.30E-10	10	CSAD
9.38E-14	0.620916	0.958	0.584	1.88E-10	10	AC245060.5
1.36E-13	0.682408	1	0.613	2.72E-10	10	UCP2
2.01E-13	0.505087	0.958	0.121	4.02E-10	10	TENM2
2.15E-13	0.494873	0.958	0.609	4.30E-10	10	ZBED3

2.41E-13	0.42736	1	0.589	4.82E-10	10	HADH
3.91E-13	0.336437	0.958	0.584	7.83E-10	10	TMEM100
4.12E-13	0.776498	0.958	0.136	8.23E-10	10	SPOCD1
4.59E-13	0.327845	0.958	0.275	9.17E-10	10	RAB3IP
4.62E-13	0.41898	1	0.586	9.24E-10	10	PLTP
4.94E-13	0.501235	1	0.75	9.88E-10	10	LDLR
6.93E-13	0.430165	1	0.744	1.39E-09	10	NRCAM
7.81E-13	0.761984	1	0.833	1.56E-09	10	FASN
9.21E-13	0.342347	1	0.686	1.84E-09	10	ADAMTS15
1.76E-12	0.813882	1	0.772	3.53E-09	10	AOC3
2.83E-12	0.333605	0.958	0.465	5.65E-09	10	PHLDA1
3.32E-12	0.712115	1	0.718	6.65E-09	10	RDH5
3.50E-12	0.31126	0.917	0.144	7.00E-09	10	NRK
4.60E-12	0.845711	1	0.867	9.21E-09	10	HEBP2
5.53E-12	0.671938	1	0.94	1.11E-08	10	STOM
1.91E-11	0.257987	0.958	0.51	3.82E-08	10	DUSP6
1.98E-11	0.297671	0.958	0.292	3.96E-08	10	PCSK5
2.83E-11	0.457033	1	0.8	5.65E-08	10	AKR1C2
2.84E-11	0.463383	0.958	0.642	5.68E-08	10	S1PR3
3.83E-11	0.471372	0.958	0.643	7.66E-08	10	CD36
4.35E-11	0.43994	0.958	0.619	8.71E-08	10	GPD1L
4.45E-11	0.824207	1	0.706	8.89E-08	10	CTNNB1
7.71E-11	0.442091	0.958	0.36	1.54E-07	10	SLC20A1
9.47E-11	0.630614	1	0.814	1.89E-07	10	DUSP1
1.65E-10	0.364696	1	0.796	3.29E-07	10	VKORC1L1
1.67E-10	0.44393	1	0.786	3.35E-07	10	CS
1.95E-10	0.471566	0.958	0.339	3.89E-07	10	RRP12
2.47E-10	0.508117	1	0.951	4.93E-07	10	ACSL1
2.50E-10	0.327382	0.958	0.451	5.01E-07	10	PHKG1
2.57E-10	0.471367	1	0.807	5.15E-07	10	RASD1
3.02E-10	0.398919	0.958	0.71	6.05E-07	10	ACOX1
3.20E-10	0.27891	0.958	0.648	6.39E-07	10	FNDC4
3.21E-10	0.339156	0.958	0.601	6.42E-07	10	TOB1
3.29E-10	0.346038	1	0.85	6.59E-07	10	PIK3R1
3.52E-10	0.286233	0.958	0.651	7.05E-07	10	ITGA7
4.29E-10	0.470015	1	0.821	8.57E-07	10	PLIN4
4.57E-10	0.588645	0.958	0.707	9.14E-07	10	LPL
4.92E-10	0.258595	0.958	0.643	9.84E-07	10	NR1H3
5.63E-10	0.408366	1	0.874	1.13E-06	10	GPC1
7.20E-10	0.296949	0.125	0.425	1.44E-06	10	ITGA10
8.69E-10	0.305936	1	0.498	1.74E-06	10	CXXC5
1.05E-09	0.397091	0.958	0.356	2.11E-06	10	NAV3
1.14E-09	0.318193	1	0.826	2.28E-06	10	GPAM
1.53E-09	0.48036	1	0.768	3.05E-06	10	MSMO1

1.62E-09	0.47976	0.958	0.597	3.23E-06	10	ELOVL6
1.75E-09	0.346579	0.958	0.699	3.50E-06	10	HP
1.82E-09	0.495673	0.958	0.541	3.64E-06	10	TENM3
1.86E-09	0.320497	0.958	0.679	3.71E-06	10	FAM213A
2.30E-09	0.523167	0.958	0.305	4.61E-06	10	PLAUR
2.40E-09	0.430792	0.958	0.731	4.81E-06	10	PLIN1
2.70E-09	0.372134	0.958	0.663	5.40E-06	10	ACSS2
2.82E-09	0.432845	0.958	0.754	5.64E-06	10	RHOB
3.43E-09	0.358297	0.958	0.699	6.86E-06	10	AACS
4.83E-09	0.302653	1	0.854	9.67E-06	10	FABP5
5.46E-09	1.148654	1	0.677	1.09E-05	10	PCDH9
5.60E-09	0.522896	0.958	0.887	1.12E-05	10	APCDD1
6.03E-09	1.154075	0.917	0.779	1.21E-05	10	MEG3
6.21E-09	0.391893	1	0.788	1.24E-05	10	IGF1
8.44E-09	0.514545	0.958	0.857	1.69E-05	10	PNPLA2
8.47E-09	0.296249	1	0.788	1.69E-05	10	KCTD12
1.19E-08	0.260555	0.958	0.497	2.38E-05	10	TNFRSF21
1.34E-08	0.273294	1	0.894	2.68E-05	10	SCD
1.34E-08	0.534113	0.958	0.69	2.68E-05	10	MRAS
1.38E-08	1.033558	1	0.721	2.77E-05	10	ADAM12
1.58E-08	0.465105	1	0.801	3.16E-05	10	AKAP12
2.52E-08	0.273949	0.167	0.391	5.04E-05	10	ARL4A
2.63E-08	0.255198	0.958	0.604	5.26E-05	10	GPC4
3.18E-08	0.596004	0.958	0.806	6.37E-05	10	FADS3
3.26E-08	0.276739	0.958	0.597	6.51E-05	10	PPIF
3.95E-08	0.391413	0.958	0.674	7.91E-05	10	SESN2
4.14E-08	0.542686	0.958	0.72	8.27E-05	10	CCND2
4.99E-08	0.727441	1	0.945	9.98E-05	10	LAMA4
5.83E-08	0.417691	1	0.866	0.000117	10	MKNK2
7.96E-08	0.463239	1	0.948	0.000159	10	IGFBP5
8.04E-08	0.301721	0.958	0.318	0.000161	10	SMTN
8.66E-08	0.36266	1	0.698	0.000173	10	ERP44
1.52E-07	0.714129	1	0.94	0.000305	10	11-Sep
1.94E-07	0.41327	0.958	0.341	0.000388	10	DNAJB4
2.15E-07	0.403682	0.958	0.727	0.00043	10	MDM2
2.30E-07	0.670995	0.917	0.39	0.000459	10	STEAP2
2.66E-07	0.620611	0.958	0.39	0.000533	10	PPME1
3.50E-07	0.264292	1	0.956	0.000701	10	METTL7A
6.76E-07	0.380412	0.958	0.329	0.001352	10	RGMB
9.06E-07	1.055662	0.875	0.713	0.001811	10	KCNQ1OT1
9.33E-07	0.371661	0.958	0.725	0.001865	10	RETSAT
1.34E-06	0.380851	0.792	0.196	0.002689	10	ALPK2
1.71E-06	0.272747	0.958	0.817	0.00341	10	ZNF703
2.12E-06	0.307049	0.958	0.432	0.004246	10	SMC4

2.22E-06	0.767646	0.958	0.717	0.004432	10	TSHZ2
2.46E-06	0.561496	1	0.905	0.004928	10	MMP14
2.69E-06	0.252467	0.958	0.702	0.005377	10	MAFB
3.46E-06	0.382223	0.958	0.499	0.006923	10	ECHDC2
5.79E-06	0.286405	1	0.96	0.011586	10	PCYOX1
6.10E-06	0.281711	0.958	0.744	0.012208	10	C19orf12
6.65E-06	0.310533	0.208	0.44	0.013292	10	RUNX1
9.14E-06	0.332632	1	0.83	0.018289	10	NOP56
1.80E-05	0.261066	0.958	0.541	0.035917	10	ANGPT1
2.13E-05	0.997099	1	0.99	0.042687	10	SAT1
2.55E-05	0.737655	1	0.933	0.05092	10	DDIT4
2.59E-05	1.709104	1	0.999	0.051702	10	MT-CO2
2.91E-05	1.251075	0.792	0.82	0.058144	10	NABP1
2.98E-05	0.277911	0.792	0.226	0.059611	10	ARRDC4
3.84E-05	0.336239	0.958	0.734	0.076891	10	CUL4B
7.21E-05	0.2659	0.958	0.609	0.144294	10	ABCA1
9.94E-05	0.966558	1	0.917	0.198846	10	REV3L
0.000108	0.892555	0.958	0.739	0.215624	10	ERV3-1
0.000136	0.42149	1	0.991	0.271399	10	SRPX
0.000177	0.365314	0.958	0.517	0.353878	10	BDH2
0.000219	0.356725	1	0.964	0.437072	10	CFD
0.000286	0.569984	0.958	0.407	0.571334	10	RDH10
0.000385	0.966966	1	0.645	0.770037	10	SOX4
0.000433	0.414901	1	0.774	0.866887	10	TUBB6
0.000446	1.27882	1	0.994	0.891112	10	MT-ND3
0.000467	0.53209	1	0.946	0.934428	10	SHC1
0.000526	0.320394	0.25	0.335	1	10	SLC2A3
0.000889	0.261075	0.708	0.223	1	10	BDKRB2
0.00093	0.473965	0.958	0.983	1	10	CALD1
0.001027	0.276364	0.958	0.682	1	10	SEMA3C
0.001097	0.866996	1	0.803	1	10	CDKN1A
0.001957	0.390753	0.208	0.259	1	10	LIMS2
0.002134	0.572427	0.25	0.456	1	10	ADAMTS1
0.002532	0.600823	0.958	0.623	1	10	KCTD20
0.003336	1.14799	0.667	0.347	1	10	SYNPO2
0.003368	0.27655	0.958	0.665	1	10	ALDH1A3
0.004444	0.490953	0.292	0.232	1	10	IFI44L
0.004453	0.740011	0.667	0.321	1	10	PAWR
0.004972	0.254499	0.292	0.53	1	10	EMX2
0.005857	0.304517	0.958	0.507	1	10	TP53I3
0.006351	0.39107	0.25	0.452	1	10	MYO1B

Table S2 the number of label genes, annotations and cell numbers of different subgroups in UMAP clustering results

category	number of label genes	New ID	GM-cell number	AD-cell number
0	2	0_Osteoclast_2852 cells	2403	449
1	72	1_Astrocyte_2007 cells	1	2006
2	70	2_Unrestricted somatic stem cell_1269 cells	1075	194
3	21	3_Liver bud hepatic cell_1147 cells	10	1137
4	150	4_DCLK1+ progenitor cell_834 cells	301	533
5	99	5_Trophoblast cell_380 cells	374	6
6	58	6_Migration phase fetal germ cell_322 cells	176	146
7	182	7_Astrocyte_123 cells	123	0
8	174	8_ 8_Neural progenitor cell_54 cells	44	10
9	125	9_Fibroblast_37 cells	9	28
10	154	10_Microglial cell_24 cells	22	2

Table S3 cluster marker number

cluster ID	0	1	2	3	4	5	6	7	8	9	10
marker number	2	72	70	21	150	99	58	182	174	125	154

Table S4 combine annotation cluster cells

Cluster ID	Cell Name	Marker Number	Signature Number	Hit Number	Score	p value	Hit genes
0	Osteoclast	6	2	1	0.849156	0.000653	CTSK
0	FGFR1HighNME5-epithelial cell	189	2	1	0.075649	0.017654	CTSK
1	Astrocyte	425	72	11	0.937158	2.09E-07	DBI,ELOVL5,FABP5,FADS2,GLUL,HMGCS1,ITGA7,LPL,RHOB,SCD,SRPX
1	PROCR+ progenitor cell	21	72	2	0.763763	0.002674	IGFBP5,RAMP2
1	Lake et al.Science.In3	11	72	1	0.729657	0.039464	IGFBP5
1	Migration phase fetal germ cell	147	72	4	0.709316	0.001661	DBI,FABP5,LAMA4,SAT1
1	Primitive endoderm cell	87	72	4	0.701162	0.000248	COL4A1,COL4A2,IGF1,ITIH5
1	Microglial cell	433	72	8	0.698748	0.000112	FABP5,GLUL,GSN,IGF1,PDK4,RHOB,SAT1,UCP2
1	Leydig precursor cell	216	72	6	0.687218	0.0001	ALDH1A3,HSPB6,IGF1,IGFBP5,INSIG1,SRPX
1	Liver bud hepatic cell	191	72	5	0.655559	0.00051	ACSL1,AKR1C1,AKR1C2,ELOVL5,RHOB
1	Epiblast cell	79	72	3	0.64355	0.002639	RASD1,SAT1,SH3PXD2A
1	Basal epithelial cell	6	72	1	0.604207	0.023211	MYLK
1	CD1C-CD141-dendritic cell	284	72	5	0.551853	0.002846	CFD,GLUL,INSIG1,SAT1,TSPAN14
1	Glutamatergic neuron	7	72	1	0.551828	0.026483	GLUL
1	Acinar cell	73	72	3	0.547753	0.002125	AKR1C1,AKR1C2,CPB1
1	Gonadal mitotic phase fetal germ cell	275	72	5	0.541514	0.002484	COL4A1,COL4A2,FABP3,RASD1,SH3PXD2A
1	Alpha cell	92	72	3	0.533797	0.003998	FADS2,PLA2G16,SORT1
1	Cancer cell	46	72	2	0.524893	0.011294	FASN,GLUL
1	Fat cell (adipocyte)	22	72	1	0.52021	0.074288	FABP4
1	MKI67+ progenitor cell	105	72	3	0.513323	0.005718	FABP4,FZD4,RAMP2
1	Neutrophil	90	72	2	0.468017	0.038151	G0S2,SAT1
1	Gonadal endothelial	455	72	6	0.4659	0.0044	AKAP12,FZD4,LAMA4,PDK4,RAMP2,SHC1

	cell				94	57	
1	Ionocyte cell	278	72	4	0.461815	0.014847	ACSL1,IGF1,IGFBP5,TSPAN14
1	Oligodendrocyte	137	72	3	0.461353	0.011619	DHCR24,GSN,SCD
1	Leydig cell	373	72	4	0.409046	0.037716	CYB5A,HSPB6,LAMA4,PNPLA2
1	Mesenchymal cell	66	72	2	0.356965	0.021852	AKAP12,HSPB6
1	Endothelial cell	367	72	4	0.356001	0.035887	AKAP12,FABP5,LAMA4,RAMP2
1	Trophectoderm cell	89	72	2	0.345559	0.037401	FABP3,FASN
1	Enteroendocrine cell	40	72	1	0.328877	0.128606	APCDD1
1	Sertoli cell	436	72	4	0.324703	0.060113	HK2,PLA2G16,PRDX6,SAT1
1	Basal cell	86	72	1	0.258799	0.253548	FABP5
1	AXL+SIGLEC6+ dendritic cell	73	72	1	0.252809	0.220149	PLA2G16
1	Plasmacytoid dendritic cell	308	72	2	0.250713	0.278379	PLA2G16,RASD1
1	Cardiomyocyte	24	72	1	0.236784	0.080484	FABP3
1	Ciliated cell	249	72	2	0.226873	0.205404	PLA2G16,UCP2
1	Epithelial cell	61	72	1	0.222784	0.188014	FZD4
1	Oligodendrocyte progenitor cell	99	72	1	0.221108	0.285535	DBI
1	Retinoid acid signaling-responsive fetal germ cell	205	72	2	0.219307	0.152744	FAM213A,SLC25A1
1	Mucosal-associated invariant T cell	58	72	1	0.217969	0.179779	ADAM12
1	Enterocyte	44	72	1	0.192967	0.140243	MLXIPL
1	FOXN4+ cell	77	72	1	0.191454	0.23058	INSIG1
1	DCLK1+ progenitor cell	98	72	1	0.189909	0.283124	SRPX
1	CD141+CLEC9A+ dendritic cell	96	72	1	0.183712	0.278276	ELOVL5
1	Granulosa cell	182	72	1	0.1779	0.46016	FABP5
1	Goblet cell	64	72	1	0.175	0.196168	GSN
1	CD4+ cytotoxic T cell	72	72	1	0.172063	0.21752	GLUL
1	Effector CD8+	73	72	1	0.1708	0.2201	GLUL

	memory T (Tem) cell				8	49	
1	Secretory cell	60	72	1	0.167829	0.185278	ALDH1A3
1	FGFR1LowNME5- epithelial cell	49	72	1	0.165714	0.154574	FABP3
1	Regulatory T (Treg) cell	436	72	2	0.16283	0.659513	SAT1,UCP2
1	Mast cell	101	72	1	0.161196	0.290335	PRDX6
1	Cancer stem cell	149	72	1	0.15975	0.396448	ALDH1A3
1	Enterocyte progenitor cell	110	72	1	0.156368	0.311543	G0S2
1	Morula cell (Blastomere)	96	72	1	0.134722	0.278276	AKAP12
1	Neural progenitor cell	172	72	1	0.108274	0.441588	UCP2
1	Ciliated epithelial cell	374	72	1	0.107554	1	APCDD1
1	FGFR1HighNME5- epithelial cell	189	72	1	0.10038	0.472797	CSAD
1	Paneth cell	271	72	1	0.086259	0.600772	UCP2
2	Unrestricted somatic stem cell	7	70	1	1.349333	0.025758	ALCAM
2	Mesenchymal cell	66	70	6	1.336774	1.26E-07	ALCAM,CCL2,COL1A2,FN1,ID3,IGFBP7
2	Myoblast	7	70	2	1.27752	0.000371	ID1,ID3
2	Mesenchymal progenitor cell	8	70	1	1.262186	0.028932	ALCAM
2	Limbal epithelial stem cell	4	70	1	1.19	0.016176	ALCAM
2	Astrocyte	425	70	13	1.15641	1.44E-09	ALCAM,DDAH1,ELN,ID1,ID2,IGFBP7,MT2A,PLEC,PSAT1,SERPINE2,TIMP3,TMSB10,VEPH1
2	Intestinal stem cell	10	70	1	1.128933	0.035248	ALCAM
2	Osteocyte	8	70	1	1.1243	0.028932	FN1
2	Oligodendrocyte progenitor cell	99	70	5	0.976897	2.27E-05	ALCAM,PSAT1,SERPINE2,STMN2,TMSB10
2	Mesangial cell	5	70	1	0.948093	0.019381	FN1
2	Myofibroblast	5	70	1	0.948093	0.019381	FN1
2	Primitive endoderm cell	87	70	5	0.930594	1.25E-05	ANKRD1,FN1,ID2,MARCKS,SERPINE2
2	Cancer stem-like cell	7	70	1	0.899555	0.025758	ALCAM

2	PROM1Low progenitor cell	7	70	1	0.899555	0.025758	ALCAM
2	Bone marrow stem cell	8	70	1	0.841457	0.028932	ALCAM
2	DCLK1+ progenitor cell	98	70	5	0.826305	2.16E-05	CCL2,COL1A1,COL1A2,COL8A1,LOX
2	Mesenchymal stem cell	83	70	2	0.755178	0.031367	ALCAM,FN1
2	Gonadal endothelial cell	455	70	9	0.713525	1.91E-05	ACTB,ADM,CTGF,CYR61,FN1,ID1, ID3,MARCKS,TIMP3
2	Leydig precursor cell	216	70	6	0.704909	8.57E-05	COL1A1,ELN,FBN2,SERPINE2, TIMP3,TPM1
2	Cardiac progenitor cell	17	70	1	0.577235	0.057038	ALCAM
2	CD1C-CD141- dendritic cell	284	70	6	0.576776	0.000364	CDC42EP3,FLNA,HEG1,MARCKS, MT2A,TAGLN
2	T helper1 (Th1) cell	18	70	1	0.572756	0.060111	STAT1
2	Stem cell	70	70	1	0.568929	0.207006	ALCAM
2	Lake et al.Science.Ex1	14	70	1	0.561249	0.047759	SERPINE2
2	Oviduct-derived stem cell	18	70	1	0.560971	0.060111	ALCAM
2	Pericyte	19	70	1	0.546009	0.063174	ALCAM
2	Lake et al.Science.In3	11	70	1	0.53066	0.038391	CRIM1
2	Ionocyte cell	278	70	5	0.522991	0.002299	ADM,ID3,THBS1,TPM1,USP53
2	Adipose-derived stromal cell	21	70	1	0.519359	0.06927	ALCAM
2	Osteoblast	22	70	1	0.507418	0.072304	ALCAM
2	Endometrial stem cell	23	70	1	0.496264	0.075328	ALCAM
2	Circulating fetal cell	39	70	2	0.489992	0.007896	ACTB,COL1A2
2	Cardiomyocyte	24	70	1	0.485815	0.078342	ALCAM
2	Progenitor cell	25	70	1	0.476	0.081347	ALCAM
2	Idiopathic pulmonary fibrosis cell	13	70	1	0.471495	0.044646	COL1A1
2	Granulosa cell	182	70	4	0.469952	0.003189	COL1A1,COL1A2,FN1,MT2A
2	Mesenchymal stromal cell	58	70	1	0.468764	0.175258	ALCAM
2	Leydig cell	373	70	5	0.467038	0.007784	ALCAM,PRSS23,PSAT1,STAT1,TGFBI

2	Endothelial cell	367	70	5	0.463532	0.00729	ALCAM,DPYSL2,FN1,MARCKS,TIMP3
2	Liver bud hepatic cell	191	70	4	0.454405	0.003774	MT2A,SERPINE1,SLC38A2,TGFBI
2	AXL+SIGLEC6+ dendritic cell	73	70	3	0.440075	0.001961	ATF5,DPYSL2,PPP1R14A
2	Pit progenitor cell	52	70	2	0.438213	0.01341	PPP1R14A,TMEM47
2	Oligodendrocyte	137	70	3	0.434014	0.010767	ACTG1,PPP1R14A,TMSB10
2	MKI67+ progenitor cell	105	70	2	0.431348	0.047614	HEG1,SERPINE2
2	Neuron	75	70	1	0.412228	0.219876	ALCAM
2	Macrophage	74	70	2	0.410354	0.025512	CCL2,TGFBI
2	M1 macrophage	16	70	1	0.405	0.053955	STAT1
2	Cancer stem cell	149	70	1	0.389954	0.387937	ALCAM
2	Tuft progenitor cell	10	70	1	0.385798	0.035248	CCL2
2	Stromal cell	42	70	1	0.367242	0.130971	ALCAM
2	Effector CD8+ memory T (Tem) cell	73	70	2	0.365168	0.024892	FLNA,PRSS23
2	Plasmacytoid dendritic cell	308	70	4	0.362395	0.018867	CRIM1,IGFBP3,SLC38A2,TGFBI
2	Enteroendocrine cell	40	70	1	0.354175	0.125273	LOX
2	Smooth muscle cell	11	70	1	0.325632	0.038391	TAGLN
2	M2 macrophage	15	70	1	0.315003	0.050862	CCL2
2	Retinoid acid signaling-responsive fetal germ cell	205	70	2	0.3115	0.146089	FBN2,SERPINE2
2	Fibroblast	31	70	1	0.305329	0.099172	COL1A1
2	Epithelial cell	61	70	1	0.304728	0.183311	ALCAM
2	Pancreatic polypeptide cell	15	70	1	0.299511	0.050862	ID2
2	Migration phase fetal germ cell	147	70	2	0.290325	0.084946	COL1A2,TMSB10
2	Exhausted CD8+ T cell	242	70	3	0.280271	0.045829	ID2,ID3,PTGIS
2	Gonadal mitotic phase fetal germ cell	275	70	3	0.27739	0.062233	FBLN5,FLNA,PRSS23

2	Acinar cell	73	70	1	0.252809	0.214753	PSAT1
2	Beta cell	97	70	1	0.241652	0.274101	ALCAM
2	Ciliated epithelial cell	374	70	2	0.202698	0.346967	CTGF,ITGBL1
2	Ciliated cell	249	70	2	0.186315	0.196925	CTGF,IGFBP7
2	Naive CD8+ T cell	88	70	1	0.183353	0.252383	LMO7
2	Secretory cell	60	70	1	0.170411	0.180635	PRSS23
2	Regulatory T (Treg) cell	436	70	2	0.166662	0.65506	DPYSL2,SERPINE2
2	Paneth cell	271	70	2	0.157939	0.223125	ATF5,IRS2
2	CD4+ cytotoxic T cell	72	70	1	0.155563	0.212179	PRSS23
2	Microglial cell	433	70	2	0.152821	0.654033	CCL2,NABP1
2	Trophectoderm cell	89	70	1	0.15052	0.254827	PALLD
2	Natural killer cell	60	70	1	0.149755	0.180635	ID2
2	Neural stem cell	60	70	1	0.149755	0.180635	ID2
2	Alpha cell	92	70	1	0.148045	0.262113	PALLD
2	Neutrophil	90	70	1	0.143357	0.257264	ADM
2	Brush cell (Tuft cell)	76	70	1	0.133061	0.222425	ID2
2	Neuroendocrine cell	78	70	1	0.131344	0.227499	ID2
2	Secretory progenitor cell	115	70	1	0.126821	0.315689	ADM
2	Granulocyte-monocyte progenitor	104	70	1	0.125514	0.290562	IGFBP7
2	CD141+CLEC9A+ dendritic cell	96	70	1	0.118392	0.271719	ID2
2	Mast cell	101	70	1	0.113434	0.283553	TIMP3
2	Sertoli cell	436	70	1	0.093867	1	NABP1
2	Neural progenitor cell	172	70	1	0.080824	0.432492	STMN2
2	B cell	260	70	1	0.080623	0.575325	ID3
3	Liver bud hepatic cell	191	21	9	0.843688	1.19E-13	MT1A,MT1E,MT1F,MT1G,MT1M,MT1X,MT2A,SAA1,SAA2
3	Granulosa cell	182	21	6	0.5752	2.04E-	GPX3,MT1A,MT1E,MT1F,MT2A,TSC22D3

					09	08	
3	Astrocyte	425	21	6	0.415221	2.67E-06	ELN,GPX3,MT1E,MT1X,MT2A,SPOCK1
3	Lymphocyte	8	21	1	0.367696	0.008779	IL1RL1
3	Basal cell	86	21	2	0.273895	0.003322	IGFBP6,MT1X
3	Nephron epithelial cell	17	21	1	0.261938	0.017484	SAA2
3	PROCR+ progenitor cell	21	21	1	0.261861	0.02133	MGP
3	Mast cell	101	21	2	0.236819	0.004519	IL1RL1,MT1F
3	Leydig precursor cell	216	21	3	0.228619	0.001219	ELN,IGF1,TSC22D3
3	T helper2 (Th2) cell	21	21	1	0.226947	0.02133	IL1RL1
3	Germ cell	27	21	1	0.203997	0.027071	SCARA5
3	Goblet cell	64	21	1	0.1725	0.061775	MT1M
3	Lymphoid cell	37	21	1	0.170975	0.036569	IL1RL1
3	Epiblast cell	79	21	1	0.157512	0.075506	MT1X
3	CD1C-CD141-dendritic cell	284	21	2	0.153095	0.031491	MT2A,TSC22D3
3	Megakaryocyte erythroid cell	56	21	1	0.138976	0.054373	IL1RL1
3	Myeloid cell	57	21	1	0.137751	0.055301	IL1RL1
3	Natural killer cell	60	21	1	0.134263	0.058081	IL1RL1
3	T cell	69	21	1	0.125201	0.066374	IL1RL1
3	DCLK1+ progenitor cell	98	21	1	0.121218	0.092623	MGP
3	Primitive endoderm cell	87	21	1	0.109355	0.08275	IGF1
3	Microglial cell	433	21	2	0.107648	0.066882	IGF1,TSC22D3
3	Gonadal mitotic phase fetal germ cell	275	21	1	0.084423	0.238225	MT1X
3	FGFR1HighNME5-epithelial cell	189	21	1	0.077104	0.170506	SCARA5
3	Ciliated cell	249	21	1	0.068442	0.218328	SAA2
3	Ionocyte cell	278	21	1	0.061176	0.240489	IGF1
3	Leydig cell	373	21	1	0.0610	0.3089	SLC39A8

					98	72	
4	DCLK1+ progenitor cell	98	150	15	3.820397	2.12E-15	C1R,C3,CCDC80,DCN,DPT,FBLN1, LUM,MGP,MMP2,NNMT,PCOLCE, RCN3,SERPINF1,SERPING1,SRPX
4	Migration phase fetal germ cell	147	150	16	3.447606	3.05E-14	DBI,ETFB,FABP5,FKBP11,GSTP1, IFITM1,MGST1,MGST3,MYL6,NQO1, OLFML3,PLTP,PSMB3,PSMB6,TPM2, TXN
4	Astrocyte	425	150	22	2.646064	6.26E-13	ANXA5,CCDC80,CD63,CLU, CRYAB,CTSL,DBI,DCN,EFEMP1, FABP5,HLA-A,HLA-B,HLA-C, HSPB1,LGALS3,LGALS3BP, PHGDH,PLTP,SLC3A2,SRPX, TIMP1,UCHL1
4	Liver bud hepatic cell	191	150	15	2.567243	1.57E-11	AKR1C1,AKR1C2,C1R,EMP2, EMP3,HP,HSPB1,NME1,PSME2, RARRES2,S100A6,SAA1, SERPINF1,TIMP1,TUBA1B
4	Microglial cell	433	150	19	2.255792	3.86E-10	ANXA5,APLP2,BLVRB,C3,CD63, CTSL,FABP5,FBLN1,GSN,HLA-B, HLA-C, MYL12A, OLFML3, PLTP, PRDX2,RCN3,S100A11,SERPINF1, TUBA1A
4	Regulatory T (Treg) cell	436	150	16	2.154151	1.11E-07	APLP2,ECH1,ENO1,GSTO1,HSPB1, LAPTM4A,LGALS3,NDUFC2,PGK1, PKM,PLTP,PRDX1,PSMB1,PSMB3, PSMB6,SLC3A2
4	PROCR+ progenitor cell	21	150	4	1.998876	2.53E-05	CD248,MGP,PCOLCE,RCN3
4	Basal cell	86	150	10	1.984123	1.32E-09	CAV1,FABP5,FBLN1,GPNMB, HSPB1,IGFBP6,LGALS3,S100A10, S100A6,TIMP1
4	Sertoli cell	436	150	15	1.917568	6.22E-07	CLU,ECH1,MGST3,PHGDH,PLA2G16, PRDX1,PRDX4,PRDX6,S100A10, SERPINF1,SLC3A2,TECR,TUBA1A, TUBB4B,UCHL1
4	Macrophage	74	150	5	1.861125	0.000226	CD63,GPNMB,LGALS3,PLTP,S100A11
4	Acinar cell	73	150	7	1.825842	1.42E-06	AKR1C1,AKR1C2,AKR1C3,MGST1,PLTP, PTGR1,RARRES2
4	Gonadal mitotic phase fetal germ cell	275	150	11	1.737911	5.34E-06	CCPG1,CD63,CTSL,EFEMP1,EMC7, FBLN1,HSPA5,MGST3,MMP2,PSME2, SLC3A2
4	Basophil	27	150	1	1.524205	0.177532	CD63
4	Leydig precursor cell	216	150	11	1.521405	5.67E-07	C1R,CALD1,DCN,EMP2,MMP2, OLFML3,PLIN2,RARRES2,RCN3, SRPX,TPM2
4	Plasmacytoid dendritic cell	308	150	10	1.471231	7.96E-05	DHRS7,HSP90B1,LMAN1,NOP56, NUCB2,PLA2G16,SERPINF1,SLC3A2, TPM2,TXN

4	Liver stem cell	7	150	1	1.451384	0.054286	GSTP1
4	Granulocyte-monocyte progenitor	104	150	5	1.447337	0.000993	ANXA1,APLP2,MGST1,NQO1,NUCB2
4	Ciliated cell	249	150	8	1.434752	0.000446	CALM1,HSP90AA1,NQO1,PLA2G16,PRDX1,SSB,TUBA1A,TUBB4B
4	Endothelial cell	367	150	10	1.381201	0.000315	ANXA2,CD248,CLEC2B,CTSL,FABP5,HSPB1,PCOLCE,PSMB3,S100A4,SERPING1
4	CD1C-CD141-dendritic cell	284	150	10	1.300713	4.13E-05	CCPG1,CFD,IFITM1,LGALS3,PLIN2,S100A11,S100A4,TAGLN,TIMP1,TUBA1A
4	Oligodendrocyte progenitor cell	99	150	5	1.280418	0.000804	ANXA5,APOD,CD63,DBI,MMP2
4	Secretory cell	60	150	4	1.229027	0.001031	C3,HLA-A,IFITM1,TMEM45A
4	Parietal progenitor cell	6	150	1	1.224745	0.047664	ANXA1
4	Granulosa cell	182	150	8	1.214166	5.65E-05	C1R,EMP3,FABP5,FBLN1,IFITM1,MMP2,PGK1,TMEM45A
4	Leydig cell	373	150	10	1.184681	0.000357	CYB5A,EPHX1,ETFB,NME1,PGK1,PLTP,S100A4,S100A6,STEAP1,TXN
4	Granulocyte	12	150	1	1.143154	0.086718	CD63
4	Exhausted CD8+ T cell	242	150	6	1.100515	0.007859	ANXA5,CD63,GSTO1,PKM,PLPP1,PSME2
4	Morula cell (Blastomere)	96	150	4	1.077775	0.00525	AIMP1,CCT5,S100A11,TXN
4	Osteoclast	6	150	1	1.061446	0.047664	CTSK
4	Smooth muscle cell	11	150	2	1.031169	0.003561	CALD1,TAGLN
4	Oligodendrocyte	137	150	5	0.98422	0.003173	ACAT2,APOD,FDPS,GSN,NME1
4	Stromal cell	42	150	3	0.969025	0.003773	LUM,MMP2,TIMP1
4	CD1C+_B dendritic cell	45	150	2	0.963	0.042326	ANXA1,MGST1
4	Lake et al.Science.Ex4	10	150	1	0.948683	0.07388	ANXA1
4	Mast cell	101	150	3	0.897524	0.036039	CLU,LAPTM4A,PRDX6
4	FGFR1HighNME5-epithelial cell	189	150	6	0.888874	0.00246	CTSK,LUM,MFAP5,MMP2,PCOLCE,RARRES2
4	Mesenchymal stromal cell	58	150	2	0.882379	0.065405	CD63,SLC3A2
4	Mesenchymal stem cell	83	150	2	0.853966	0.118168	CD63,SLC3A2
4	Lymphocyte	8	150	1	0.841457	0.060863	ANXA5
4	Gonadal endothelial cell	455	150	7	0.820413	0.042896	ACTB,ANXA2,CALM1,CCPG1,HSPB1,OLFML3,S100A11
4	CD4+ cytotoxic T	72	150	2	0.8013	0.0938	AKR1C3,GSTP1

	cell				88	21	
4	Neural progenitor cell	172	150	5	0.762493	0.00799	HSPB1,PHGDH,PLIN2,TUBA1B,TUBB4B
4	Neutrophil	90	150	2	0.702026	0.134392	G0S2,S100A11
4	Mesenchymal cell	66	150	3	0.647461	0.012345	MMP2,PLTP,S100A4
4	Platelet	38	150	1	0.642397	0.238372	CD63
4	AXL+SIGLEC6+ dendritic cell	73	150	3	0.625	0.015994	PLA2G16,S100A10,TXN
4	Fat cell (adipocyte)	22	150	2	0.592698	0.011985	FABP4,PLIN2
4	Purkinje cell	23	150	1	0.588011	0.154231	CLU
4	Exhausted CD4+ T cell	102	150	2	0.564384	0.16324	PKM,PSME2
4	Dendritic cell	31	150	1	0.506487	0.200194	CLU
4	Ionocyte cell	278	150	4	0.496601	0.133924	AKR1B1,APLP2,DHRS7,RARRES2
4	Tuft progenitor cell	10	150	1	0.480666	0.07388	TPM2
4	LGR5+ stem cell	52	150	1	0.479816	0.30938	MGST1
4	Stem cell	70	150	1	0.458968	0.391093	GSTP1
4	Primordial germ cell	18	150	1	0.438406	0.124181	IFITM1
4	Enterocyte progenitor cell	110	150	2	0.427151	0.183034	G0S2,MGST1
4	Paneth cell	271	150	3	0.421575	0.44336	CLEC2B,CTSL,PLIN2
4	Spermatogonial stem cell	9	150	1	0.406667	0.067394	UCHL1
4	Alpha cell	92	150	2	0.406603	0.139117	CLU,PLA2G16
4	Plasma cell	37	150	1	0.401134	0.233031	FKBP11
4	Trophectoderm cell	89	150	2	0.368879	0.132044	EMP2,S100A6
4	Effector CD8+ memory T (Tem) cell	73	150	1	0.353464	0.403745	EMP3
4	Epiblast cell	79	150	2	0.353278	0.109138	CCDC80,IFITM1
4	Enterocyte	44	150	1	0.349753	0.269651	STEAP1
4	Brush cell (Tuft cell)	76	150	1	0.344124	0.416136	ANXA1
4	Neuroendocrine	78	150	1	0.3396	0.4242	ANXA1

	cell				83	55	
4	Neuron	75	150	1	0.327935	0.412035	DCN
4	Mucosal-associated invariant T cell	58	150	1	0.320388	0.337761	FKBP11
4	FOXN4+ cell	77	150	1	0.312252	0.42021	ENO1
4	Enteroendocrine cell	40	150	1	0.309903	0.248943	DPT
4	MKI67+ progenitor cell	105	150	2	0.306433	0.170618	CD248,FABP4
4	Oocyte	86	150	1	0.280365	0.455622	REXO2
4	Goblet cell	64	150	1	0.2775	0.364983	GSN
4	Cancer stem cell	149	150	1	0.270347	1	HSPA5
4	Circulating fetal cell	39	150	1	0.2498	0.243676	ACTB
4	Beta cell	97	150	1	0.249775	0.495998	APLP2
4	B cell	260	150	1	0.245589	1	CD63
4	Naive CD8+ T cell	88	150	1	0.221729	0.463195	CD248
4	T cell	69	150	1	0.146871	0.386817	UCHL1
4	Retinoid acid signaling-responsive fetal germ cell	205	150	1	0.118733	1	NEXN
5	Trophoblast cell	4	99	1	2.7	0.022786	KRT7
5	Osteocyte	8	99	2	2.259206	0.00092	BGN,FN1
5	Liver progenitor cell	5	99	1	1.811215	0.027281	KRT7
5	DCLK1+ progenitor cell	98	99	9	1.743523	1.57E-09	COL1A1,COL1A2,COL8A1,CSF1,HTRA1,LOX,MYC,RCN3,THBS2
5	Progenitor cell	25	99	2	1.572	0.006819	ALCAM,KRT7
5	Mesenchymal cell	66	99	5	1.525104	1.90E-05	ALCAM,COL1A2,FN1,MDK,PDLIM7
5	Gonadal endothelial cell	455	99	15	1.478618	3.93E-09	ADM,ANXA2,CSRP1,CTGF,CYR61,DAB2,EDIL3,FN1 GNG11,LAMC1,LOXL2,MDK,MYO1B,PDLIM7,TMEM204
5	Unrestricted somatic stem cell	7	99	1	1.394689	0.03621	ALCAM
5	Mesangial cell	5	99	1	1.314808	0.027281	FN1
5	Myofibroblast	5	99	1	1.314808	0.027281	FN1

5	Mesenchymal progenitor cell	8	99	1	1.304612	0.040644	ALCAM
5	Leydig precursor cell	216	99	10	1.287343	9.19E-08	BGN,COL1A1,FBN1,FBN2,INHBA,LOXL2,MDK,RCN SERPINE2,SULF1
5	Epithelial cell	61	99	3	1.259883	0.003208	ALCAM,CTNNB1,KRT7
5	Limbal epithelial stem cell	4	99	1	1.23	0.022786	ALCAM
5	Mesenchymal stem cell	83	99	4	1.207407	0.000699	ALCAM,CD59,FN1,ITGB5
5	Intestinal stem cell	10	99	1	1.16688	0.049452	ALCAM
5	Cancer stem cell	149	99	4	1.155117	0.005477	ALCAM,ALDH1B1,CTNNB1,MYC
5	Primitive endoderm cell	87	99	4	1.127862	0.000828	FN1,FST,PGM1,SERPINE2
5	Cardiomyocyte	24	99	2	1.098188	0.006332	ACTN1,ALCAM
5	Astrocyte	425	99	11	1.08753	5.28E-06	ALCAM,ANXA5,DDAH1,GNG11,GRAMD2B,HTRA1 LGALS3BP,PHACTR2,PSAT1,SERPINE2,VEPH1
5	Granulosa cell	182	99	6	1.065917	0.000234	AEBP1,COL1A1,COL1A2,FN1,LDHA,MDK
5	Oligodendrocyte progenitor cell	99	99	5	0.999008	0.000119	ALCAM,ANXA5,PSAT1,SERPINE2,STMN2
5	Microglial cell	433	99	11	0.9621	6.27E-06	ANXA5,AXL,C12orf75,CTSB,DAB2,HERPUD1, HTRA1,MAGED1,MDK,MYL12A,RCN3
5	Endothelial cell	367	99	8	0.95734	0.000339	AEBP1,ALCAM,ANXA2,DAB2,FN1,GNG11,LDHA,M
5	Migration phase fetal germ cell	147	99	5	0.936957	0.000689	COL1A2,LDHA,MYL9,PDLIM7,TPM4
5	Cancer stem-like cell	7	99	1	0.929793	0.03621	ALCAM
5	PROM1Low progenitor cell	7	99	1	0.929793	0.03621	ALCAM
5	Naive CD4+ T cell	30	99	3	0.883659	0.000466	ACTN1,MYC,TMEM204
5	Bone marrow stem cell	8	99	1	0.869741	0.040644	ALCAM
5	Stem cell	70	99	2	0.853393	0.043462	ALCAM,CTNNB1
5	Mesenchymal stromal cell	58	99	2	0.83117	0.031178	ALCAM,CD59
5	MKI67+ progenitor cell	105	99	4	0.815852	0.001618	BGN,GNG11,HEG1,SERPINE2
5	Interneuron	28	99	2	0.812624	0.008375	LDHA,MDK
5	Idiopathic pulmonary fibrosis cell	13	99	1	0.798768	0.062513	COL1A1
5	Circulating fetal cell	39	99	2	0.797438	0.015279	COL1A2,CTNNB1

5	Exhausted CD8+ T cell	242	99	7	0.793245	0.000153	ANXA5,CSF1,CTNNB1,CTSB,GSTO1,LDHA,PDLIM7
5	Naive CD8+ T cell	88	99	4	0.780315	0.000863	ACTN1,LMO7,MYC,TMEM204
5	Adipose-derived stromal cell	21	99	2	0.772491	0.004974	ALCAM,MYC
5	Plasmacytoid dendritic cell	308	99	7	0.765815	0.000634	CRIM1,CTSB,DAB2,HERPUD1,MAGED1,SLC38A2,TGFBI
5	Liver bud hepatic cell	191	99	6	0.765542	0.000301	CSRP1,MYC,PDLIM7,SERPINE1,SLC38A2,TGFBI
5	Retinoid acid signaling-responsive fetal germ cell	205	99	5	0.69424	0.002869	FBN2,FST,MDK,NEXN,SERPINE2
5	Lake et al.Science.Ex1	14	99	1	0.684189	0.066827	SERPINE2
5	Lymphocyte	8	99	1	0.671751	0.040644	ANXA5
5	Stromal cell	42	99	2	0.663504	0.017471	ALCAM,GREM1
5	Regulatory T (Treg) cell	436	99	7	0.658984	0.004374	CD59,CSF1,CTNNB1,GSTO1,HERPUD1,PHACTR2,SERPINE2
5	CD141+CLEC9A+ dendritic cell	96	99	4	0.647074	0.001177	ACTN1,CD59,CSRP1,PDLIM7
5	Leydig cell	373	99	6	0.642048	0.008135	ALCAM,ALDH1B1,CAV2,MPC2,PSAT1,TGFBI
5	Cardiac progenitor cell	17	99	1	0.596638	0.079652	ALCAM
5	Trophectoderm cell	89	99	4	0.580879	0.000898	ACTN1,DAB2,MYC,PALLD
5	Oviduct-derived stem cell	18	99	1	0.579828	0.083888	ALCAM
5	Ionocyte cell	278	99	4	0.568573	0.041282	ADM,KRT7,LTBP2,THBS1
5	Pericyte	19	99	1	0.564363	0.088105	ALCAM
5	Dendritic cell progenitor	11	99	1	0.560811	0.053825	AXL
5	PROCR+ progenitor cell	21	99	1	0.528087	0.096481	RCN3
5	Lake et al.Science.In3	11	99	1	0.52463	0.053825	CRIM1
5	Osteoblast	22	99	1	0.524474	0.100641	ALCAM
5	Fibroblast	31	99	1	0.517263	0.137232	COL1A1
5	Endometrial stem cell	23	99	1	0.512945	0.104781	ALCAM
5	Adipose-derived stem cell	34	99	2	0.487056	0.011915	CD59,MYC
5	Macrophage	74	99	1	0.47429	0.292706	TGFBI

5	Parietal progenitor cell	6	99	1	0.465403	0.031756	ANXA1
5	Induced pluripotent stem cell	30	99	1	0.456435	0.133241	VSIR
5	Beta cell	97	99	2	0.436599	0.076231	ALCAM,GREM1
5	Neuron	75	99	1	0.426084	0.29597	ALCAM
5	Neural progenitor cell	172	99	3	0.411746	0.047009	LDHA,MDK,STMN2
5	Cancer cell	46	99	1	0.389247	0.194972	NDUFA4L2
5	Lake et al.Science.Ex6	28	99	1	0.377964	0.125202	SULF1
5	AXL+SIGLEC6+ dendritic cell	73	99	2	0.376872	0.046772	AXL,DAB2
5	Granulocyte-monocyte progenitor	104	99	2	0.366737	0.085693	ANXA1,LDHA
5	Lake et al.Science.Ex4	10	99	1	0.3605	0.049452	ANXA1
5	Ciliated cell	249	99	3	0.359955	0.110264	C12orf75,CD59,CTGF
5	Pit progenitor cell	52	99	1	0.357782	0.216981	ALDH1B1
5	Enteroendocrine cell	40	99	1	0.344688	0.17235	LOX
5	Embryonic stem cell	27	99	1	0.338712	0.121155	CD59
5	Alpha cell	92	99	2	0.335708	0.069694	ELL2,PALLD
5	Mucosal-associated invariant T cell	58	99	1	0.315135	0.238395	PHACTR2
5	Neural stem cell	60	99	1	0.286601	0.245403	CTNNB1
5	Morula cell (Blastomere)	96	99	1	0.265361	0.361173	LDHA
5	Brush cell (Tuft cell)	76	99	2	0.254651	0.050172	ANXA1,MYC
5	CD1C-CD141- dendritic cell	284	99	2	0.253971	0.37957	CDC42EP3,HEG1
5	Neuroendocrine cell	78	99	2	0.251366	0.052487	ANXA1,MYC
5	Neutrophil	90	99	1	0.246658	0.343179	ADM
5	Sertoli cell	436	99	3	0.227963	0.45793	AXL,ITGB5,MAP1B
5	Acinar cell	73	99	1	0.224719	0.289426	PSAT1
5	Secretory progenitor cell	115	99	1	0.218206	0.414996	ADM
5	Basal cell	86	99	1	0.1725	0.3309	IGFBP6

					32	06	
5	CD1C+_B dendritic cell	45	99	1	0.169941	0.191245	ANXA1
5	FOXN4+ cell	77	99	1	0.166382	0.302453	ACTN1
5	FGFR1HighNME5-epithelial cell	189	99	2	0.162936	0.219307	FBN1,THBS2
5	Paneth cell	271	99	2	0.159154	0.358113	DAB2,INHBA
5	CD4+ cytotoxic T cell	72	99	1	0.153206	0.286132	C12orf75
5	Effector CD8+ memory T (Tem) cell	73	99	1	0.152153	0.289426	C12orf75
5	Hematopoietic stem cell	51	99	1	0.15123	0.213355	MYC
5	Ciliated epithelial cell	374	99	1	0.135477	1	CTGF
5	Mast cell	101	99	1	0.123385	0.375795	CSF1
5	Gonadal mitotic phase fetal germ cell	275	99	1	0.101308	1	LOXL2
5	B cell	260	99	1	0.066979	1	MYC
6	Migration phase fetal germ cell	147	58	12	1.819478	1.18E-14	DBI,FABP5,GSTP1,LDHA,LGALS1,MGST1,MGST3,M MYL9,NQO1,TPM2,TXN
6	Basal cell	86	58	7	1.1991	5.68E-09	FABP5,HSPB1,LGALS3,S100A10,S100A6,SH3BGRL3 TIMP1
6	Granulocyte-monocyte progenitor	104	58	5	0.858989	1.13E-05	ANXA1,LDHA,LGALS1,MGST1,NQO1
6	Liver stem cell	7	58	1	0.771048	0.021396	GSTP1
6	Parietal progenitor cell	6	58	1	0.726682	0.018746	ANXA1
6	Microglial cell	433	58	9	0.708359	2.65E-06	ANXA5,CCND1,FABP5,FTL,MYL12A,PRDX2,S100A1 SRGN,UCP2
6	Lymphocyte	8	58	1	0.707107	0.024039	ANXA5
6	Astrocyte	425	58	9	0.685891	2.28E-06	ANXA5,CRYAB,DBI,FABP5,HSPB1,LGALS1,LGALS3, TIMP1,UCHL1
6	Morula cell (Blastomere)	96	58	3	0.630744	0.002437	LDHA,S100A11,TXN
6	Tuft progenitor cell	10	58	1	0.588184	0.029303	TPM2
6	Lake et al.Science.Ex4	10	58	1	0.562885	0.029303	ANXA1
6	Exhausted CD8+ T cell	242	58	5	0.547686	0.000538	ANXA5,GAPDH,LDHA,PKM,SRGN
6	Granulosa cell	182	58	4	0.544077	0.001602	FABP5,GAPDH,LDHA,SRGN

6	Endothelial cell	367	58	6	0.532436	0.000497	ANXA2,FABP5,HSPB1,LDHA,S100A4,SH3BGRL3
6	CD1C-CD141-dendritic cell	284	58	6	0.524557	0.000129	FTL,LGALS3,S100A11,S100A4,TAGLN,TIMP1
6	Neutrophil	90	58	2	0.508073	0.025668	FTL,S100A11
6	Regulatory T (Treg) cell	436	58	7	0.505732	0.000183	ENO1,GAPDH,HSPB1,LGALS3,PKM,PRDX1,UCP2
6	Smooth muscle cell	11	58	1	0.500509	0.031925	TAGLN
6	Macrophage	74	58	2	0.499865	0.017961	LGALS3,S100A11
6	Acinar cell	73	58	3	0.498595	0.001141	AKR1C2,AKR1C3,MGST1
6	CD1C+_B dendritic cell	45	58	2	0.488954	0.007161	ANXA1,MGST1
6	Exhausted CD4+ T cell	102	58	3	0.455468	0.002879	GAPDH,PKM,SRGN
6	AXL+SIGLEC6+ dendritic cell	73	58	2	0.451779	0.017519	S100A10,TXN
6	Sertoli cell	436	58	6	0.447305	0.001201	MAP1B,MGST3,PRDX1,PRDX6,S100A10,UCHL1
6	Liver bud hepatic cell	191	58	4	0.439933	0.001904	AKR1C2,HSPB1,S100A6,TIMP1
6	Fat cell (adipocyte)	22	58	1	0.430665	0.060308	FABP4
6	CD4+ cytotoxic T cell	72	58	2	0.407765	0.017081	AKR1C3,GSTP1
6	Gonadal endothelial cell	455	58	5	0.402237	0.00795	ACTB,ANXA2,CCND1,HSPB1,S100A11
6	Oligodendrocyte progenitor cell	99	58	2	0.389955	0.030494	ANXA5,DBI
6	Leydig cell	373	58	5	0.388335	0.003503	CYB5A,MPC2,S100A4,S100A6,TXN
6	Neural progenitor cell	172	58	3	0.379721	0.011808	HSPB1,LDHA,UCP2
6	Interneuron	28	58	1	0.362846	0.075443	LDHA
6	Lake et al.Science.In3	11	58	1	0.361814	0.031925	IGFBP5
6	Spermatogonial stem cell	9	58	1	0.36	0.026674	UCHL1
6	Leydig precursor cell	216	58	3	0.349733	0.021373	IGFBP5,NPM1,TPM2
6	Mast cell	101	58	2	0.316422	0.031611	ADIRF,PRDX6
6	Ciliated cell	249	58	3	0.30799	0.030663	NQO1,PRDX1,UCP2
6	Oligodendrocyte	137	58	2	0.285355	0.054289	FDPS,NPM1
6	Megakaryocyte	23	58	1	0.2835	0.0628	SRGN

					8	47	
6	Circulating fetal cell	39	58	1	0.269015	0.102571	ACTB
6	PROCR+ progenitor cell	21	58	1	0.261861	0.057762	IGFBP5
6	Cancer cell	46	58	1	0.256549	0.119426	NDUFA4L2
6	1-cell stage cell (Blastomere)	41	58	1	0.256125	0.107419	FTL
6	Stem cell	70	58	1	0.243827	0.174885	GSTP1
6	Plasmacytoid dendritic cell	308	58	2	0.227921	0.203974	TPM2,TXN
6	Effector CD8+ memory T (Tem) cell	73	58	1	0.22706	0.181571	LGALS1
6	Naive CD8+ T cell	88	58	1	0.221729	0.214213	NPM1
6	Trophectoderm cell	89	58	1	0.20988	0.216343	S100A6
6	LGR5+ stem cell	52	58	1	0.208013	0.133625	MGST1
6	Brush cell (Tuft cell)	76	58	1	0.20418	0.188205	ANXA1
6	Neuroendocrine cell	78	58	1	0.201545	0.192597	ANXA1
6	MKI67+ progenitor cell	105	58	1	0.197132	0.249661	FABP4
6	Mesenchymal cell	66	58	1	0.195715	0.165886	S100A4
6	Stromal cell	42	58	1	0.191336	0.109833	TIMP1
6	Paneth cell	271	58	2	0.176162	0.16801	SRGN,UCP2
6	FOXN4+ cell	77	58	1	0.164103	0.190404	ENO1
6	Enterocyte progenitor cell	110	58	1	0.143019	0.259784	MGST1
6	Oocyte	86	58	1	0.140183	0.209936	REXO2
6	T cell	69	58	1	0.130017	0.172644	UCHL1
6	B cell	260	58	1	0.128996	0.508264	NPM1
6	CD141+CLEC9A+ dendritic cell	96	58	1	0.112268	0.231095	CCND1
6	Gonadal mitotic phase fetal germ cell	275	58	1	0.10975	0.528046	MGST3
6	Retinoid acid signaling-responsiv	205	58	1	0.076827	0.428512	CCND1

	e fetal germ cell						
6	Ionocyte cell	278	58	1	0.071971	0.531907	IGFBP5
7	Astrocyte	425	182	24	3.471655	6.39E-13	ALDH1L1,APOE,CRYAB,DBI,ELOVL5,FABP5,FADS2,GLUL,HMGCS1,IDI1,ITGA7,LGALS1,LPL,MSMO1,PFKFB3,PNPLA3,PPP1R16A,RASSF4,RHOB,SCD,SELENBP1,SOX4,SPRY1,TNS1
7	Fat cell (adipocyte)	22	182	3	2.362264	0.001179	FABP4,LGALS12,PPARG
7	Microglial cell	433	182	16	2.125558	1.39E-06	APOC1,APOE,CD36,CEBPA,FABP5,FRMD4A,GLUL,GYPC,PDK4,RASSF4,RHOB,SAT1,SOX4,SPRY1,STOM,UCP2
7	Migration phase fetal germ cell	147	182	8	1.987734	5.21E-05	DBI,ETFB,FABP5,GLIPR2,HADH,LAMA4,LGALS1,SAT1
7	Liver bud hepatic cell	191	182	9	1.810384	5.18E-05	ACSL1,AKR1C2,ELOVL5,HP,IDI1,LBP,PALMD,PPIF,RHOB
7	Leydig cell	373	182	12	1.728351	0.000109	ACSF2,APOC1,APOE,CAV2,CYB5A,ETFB,GHR,HSPB6,LAMA4,MPC2,PNPLA2,PRKAR2B
7	Gonadal mitotic phase fetal germ cell	275	182	9	1.647458	0.000704	APOC1,APOE,COL4A1,COL4A2,GLIPR2,HILPDA,RASD1,SH3PXD2A,STOM
7	Primitive endoderm cell	87	182	5	1.485948	0.001091	COL4A1,COL4A2,DUSP4,ITIH5,PGM1
7	Neutrophil	90	182	3	1.441999	0.043955	DGAT2,G0S2,SAT1
7	Alpha cell	92	182	4	1.409557	0.008863	CD36,FADS2,PLA2G16,SORT1
7	CD1C-CD141-dendritic cell	284	182	11	1.402776	4.32E-05	CAMK1,CFD,CKB,DUSP6,GLUL,GNG2,GPBAR1,INSIG1,SAT1,TKT,TSPAN14
7	Sertoli cell	436	182	10	1.315095	0.004663	CKB,GNG2,GPC1,HK2,PLA2G16,PPIF,PRDX6,PTPRF,SAT1,TECR
7	Lake et al.Science.Ex2	7	182	1	1.300198	0.065434	CLMN
7	Gonadal endothelial cell	455	182	11	1.234838	0.002056	AKAP12,CKS1B,DUSP6,FRMD4A,FZD4,GYPC,LAMA4,PDK4,PIM3,SHC1,SPRY1
7	MKI67+ progenitor cell	105	182	4	1.208164	0.013651	FABP4,FZD4,NRN1,PALMD
7	Enterocyte progenitor cell	110	182	4	1.189921	0.015856	CHCHD10,FAH,G0S2,RBP4
7	Cytotoxic T cell	12	182	1	1.166248	0.104149	APOE
7	Endothelial cell	367	182	9	1.149435	0.004716	AKAP12,CD36,CKS1B,FABP5,GYPC,LAMA4,LMO4,SEMA3G,SOX4
7	Oligodendrocyte	137	182	5	1.141422	0.007108	ACAT2,DHCR24,EBP,FDPS,SCD

7	Granulosa cell	182	182	5	1.083707	0.021234	FABP5,GNG2,HILPDA,PRKAR2B,SPRY1
7	Mast cell	101	182	3	1.056729	0.05775	ADIRF,LGALS12,PRDX6
7	Ionocyte cell	278	182	5	1.036386	0.091626	ACSL1,AGPAT2,DUSP6,HILPDA,TSPAN14
7	Enterocyte	44	182	2	1.007048	0.05735	DGAT1,MLXIPL
7	Basal cell	86	182	3	1.000688	0.039403	CAV1,FABP5,GPC1
7	Epiblast cell	79	182	3	0.996828	0.032051	RASD1,SAT1,SH3PXD2A
7	Retinoid acid signaling-responsive fetal germ cell	205	182	4	0.965231	0.100629	FAM213A,PEMT,RBP4,SLC25A1
7	Multipotent progenitor cell	50	182	2	0.958837	0.071136	C2CD2,GPT2
7	Oligodendrocyte progenitor cell	99	182	5	0.950766	0.001882	DBI,DUSP6,KANK1,SOX4,SPRY1
7	CD141+CLEC9A+ dendritic cell	96	182	3	0.914476	0.051251	ACSS2,ELOVL5,GYPC
7	Acinar cell	73	182	3	0.898876	0.026381	AKR1C2,GPT2,PPIF
7	Cancer cell	46	182	2	0.83747	0.061832	FASN,GLUL
7	Trophectoderm cell	89	182	2	0.807718	0.178647	FASN,GLIPR2
7	Delta cell	15	182	1	0.805581	0.126608	RBP4
7	Granulocyte-monocyte progenitor	104	182	3	0.786426	0.061828	LGALS1,PDE3B,RETSAT
7	M2 macrophage	15	182	1	0.774597	0.126608	CD36
7	Platelet	38	182	1	0.729996	0.281178	CD36
7	Neural progenitor cell	172	182	4	0.710643	0.06188	CKS1B,GNG2,LMO4,UCP2
7	Secretory progenitor cell	115	182	2	0.684459	0.258813	APOE,GPT2
7	Ciliated cell	249	182	3	0.66541	0.473918	CKB,PLA2G16,UCP2
7	Regulatory T (Treg) cell	436	182	6	0.664731	0.281055	DUSP4,NDUFC2,PIM3,SAT1,SOX4,UCP2
7	AXL+SIGLEC6+ dendritic cell	73	182	2	0.65543	0.131682	PLA2G16,SOX4
7	Mesenchymal cell	66	182	2	0.642538	0.112185	AKAP12,HSPB6
7	T helper cell	42	182	1	0.623386	0.305134	APOE
7	Paneth cell	271	182	4	0.564935	0.298669	DUSP6,PFKFB3,PPIF,UCP2

7	Megakaryocyte erythroid cell	56	182	1	0.539868	0.382895	APOE
7	Fibroblast	31	182	1	0.538816	0.237262	CD36
7	Myeloid cell	57	182	1	0.535111	0.388106	APOE
7	Epithelial cell	61	182	1	0.504465	0.408517	FZD4
7	T cell	69	182	1	0.486359	0.447328	APOE
7	FOXN4+ cell	77	182	1	0.476355	0.483605	INSIG1
7	Plasmacytoid dendritic cell	308	182	3	0.46268	0.748311	KANK1,PLA2G16,RASD1
7	Monocyte	43	182	1	0.457496	0.310998	CD36
7	CD1C+_B dendritic cell	45	182	1	0.447214	0.322578	CD36
7	Mucosal-associated invariant T cell	58	182	1	0.44119	0.393274	ADAM12
7	Glutamatergic neuron	7	182	1	0.430879	0.065434	GLUL
7	Goblet cell	64	182	1	0.415	0.423378	ACSS2
7	Leydig precursor cell	216	182	2	0.39464	0.706803	HSPB6,INSIG1
7	Effector CD8+ memory T (Tem) cell	73	182	2	0.36985	0.131682	GLUL,LGALS1
7	Morula cell (Blastomere)	96	182	1	0.367423	0.56055	AKAP12
7	FGFR1HighNME5-epithelial cell	189	182	2	0.360787	0.676922	CSAD,GHR
7	FGFR1LowNME5-epithelial cell	49	182	1	0.345714	0.345162	GPC1
7	Exhausted CD4+ T cell	102	182	2	0.330709	0.218396	CAMK1,DUSP4
7	Naive CD8+ T cell	88	182	1	0.317669	0.52965	PDE3B
7	Lake et al.Science.Ex6	28	182	1	0.31749	0.217634	GHR
7	Beta cell	97	182	1	0.316788	0.564268	RBP4
7	Exhausted CD8+ T cell	242	182	2	0.214703	1	CAMK1,DUSP4
7	Ciliated epithelial cell	374	182	1	0.17581	0.383022	CEBPA
7	Neuron	75	182	1	0.157039	0.474764	CDO1
7	Brush cell (Tuft cell)	76	182	1	0.135355	0.479203	SOX4

7	CD4+ cytotoxic T cell	72	182	1	0.13435	0.461219	GLUL
7	Neuroendocrine cell	78	182	1	0.133609	0.48797	SOX4
7	Cancer stem cell	149	182	1	0.096669	1	SOX4
8	Neural progenitor cell	172	174	53	13.5617	8.41E-64	ASPM,AURKB,BIRC5,C19orf48,CCNA2,CCNB1,CDC20,CDCA3,CDCA5,CDCA7,CDKN3,CENPF,CENPM,CENPN,CENPU,CENPW,CKAP2,CKAP2L,CKS1B,CKS2,CLSPN,DHFR,GTSE1,H2AFZ,HELLS,HIST1H4C,HMGB2,HMGB3,KIF22,KIF23,KNSTRN,MCM4,MKI67,NUSAP1,ORC6,PCLAF,PCNA,PHGDH,PIMREG,PRC1,PRR11,PTTG1,RRM2,SMC4,TACC3,TOP2A,TPX2,TUBA1B,TUBB4B,UBE2C,UBE2T,UHRF1,ZWINT
8	MKI67+ progenitor cell	105	174	27	8.558644	1.67E-30	ANLN,ARHGAP29,ASPM,AURKB,BIRC5,CCNA2,CCNB1,CDC20,CDKN3,CENPF,CENPW,HIST1H4C,KIF20B,KNSTRN,MKI67,MXD3,NUSAP1,PRC1,PTTG1,RRM2,TACC3,TK1,TOP2A,TPX2,TROAP,UBE2C,UBE2T
8	Endothelial cell	367	174	29	4.674994	1.24E-19	ALCAM,ANPEP,BIRC5,C19orf48,CD44,CDCA5,CDH2,CENPN,CENPU,CKS1B,CLEC2B,COTL1,DCBLD2,H2AFZ,HELLS,HIST1H4C,HMGB3,ITGA5,KIF20B,KIF22,KIF23,PRR11,PTTG1,SMTN,STMN1,TACC3,TIMP3,TUBB,TUBB6
8	Interneuron	28	174	5	3.273172	6.33E-06	CKS2,HMGB2,PTTG1,UBE2C,UBE2T
8	FOXN4+ cell	77	174	9	3.220526	3.13E-08	CDT1,CENPM,DHFR,ENO1,HELLS,TCF19,TMEM106C,TYMS,UBE2T
8	Granulocyte-monocyte progenitor	104	174	10	2.95547	2.93E-08	ANXA1,ASPM,CDKN3,CLSPN,MCM4,MYBL2,RAD51AP1,SHCBP1,TCF19,UHRF1
8	Mesenchymal cell	66	174	8	2.808948	1.45E-07	ALCAM,CD44,CDH2,COL1A2,COTL1,FOSL1,ITGA5,TNC
8	Cancer stem-like cell	7	174	3	2.721344	5.91E-05	ALCAM,ANPEP,CD44
8	Myoblast	7	174	2	2.517243	0.002238	CDC20,PCNA
8	Mesenchymal stem cell	83	174	5	2.322612	0.000731	ALCAM,ANPEP,CD44,CDH2,ITGA5
8	Cancer stem cell	149	174	8	2.232407	4.16E-05	ALCAM,ALDH1A3,ANPEP,CD44,CDH2,FOXM1,ITGA5,MKI67
8	Immature neuron	4	174	1	2.18	0.0396	STMN1

						34	
8	Gonadal endothelial cell	455	174	18	2.119946	5.64E-08	ACTB,ARHGAP29,BIRC5,CALM1,CCND1,CDCA5,CDCA7,CKS1B,COTL1,CYR61,ITGA5,KLF6,LOXL2,SMTN,TIMP3,TUBA1C,TUBB6,UBE2C
8	Basal cell	86	174	6	1.951773	0.000101	CAV1,IGFBP6,PLP2,PTTG1,TIMP1,TUBB6
8	Unrestricted somatic stem cell	7	174	2	1.927619	0.002238	ALCAM,CD44
8	Astrocyte	425	174	13	1.867524	5.81E-05	ALCAM,ANLN,H2AFZ,HIF1A,MGLL,MT2A,PHGDH,STMN1,TIMP1,TIMP3,TMSB10,TNC,TUBB
8	Mesenchymal progenitor cell	8	174	2	1.803122	0.002782	ALCAM,CD44
8	Epithelial cell	61	174	5	1.687526	0.000192	ALCAM,ANPEP,BIRC5,CDH2,KLF6
8	Gonadal mitotic phase fetal germ cell	275	174	9	1.664343	0.000511	CDCA7,CDT1,DDIT4,LOXL2,MYBL2,RRM2,TUBA1C,TUBB,UPP1
8	Progenitor cell	25	174	3	1.66	0.001451	ALCAM,ASPM,CD44
8	Radial glial cell	10	174	2	1.650709	0.004038	CDH2,TNC
8	Intestinal stem cell	10	174	2	1.612762	0.004038	ALCAM,CD44
8	Liver bud hepatic cell	191	174	9	1.60923	3.66E-05	EMP2,MGLL,MT1M,MT2A,PLP2,PRG4,SERPINE1,TIMP1,TUBA1B
8	Mesenchymal stromal cell	58	174	4	1.578303	0.001577	ALCAM,ANPEP,CD44,ITGA5
8	Adipose-derived stromal cell	21	174	3	1.571169	0.000918	ALCAM,ANPEP,CD44
8	Stem cell	70	174	4	1.534674	0.003018	ALCAM,ANPEP,CD44,SMTN
8	Stromal cell	42	174	5	1.532232	3.73E-05	ALCAM,ANPEP,CD44,KLF6,TIMP1
8	Granulosa cell	182	174	8	1.509184	0.000158	ARHGAP29,COL1A2,HMGB3,MCM4,MT2A,PCNA,PGK1,SYNE2
8	Neural stem cell	60	174	3	1.404602	0.014326	CDH2,HMGB2,MKI67
8	Activated T cell	10	174	1	1.397727	0.085138	MKI67
8	Mesoderm cell	20	174	3	1.38189	0.000808	ANPEP,CD44,CDH2
8	Bone marrow stem cell	8	174	2	1.378858	0.002782	ALCAM,CD44
8	Pericyte	19	174	3	1.30767	0.000707	ALCAM,ANPEP,CD44
8	Migration phase fetal germ cell	147	174	6	1.304812	0.001523	ADAMTS5,COL1A2,H2AFZ,HMGB3,NUSAP1,TMSB10
8	Limbal epithelial stem cell	4	174	1	1.2	0.039634	ALCAM

8	Plasmacytoid dendritic cell	308	174	7	1.169236	0.01412	CRIM1,GAS6,IGFBP3,MYBL2,PLP2,STMN1,TUBB6
8	Dental pulp stem cell	12	174	2	1.169134	0.005508	ANPEP,CD44
8	Secretory cell	60	174	4	1.156731	0.001774	ALDH1A3,ANPEP,CREB3L1,CXCL6
8	Regulatory T (Treg) cell	436	174	9	1.152265	0.010216	CDKN1A,CKAP2,DDIT4,ENO1,GADD45A,KIF20B,PGK1,PTTG1,SMC4
8	Sertoli cell	436	174	8	1.102458	0.027774	ANKH,CDH2,GAS6,PHGDH,SYNE2,TMEM97,TUBA1A,TUBB4B
8	Primitive endoderm cell	87	174	3	1.102132	0.036251	ANKRD1,CDH2,CERS6
8	Retinoid acid signaling-responsive fetal germ cell	205	174	6	1.069995	0.007368	CCND1,CDKN3,COTL1,FBN2,TMEM97,UBE2T
8	FGFR1LowNME5- epithelial cell	49	174	2	1.065714	0.063625	C19orf48,DIAPH3
8	Oligodendrocyte	137	174	4	1.033773	0.027398	RGMB,TMEM97,TMSB10,TUBB
8	Granulocyte	12	174	2	0.952628	0.005508	ANPEP,CD44
8	Alpha cell	92	174	3	0.939357	0.0415	HMGB2,HMGB3,KCNMA1
8	Oviduct-derived stem cell	18	174	2	0.919239	0.011143	ALCAM,CD44
8	PROM1Low progenitor cell	7	174	1	0.907115	0.062661	ALCAM
8	Leydig precursor cell	216	174	8	0.900868	0.000479	ADAMTS1,ALDH1A3,CRISPLD2,EMP2,FBN2,LOXL2,POSTN,TIMP3
8	Acinar cell	73	174	3	0.889513	0.023518	ANPEP,PTGR1,TMEM97
8	Microglial cell	433	174	7	0.871753	0.093402	C12orf75,CCND1,GAS6,KLF6,STMN1,SYNE2,TUBA1A
8	Type II pneumocyte	3	174	1	0.866025	0.031834	CD44
8	Delta cell	15	174	1	0.862384	0.121416	PRG4
8	Osteoblast	22	174	2	0.831483	0.01585	ALCAM,CD44
8	Adipose-derived stem cell	34	174	2	0.823193	0.033979	ANPEP,CD44
8	Endometrial stem cell	23	174	2	0.813206	0.017138	ALCAM,CD44
8	Basophil	27	174	2	0.80829	0.022712	ANPEP,CD44
8	CD4+ cytotoxic T cell	72	174	2	0.801388	0.119831	C12orf75,SYNE2
8	Hematopoietic stem cell	51	174	2	0.80096	0.06806	CD44,ITGA5
8	Circulating fetal cell	39	174	3	0.781425	0.004683	ACTB,COL1A2,TNC

8	Goblet cell	64	174	2	0.7575	0.0991 15	CENPM,MT1M
8	Fibroblast	31	174	2	0.7525 46	0.0289 26	CD44,CKAP4
8	Dedifferentiation adipocyte	4	174	1	0.75	0.0396 34	CD44
8	Luminal progenitor	4	174	1	0.75	0.0396 34	CD44
8	Multipotent stem cell	4	174	1	0.75	0.0396 34	CD44
8	Neuron	75	174	2	0.7482 46	0.1278 55	ALCAM,CDH2
8	Myeloblast	6	174	1	0.7348 47	0.0550 47	ANPEP
8	Parietal progenitor cell	6	174	1	0.6940 22	0.0550 47	ANXA1
8	Exhausted CD4+ T cell	102	174	2	0.6911 23	0.2044 28	CKAP2,PTTG1
8	1-cell stage cell (Blastomere)	41	174	1	0.6871 65	0.2882 27	TROAP
8	CD1C-CD141-dendritic cell	284	174	5	0.6776 52	0.0848 83	MT2A,MXD3,TIMP1,TUBA1A,UPP1
8	Leydig cell	373	174	6	0.6669 01	0.1321	ALCAM,ANPEP,C19orf48,GADD45A,PGK1,RPL22L1
8	Ciliated cell	249	174	4	0.6654 1	0.148	C12orf75,CALM1,TUBA1A,TUBB4B
8	Endothelial stem cell	17	174	1	0.6596 97	0.1355 24	ITGA5
8	CD1C+_B dendritic cell	45	174	2	0.6588 95	0.0550 69	ANXA1,ITGA5
8	FGFR1HighNME5-epithelial cell	189	174	4	0.6575 63	0.0710 44	ABI3BP,ADAMTS1,POSTN,TROAP
8	AXL+SIGLEC6+ dendritic cell	73	174	3	0.6554 3	0.0235 18	ATF5,CDKN1A,MGLL
8	Epidermal stem cell	13	174	1	0.6489 99	0.1070 8	TNC
8	Effector memory T cell	6	174	1	0.6123 72	0.0550 47	CD44
8	LGR5+ stem cell	52	174	2	0.5824 35	0.0703 15	CDCA7,RGMB
8	Cardiac progenitor cell	17	174	1	0.5820 86	0.1355 24	ALCAM
8	Trophectoderm cell	89	174	2	0.5787 59	0.1667 6	EMP2,TMEM106C
8	CD1C+_A dendritic cell	25	174	1	0.544	0.1897 36	ITGA5
8	Oligodendrocyte progenitor cell	99	174	2	0.5387	0.1956 41	ALCAM,TMSB10
8	Lake et al.Science.Ex4	10	174	1	0.5375 87	0.0851 38	ANXA1
8	Myeloid-derived	26	174	1	0.5334	0.1962	ITGA5

	suppressor cell				36	71	
8	Adventitial cell	8	174	1	0.53033	0.070214	CD44
8	Osteocyte	8	174	1	0.53033	0.070214	CD44
8	Multipotent progenitor cell	50	174	1	0.528916	0.338297	UBE2S
8	Paneth cell	271	174	4	0.507834	0.288614	ATF5,CLEC2B,COTL1,METRNL
8	Keratinocyte	9	174	1	0.5	0.077706	CD44
8	Myoepithelial cell	9	174	1	0.5	0.077706	CD44
8	Cardiomyocyte	24	174	1	0.489898	0.183149	ALCAM
8	Oocyte	86	174	2	0.485247	0.158249	ABI3BP,CENPE
8	Exhausted CD8+ T cell	242	174	3	0.482118	0.452366	COTL1,KIF20B,PTGIS
8	Primordial germ cell	18	174	1	0.471405	0.142493	CCNA2
8	Pluripotent stem cell	15	174	1	0.464758	0.121416	ANPEP
8	Lake et al.Science.In3	11	174	1	0.446237	0.092511	CRIM1
8	Neutrophil	90	174	2	0.442719	0.169614	ANPEP,CD44
8	Urine-derived stem cell	12	174	1	0.433013	0.099825	CD44
8	Eosinophil	29	174	1	0.417815	0.215561	CD44
8	T cell	69	174	2	0.397273	0.11194	ANPEP,CD44
8	DCLK1+ progenitor cell	98	174	3	0.393959	0.048276	ADAMTS1,COL1A2,LOX
8	CD141+CLEC9A+ dendritic cell	96	174	2	0.391918	0.186904	ANPEP,CCND1
8	Macrophage	74	174	1	0.373155	0.455345	METRNL
8	Platelet	38	174	1	0.373109	0.270713	SLC44A1
8	Effector CD8+ memory T (Tem) cell	73	174	1	0.367509	0.450907	C12orf75
8	Immune cell	25	174	1	0.36	0.189736	ANPEP
8	Myeloid cell	57	174	1	0.357624	0.374806	ANPEP
8	Cancer cell	46	174	1	0.331744	0.316491	CD44
8	Dendritic cell	31	174	1	0.3232	0.2281	ANPEP

					9	65	
8	Enterocyte	44	174	1	0.27136	0.305322	ANPEP
8	Enteroendocrine cell	40	174	1	0.252982	0.282436	LOX
8	Epiblast cell	79	174	1	0.247519	0.477006	UPP1
8	Beta cell	97	174	1	0.243683	0.54813	ALCAM
8	Ionocyte cell	278	174	2	0.242303	1	KCNMA1,POSTN
8	Megakaryocyte erythroid cell	56	174	1	0.240535	0.369716	ANPEP
8	Morula cell (Blastomere)	96	174	1	0.212289	0.544444	WEE1
8	Brush cell (Tuft cell)	76	174	1	0.195003	0.464114	ANXA1
8	Lake et al.Science.Ex8	48	174	1	0.193412	0.327482	POSTN
8	Neuroendocrine cell	78	174	1	0.192487	0.472743	ANXA1
8	Mast cell	101	174	1	0.131345	0.562579	TIMP3
8	B cell	260	174	1	0.064498	0.727417	RPL22L1
9	Fibroblast	31	125	4	3.656764	4.91E-05	CD81,CKAP4,ITGB1,THY1
9	Mesenchymal stromal cell	58	125	7	3.421846	9.81E-08	ALCAM,CD59,CD63,CD81,ITGA3, ITGB1,THY1
9	Mesenchymal stem cell	83	125	9	3.289635	3.22E-09	ALCAM,CD59,CD63,CD81,ITGA3, ITGB1,ITGB5,LEP,THY1
9	Adipose-derived stem cell	34	125	3	2.641078	0.001281	CD59,ITGB1,THY1
9	Bone marrow stem cell	8	125	3	2.630437	3.04E-05	ALCAM,ITGB1,THY1
9	Unrestricted somatic stem cell	7	125	2	2.528582	0.00117	ALCAM,THY1
9	Embryonic stem cell	27	125	3	2.374834	0.00069	CD59,ITGB1,THY1
9	Mesenchymal progenitor cell	8	125	2	2.365272	0.001457	ALCAM,THY1
9	Liver stem cell	7	125	2	2.282905	0.00117	ITGB1,THY1
9	Adipose-derived stromal cell	21	125	3	2.282559	0.000353	ALCAM,ITGB1,THY1
9	Microglial cell	433	125	19	2.225036	1.55E-11	APLP2,APOC1,BRI3,BST2,C3, CD63,CD81,CTSB,FTL,GAS6, HIST1H1C,HLA-B, HLA-C, KCTD12, LGMN,METTL7A,SAT1, SRGN,STOM
9	Urine-derived stem	12	125	2	2.1852	0.0029	ITGB1,THY1

	cell				71	01	
9	Astrocyte	425	125	18	2.0591 27	9.69E-11	ALCAM,CD63,CD82,CLU,CRYAB, DKK3,FTH1,GPX3,HLA-A,HLA-B, HLA-C,IGFBP7,METTL7A,MT1X, MT2A,SELENBP1,TFPI,TMSB10
9	Pericyte	19	125	3	2.0578 59	0.0002 71	ALCAM,ITGB1,THY1
9	Cancer stem cell	149	125	7	1.9792 64	3.55E-05	ALCAM,DPP4,IFI27,ITGA3,ITGB1, KRT8,THY1
9	Stem cell	70	125	4	1.9267 09	0.0009 07	ALCAM,DPP4,ITGB1,THY1
9	Stromal cell	42	125	3	1.8500 97	0.0022 62	ALCAM,ITGB1,THY1
9	Progenitor cell	25	125	3	1.786	0.0005 61	ALCAM,ITGB1,THY1
9	Oviduct-derived stem cell	18	125	3	1.7536 25	0.0002 35	ALCAM,ITGB1,THY1
9	Dental pulp stem cell	12	125	2	1.7435 98	0.0029 01	ITGB1,THY1
9	Keratinocyte	9	125	2	1.72	0.0017 73	ITGB1,KRT8
9	Regulatory T (Treg) cell	436	125	13	1.6728 44	2.16E-06	APLP2,BST2,CD59,CD82,DPP4, GLRX,IL32,LAPTM4A,METTL7A, NAMPT,PKM,SAT1,SQSTM1
9	Lake et al.Science.Ex4	10	125	2	1.6696 83	0.0021 2	ANXA1,PAPSS2
9	Multipotent mesenchymal stroma cell	8	125	1	1.6228 1	0.0510 12	THY1
9	Mesenchymal cell	66	125	5	1.6161 91	5.80E-05	ALCAM,DKK3,IGFBP7,SVIL,THY1
9	Liver bud hepatic cell	191	125	11	1.5788 4	2.73E-08	CARHSP1,FTH1,HP,LBP,MT1X,MT2A, PCOLCE2,SAA1,SAA2,SOD2,SVIP
9	Endometrial stem cell	23	125	3	1.5513 47	0.0004 49	ALCAM,ITGB1,THY1
9	T helper1 (Th1) cell	18	125	2	1.5414 93	0.0059 21	DPP4,STAT1
9	Multipotent stem cell	4	125	1	1.53	0.0286 66	THY1
9	Neutrophil	90	125	4	1.5020 82	0.0021 99	FTL,NAMPT,SAT1,SOD2
9	Limbal stem cell	5	125	1	1.4936 93	0.0343 01	SOD2
9	Dedifferentiation adipocyte	4	125	1	1.49	0.0286 66	ITGB1
9	Myoepithelial cell	9	125	1	1.49	0.0565 18	ITGB1
9	Hematopoietic precursor cell	5	125	1	1.3684 74	0.0343 01	THY1
9	Retinal ganglion cell	5	125	1	1.3684 74	0.0343 01	THY1

9	Osteoclast	6	125	1	1.355384	0.039903	CTSK
9	Mesoderm cell	20	125	2	1.350585	0.007145	ITGB1,THY1
9	Colorectal stem cell	5	125	1	1.332697	0.034301	ITGB1
9	CD1C-CD141-dendritic cell	284	125	9	1.326822	5.41E-05	FTH1,FTL,ITGB1,MT2A,NAMPT,SAT1,SOD2,SVIL,TMTC1
9	Hematopoietic cell	12	125	1	1.325019	0.072847	THY1
9	Sertoli cell	436	125	10	1.289234	0.000278	CLU,GAS6,IL32,ITGB5,KRT8,PPM1K,PTGDS,SAT1,SLC39A14,SVIP
9	Osteoblast	22	125	2	1.277072	0.008472	ALCAM,THY1
9	Platelet	38	125	3	1.268572	0.001728	CCL5,CD63,ITGB1
9	Gonadal mitotic phase fetal germ cell	275	125	9	1.266348	4.25E-05	APOC1,CD63,CD81,COL4A1,COL4A2,MT1X,PCYOX1,STOM,THY1
9	Ionocyte cell	278	125	9	1.259496	4.61E-05	AKR1B1,APLP2,CELF2,KRT8,QSOX1,SEMA3C,SVIL,TFPI,USP53
9	Hepatocyte	12	125	1	1.258624	0.072847	KRT8
9	Common myeloid progenitor	6	125	1	1.24924	0.039903	THY1
9	Spermatogonium	6	125	1	1.21658	0.039903	ITGB1
9	Endocrine cell	6	125	1	1.20025	0.039903	SEZ6L2
9	Germinal center B cell	8	125	1	1.187939	0.051012	CD81
9	Hematopoietic stem cell	51	125	2	1.165033	0.03794	ITGA3,THY1
9	Oligodendrocyte	137	125	5	1.161926	0.001441	DHCR24,MAP1A,PPP1R14A,THY1,TMSB10
9	Beta cell	97	125	5	1.161556	0.000322	ALCAM,APLP2,LGMN,MXRA7,SLC43A2
9	Activated CD4+ T cell	7	125	1	1.126334	0.045474	ITGB1
9	T cell	69	125	3	1.106346	0.00849	CD81,DPP4,THY1
9	Adventitial cell	8	125	1	1.081873	0.051012	THY1
9	Alpha cell	92	125	5	1.067594	0.000255	CLU,DPP4,GLS,PLOD2,SORT1
9	Marginal zone B cell	10	125	1	1.062525	0.061992	CD81
9	Cardiac progenitor cell	17	125	2	1.062306	0.005349	ALCAM,ITGB1
9	CD8+ T cell	11	125	1	1.05529	0.067435	CCL5

9	Exhausted CD4+ T cell	102	125	4	1.031734	0.003396	BST2,CD82,PKM,SRGN
9	Endothelial progenitor cell	9	125	1	1.02	0.056518	THY1
9	Spermatogonial stem cell	9	125	1	1.02	0.056518	THY1
9	Migration phase fetal germ cell	147	125	5	1.016136	0.001943	ADAMTS5,CARHSP1,MYL9,SAT1,TMSB10
9	Parietal progenitor cell	6	125	1	1.012456	0.039903	ANXA1
9	Neural tube cell	12	125	1	0.969948	0.072847	CD81
9	Plasmacytoid dendritic cell	308	125	7	0.95499	0.002445	CRIM1,CTSB,GAS6,LGMN,MAP1A,PCYOX1,PPM1K
9	Lake et al.Science.In3	11	125	1	0.934685	0.067435	CRIM1
9	Endothelial cell	367	125	7	0.912971	0.006221	ALCAM,BST2,CD81,DCBLD2,ITGB1,KIF22,THY1
9	Oligodendrocyte progenitor cell	99	125	4	0.900514	0.003063	ALCAM,CD63,CD82,TMSB10
9	Exhausted CD8+ T cell	242	125	6	0.883241	0.003282	BST2,CD63,CD82,CTSB,PKM,SRGN
9	Follicular B cell	15	125	1	0.867548	0.088895	CD81
9	DCLK1+ progenitor cell	98	125	4	0.840447	0.002957	C3,LOX,PAMR1,PDGFRL
9	AXL+SIGLEC6+ dendritic cell	73	125	3	0.838015	0.009842	LGMN,PPP1R14A,PTGDS
9	Epidermal stem cell	13	125	1	0.826503	0.078227	ITGB1
9	Neural stem cell	60	125	2	0.81849	0.050352	CD81,ITGB1
9	Ciliated cell	249	125	5	0.811167	0.016348	CD59,HIST1H1C,IFI27,IGFBP7,SAA2
9	M1 macrophage	16	125	1	0.78	0.094183	STAT1
9	Neuron	75	125	2	0.772495	0.07371	ALCAM,THY1
9	Cancer cell	46	125	2	0.7608	0.031642	ITGB1,KRT8
9	Granulocyte-monocyte progenitor	104	125	3	0.751125	0.024427	ANXA1,APLP2,IGFBP7
9	Acinar cell	73	125	2	0.742041	0.070423	IL32,SOD2
9	Leydig cell	373	125	6	0.738355	0.023272	ALCAM,APOC1,GLRX,IFI27,PAPSS2,STAT1
9	Epithelial cell	61	125	2	0.737492	0.05181	ALCAM,KRT8
9	CD4+ cytotoxic T cell	72	125	2	0.730677	0.068798	CCL5,PTGDS
9	Endothelial stem	17	125	1	0.7227	0.0994	ITGB1

	cell				56	41	
9	Megakaryocyte	23	125	1	0.713119	0.130356	SRGN
9	Limbal epithelial stem cell	4	125	1	0.7	0.028666	ALCAM
9	Mast cell	101	125	3	0.694536	0.022692	CD82,CLU,LAPTM4A
9	Neural crest cell	19	125	1	0.683659	0.109865	ITGB1
9	Primitive endoderm cell	87	125	4	0.681864	0.001953	COL4A1,COL4A2,DPP4,ITIH5
9	FGFR1HighNME5- epithelial cell	189	125	4	0.676475	0.026137	CTSK,CYP1B1,PAMR1,PDGFRL
9	Natural killer cell	60	125	2	0.673899	0.050352	CD81,DPP4
9	Intestinal stem cell	10	125	1	0.664078	0.061992	ALCAM
9	Myeloid-derived suppressor cell	26	125	1	0.65895	0.145417	CD81
9	Multipotent progenitor cell	50	125	2	0.656195	0.036645	C2CD2,THY1
9	T helper cell	42	125	2	0.654246	0.026936	DPP4,IL32
9	Paneth cell	271	125	4	0.651194	0.076309	LGMN,NAMPT,SOD2,SRGN
9	Eosinophil	29	125	1	0.623936	0.160219	CD81
9	Gonadal endothelial cell	455	125	5	0.578508	0.196546	ANGPTL4,NRP2,SNCG,TFPI,THY1
9	Specialist antigen presenting cell	34	125	1	0.576235	0.184326	CD81
9	Enterocyte progenitor cell	110	125	3	0.572078	0.028109	DPP4,GLRX,IL32
9	Purkinje cell	23	125	1	0.554648	0.130356	CLU
9	Lymphoid cell	37	125	1	0.552381	0.198459	CD81
9	Plasma cell	37	125	1	0.552381	0.198459	CD81
9	Induced pluripotent stem cell	30	125	1	0.544071	0.165096	ITGB1
9	Cancer stem-like cell	7	125	1	0.52915	0.045474	ALCAM
9	PROM1Low progenitor cell	7	125	1	0.52915	0.045474	ALCAM
9	Leydig precursor cell	216	125	3	0.528001	0.134726	CYP1B1,TFPI,THY1
9	Trophectoderm cell	89	125	2	0.519399	0.097994	KRT8,TMEM106C
9	Basophil	27	125	1	0.515766	0.15038	CD63

9	Mucosal-associated invariant T cell	58	125	2	0.506843	0.047481	DKK3,DPP4
9	Epiblast cell	79	125	2	0.504039	0.080428	MT1X,SAT1
9	1-cell stage cell (Blastomere)	41	125	1	0.490386	0.216926	FTL
9	B cell	260	125	4	0.480014	0.06788	BST2,CD63,CD81,PPM1K
9	Dendritic cell	31	125	1	0.47775	0.169945	CLU
9	CD141+CLEC9A+ dendritic cell	96	125	2	0.465403	0.11087	CD59,DPP4
9	Naive CD4+ T cell	30	125	1	0.456435	0.165096	SVIL
9	Megakaryocyte erythroid cell	56	125	1	0.448999	0.282495	CD81
9	Nephron epithelial cell	17	125	1	0.446266	0.099441	SAA2
9	Myeloid cell	57	125	1	0.445043	0.286667	CD81
9	FOXN4+ cell	77	125	2	0.442167	0.077045	CARHSP1,TMEM106C
9	Granulosa cell	182	125	3	0.441785	0.092869	GPX3,MT2A,SRGN
9	Granulocyte	12	125	1	0.386825	0.072847	CD63
9	Myeloid conventional dendritic cell	11	125	1	0.373874	0.067435	DPP4
9	CD1C+_B dendritic cell	45	125	1	0.369697	0.234971	ANXA1
9	Pit progenitor cell	52	125	1	0.318953	0.265562	PPP1R14A
9	Goblet cell	64	125	1	0.3025	0.315207	IFI27
9	Secretory cell	60	125	2	0.294347	0.050352	C3,HLA-A
9	Cardiomyocyte	24	125	1	0.285774	0.135406	ALCAM
9	Brush cell (Tuft cell)	76	125	1	0.284476	0.361522	ANXA1
9	Fat cell (adipocyte)	22	125	1	0.281425	0.125278	LEP
9	Neuroendocrine cell	78	125	1	0.280805	0.368933	ANXA1
9	Retinoid acid signaling-responsive fetal germ cell	205	125	2	0.279372	0.338113	COX7A1,FAM213A
9	Lake et al.Science.Ex8	48	125	1	0.274241	0.248233	LTBP1
9	MKI67+ progenitor	105	125	1	0.2713	0.4610	TFPI

	cell					13	
9	Secretory progenitor cell	115	125	1	0.242451	0.491622	SRPX2
9	Enteroendocrine cell	40	125	1	0.218197	0.212349	LOX
9	Ciliated epithelial cell	374	125	2	0.18822	1	LTBP1,NRP2
9	Macrophage	74	125	1	0.155772	0.354024	CD63
9	Basal cell	86	125	1	0.116459	0.397733	MT1X
9	Neural progenitor cell	172	125	1	0.096074	1	KIF22
10	Microglial cell	433	154	17	2.371129	2.49E-08	BTG2,CD36,DUSP1,FABP5,IGF1,KCTD12,METTL7A,NABP1,NAV3,PDK4,PLTP,RHOB,RUNX1,SAT1,SOX4,STOM,UCP2
10	Astrocyte	425	154	15	1.771965	6.39E-07	ABCA1,BDH2,BTG2,FABP5,ITGA7,LPL,MAFB,METTL7A,MSMO1,PLTP,RHOB,SCD,SOX4,SRPX,TP53I3
10	Leydig precursor cell	216	154	10	1.403013	5.36E-06	ADAMTS1,ALDH1A3,CALD1,IGF1,IGFBP5,NRK,PCDH9,PHLDA1,SRPX,TSHZ2
10	Endothelial cell	367	154	10	1.367107	0.000389	AKAP12,ARL4A,CD36,FABP5,LAMA4,S1PR3,SEMA3G,SMTN,SOX4,TUBB6
10	Regulatory T (Treg) cell	436	154	11	1.26912	0.000377	CCND2,CDKN1A,CTNNB1,DDIT4,METTL7A,PHLDA1,PLTP,SAT1,SMC4,SOX4,UCP2
10	Cancer stem cell	149	154	6	1.268171	0.000873	ALDH1A3,CCND2,CTNNB1,PLAUR,SLC2A3,SOX4
10	Gonadal mitotic phase fetal germ cell	275	154	8	1.200015	0.000993	CCND2,DDIT4,ELOVL6,MDM2,NRK,PCYOX1,RASD1,STOM
10	Migration phase fetal germ cell	147	154	6	1.190991	0.000816	FABP5,HADH,LAMA4,PLTP,SAT1,TP53I3
10	Alpha cell	92	154	4	1.096786	0.004982	ARRDC4,CD36,MAFB,PTGER3
10	Gonadal endothelial cell	455	154	11	1.073569	0.000533	AKAP12,ANGPT1,DUSP6,LAMA4,MYO1B,NAV3,PDK4,SHC1,SMTN,TP53I3,TUBB6
10	Ionocyte cell	278	154	7	1.054378	0.004449	ACSL1,ARL4A,DUSP6,GPD1L,IGF1,IGFBP5,SEMA3C
10	Acinar cell	73	154	3	1.048689	0.017137	AKR1C2,PLTP,PPIF
10	Corneal endothelial cell	5	154	1	1.010703	0.042062	GPC4
10	Liver bud hepatic cell	191	154	5	0.994192	0.013376	ACSL1,AKR1C2,HP,PPIF,RHOB
10	Primitive endoderm cell	87	154	3	0.990632	0.026656	HMGCR,IGF1,PIK3R1
10	Pancreatic	15	154	1	0.9708	0.1082	LMO3

	polypeptide cell				28	94	
10	Basal cell	86	154	4	0.957555	0.00396	CCND2,FABP5,GPC1,TUBB6
10	Oligodendrocyte	137	154	5	0.953463	0.003548	ARL4A,LIMS2,PHLDA1,RGMB,SCD
10	Paneth cell	271	154	6	0.91483	0.014693	ABCA1,DUSP6,MAFB,PLAUR,PPIF,UCP2
10	Cancer cell	46	154	2	0.893498	0.046081	FASN,PLAUR
10	Plasmacytoid dendritic cell	308	154	7	0.888323	0.007574	IFI44L,NOP56,PCYOX1,RASD1,SLC20A1,TNFRSF21,TUBB6
10	Trophectoderm cell	89	154	3	0.860718	0.028205	ELOVL6,FASN,PPME1
10	Gastric stem cell	4	154	1	0.85	0.035176	RUNX1
10	Sertoli cell	436	154	8	0.841929	0.014565	BTG2,CUL4B,CXXC5,GPC1,LIMS2,NABP1,PPIF,SAT1
10	Stem cell	70	154	3	0.831879	0.015397	CTNNB1,RUNX1,SMTN
10	M2 macrophage	15	154	1	0.826236	0.108294	CD36
10	CD1C-CD141-dendritic cell	284	154	6	0.802264	0.018005	ARL4A,CFD,DUSP1,DUSP6,MAFB,SAT1
10	Circulating fetal cell	39	154	2	0.794236	0.034594	CTNNB1,MMP14
10	Neural progenitor cell	172	154	4	0.785368	0.037564	EMX2,LMO3,SMC4,UCP2
10	Platelet	38	154	1	0.778663	0.243863	CD36
10	Granulosa cell	182	154	3	0.733837	0.146276	FABP5,HIST1H1D,NRK
10	FGFR1HighNME5-epithelial cell	189	154	4	0.731757	0.049747	ADAMTS1,CSAD,ELOVL6,PCDH9
10	Mucosal-associated invariant T cell	58	154	2	0.714307	0.068452	ADAM12,DUSP1
10	Mesenchymal cell	66	154	2	0.713931	0.084955	AKAP12,PLTP
10	Oligodendrocyte progenitor cell	99	154	3	0.697496	0.036647	ARL4A,DUSP6,SOX4
10	AXL+SIGLEC6+ dendritic cell	73	154	3	0.671816	0.017137	CDKN1A,SLC20A1,SOX4
10	CD1C+_B dendritic cell	45	154	2	0.67082	0.044364	CD36,SLC2A3
10	Leydig cell	373	154	5	0.668972	0.199418	LAMA4,MAFB,PLTP,PNPLA2,RRP12
10	Lake et al.Science.In3	11	154	1	0.639204	0.082365	IGFBP5
10	Interneuron	28	154	1	0.63876	0.187638	PHLDA1
10	Neural stem cell	60	154	2	0.627423	0.072469	CTNNB1,RUNX1

10	Lake et al.Science.Ex6	28	154	1	0.616082	0.187638	PCSK5
10	Macrophage	74	154	1	0.6103	0.416093	PLTP
10	Epithelial cell	61	154	1	0.606895	0.358934	CTNNB1
10	FGFR1LowNME5- epithelial cell	49	154	2	0.588571	0.051376	GPC1,SLC2A3
10	Lake et al.Science.Ex4	10	154	1	0.575535	0.075767	TSHZ2
10	Fibroblast	31	154	1	0.574737	0.204928	CD36
10	Granulocyte-monocyte progenitor	104	154	2	0.562853	0.175085	PDE3B,RETSAT
10	Exhausted CD8+ T cell	242	154	3	0.560543	0.254915	CCND2,CTNNB1,PHLDA1
10	Epiblast cell	79	154	2	0.522041	0.11395	RASD1,SAT1
10	Monocyte	43	154	1	0.487995	0.270508	CD36
10	T helper cell	42	154	1	0.481426	0.265254	DUSP1
10	PROCR+ progenitor cell	21	154	1	0.462622	0.145828	IGFBP5
10	Naive CD8+ T cell	88	154	1	0.409345	0.471996	PDE3B
10	Oocyte	86	154	1	0.405451	0.464348	LMO3
10	Retinoid acid signaling-responsive fetal germ cell	205	154	2	0.403693	0.661296	FAM213A,SLC20A1
10	B cell	260	154	3	0.39443	0.439966	CXXC5,PAWR,PCDH9
10	Enteroendocrine cell	40	154	1	0.392122	0.254635	APCDD1
10	Smooth muscle cell	11	154	1	0.379904	0.082365	CALD1
10	Naive CD4+ T cell	30	154	1	0.332285	0.199205	TSHZ2
10	Goblet cell	64	154	1	0.3225	0.372599	ACSS2
10	Primordial germ cell	18	154	1	0.306413	0.127261	SLC2A3
10	LGR5+ stem cell	52	154	1	0.26903	0.316139	RGMB
10	DCLK1+ progenitor cell	98	154	2	0.26668	0.15993	ADAMTS1,SRPX
10	CD141+CLEC9A+ dendritic cell	96	154	1	0.26332	0.501518	ACSS2
10	CD1C+_A dendritic cell	25	154	1	0.26	0.169974	SLC2A3

10	Morula cell (Blastomere)	96	154	1	0.234743	0.501518	AKAP12
10	Ciliated cell	249	154	1	0.230676	1	UCP2
10	Natural killer cell	60	154	1	0.219469	0.354313	RUNX1
10	Neutrophil	90	154	1	0.172871	0.479535	SAT1
10	Pit progenitor cell	52	154	1	0.16641	0.316139	LIMS2
10	Brush cell (Tuft cell)	76	154	1	0.158297	0.424425	SOX4
10	Neuroendocrine cell	78	154	1	0.156254	0.432639	SOX4
10	Mast cell	101	154	1	0.151246	0.51913	ABCA1
10	Secretory cell	60	154	1	0.144591	0.354313	ALDH1A3
10	Ciliated epithelial cell	374	154	1	0.128238	0.528961	APCDD1

Table S5 Statistics of input gene lists

Name	Total	Unique
Input ID	46	44
