# Supplementary figures and images for: The role of serum amyloid A1 in the adipogenic differentiation of human adipose-derived stem cells basing on single-cell RNA sequencing analysis
Source: Stem Cell Res Ther. 2022 May 7;13:187. doi: 10.1186/s13287-022-02873-5 (PMC9080218; doi:10.1186/s13287-022-02873-5)

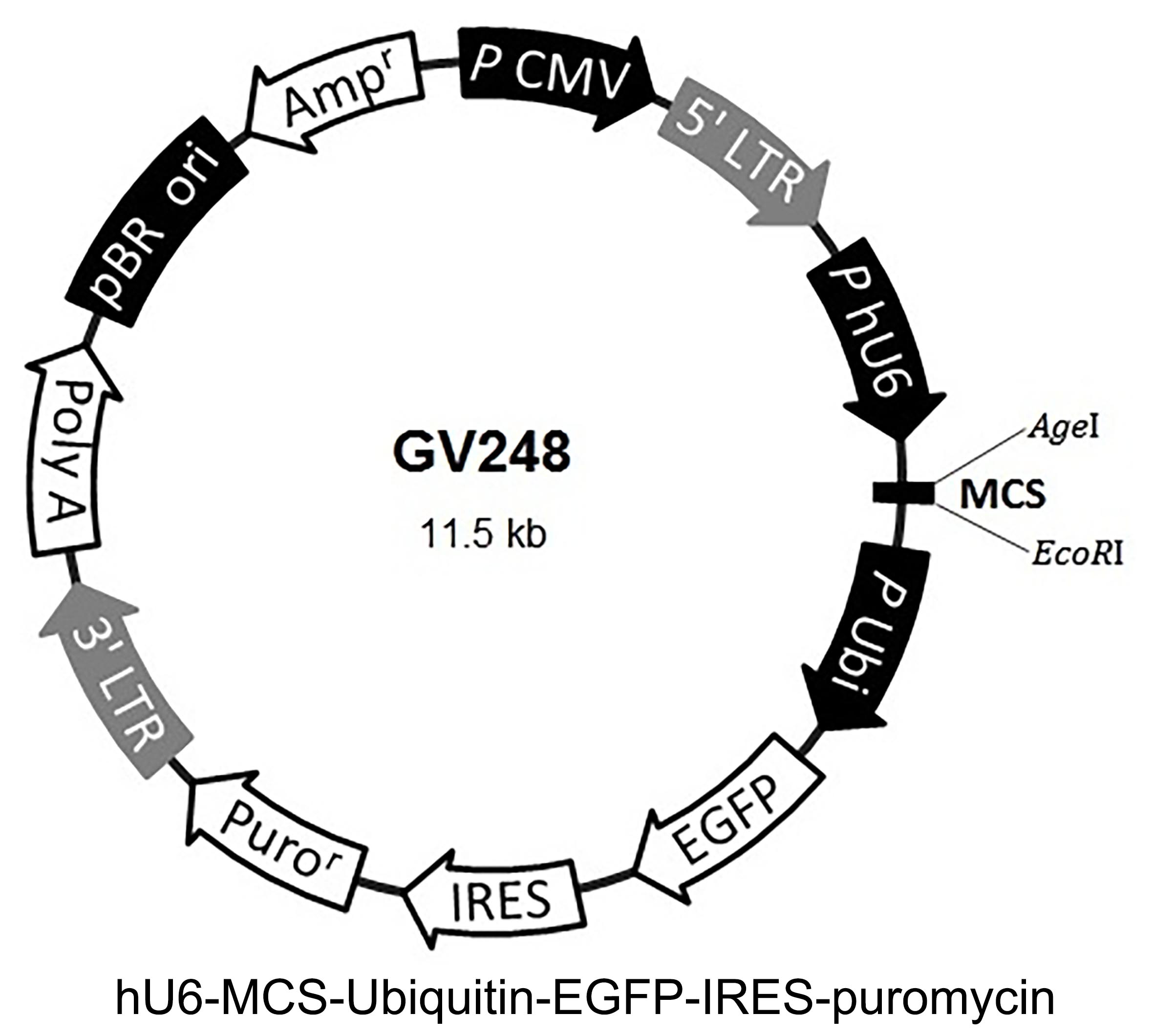

Supplement: Supplementary file 2 — Additional file 2: Figure S1. Structure of SAA1 virus vector: hU6-MCS-Ubiquitin-EGFP-IRES-puromycin. [file 13287_2022_2873_MOESM2_ESM.tif]

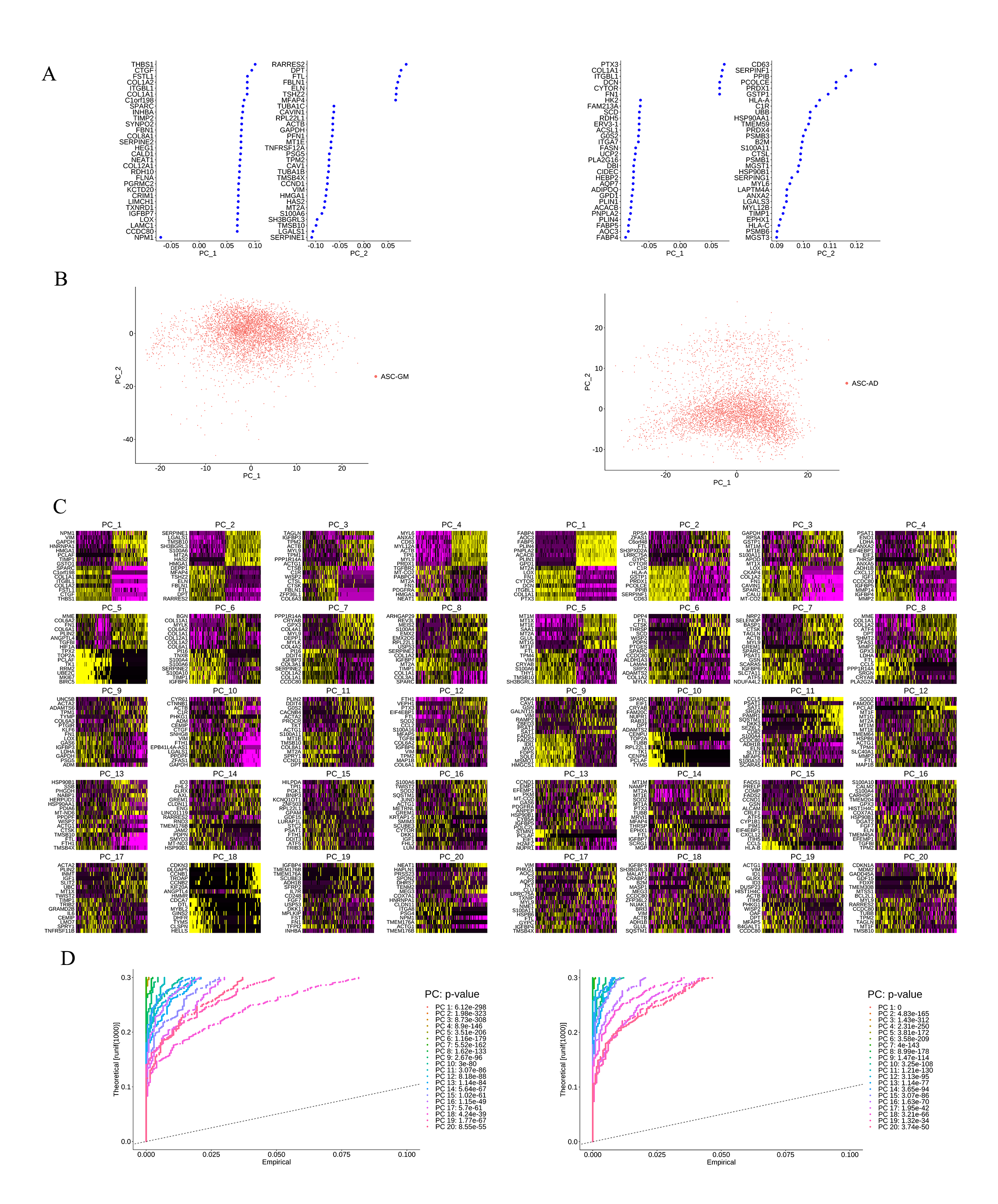

Supplement: Supplementary file 3 — Additional file 3: Figure S2. (A) and (B) show the expression distribution and standard deviation of the top 20 principal components in 100 different types of cells, respectively. (C) and (D) show the expression distribution of the top 20 principal components in 100 different types of cells. [file 13287_2022_2873_MOESM3_ESM.tif]

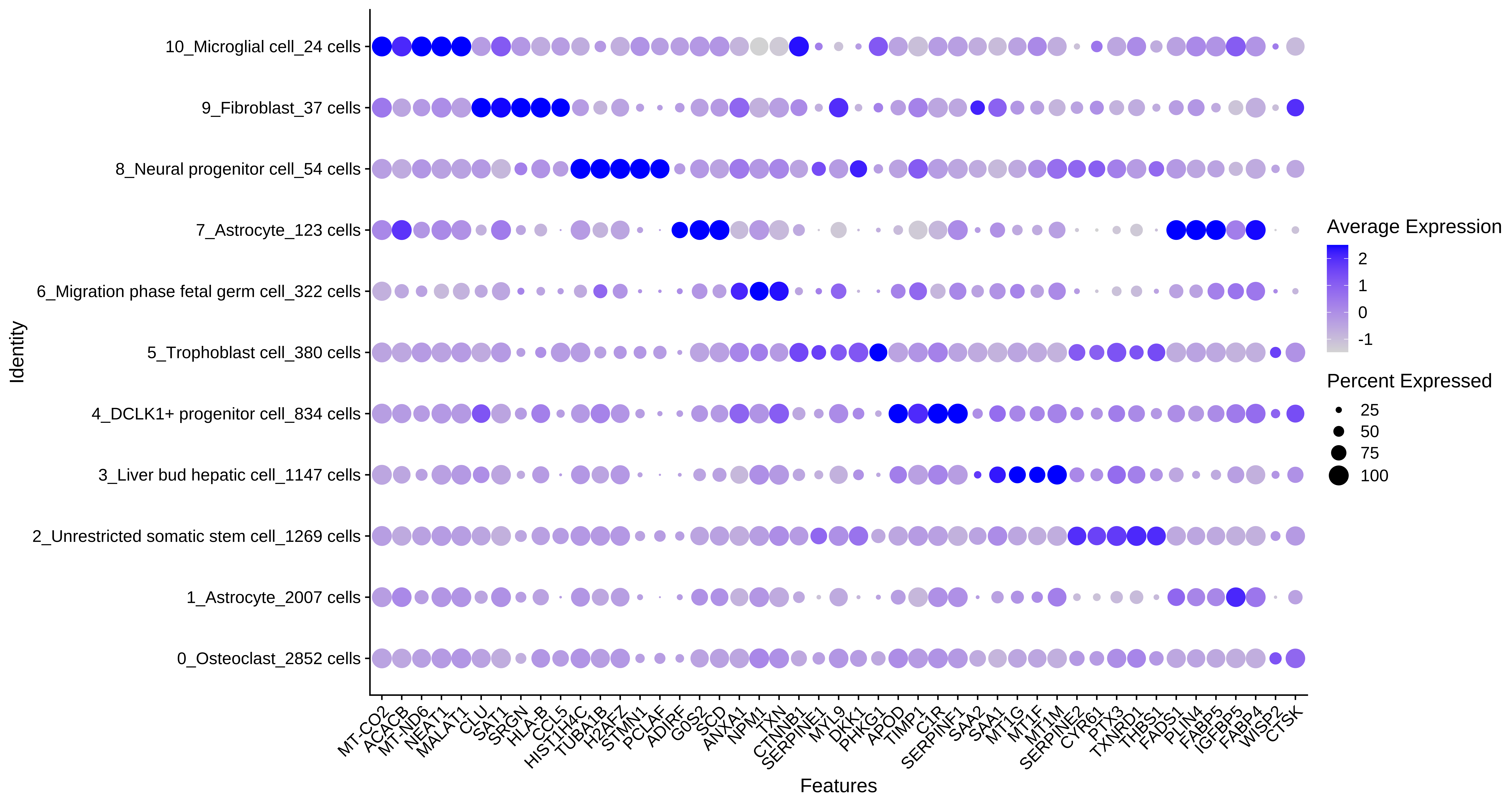

Supplement: Supplementary file 4 — Additional file 4: Figure S3. Bubble diagram of the top 5 tag genes expressed in different subgroups. [file 13287_2022_2873_MOESM4_ESM.tif]
